# Electronic nature of zwitterionic alkali metal methanides, silanides and germanides – a combined experimental and computational approach†
†Electronic supplementary information (ESI) available: Experimental procedures, compound characterization data and computational data are provided. CCDC 926164, 1000501, 1000506, 1000507 and 1013011. For ESI and crystallographic data in CIF or other electronic format see DOI: 10.1039/c6sc02390h



**DOI:** 10.1039/c6sc02390h

**Published:** 2016-10-07

**Authors:** H. Li, A. J. A. Aquino, D. B. Cordes, W. L. Hase, C. Krempner

**Affiliations:** a Texas Tech University , Department of Chemistry and Biochemistry , Box 41061 , Lubbock , Texas 79409-1061 , USA . Email: clemens.krempner@ttu.edu

## Abstract

Zwitterionic group 14 complexes of the alkali metals of formula [C(SiMe_2_OCH_2_CH_2_OMe)_3_M], (M-**1**), [Si(SiMe_2_OCH_2_CH_2_OMe)_3_M], (M-**2**), [Ge(SiMe_2_OCH_2_CH_2_OMe)_3_M], (M-**3**), where M = Li, Na or K, have been prepared, structurally characterized and their electronic nature was investigated by computational methods. Zwitterions M-**2** and M-**3** were synthesized *via* reactions of [Si(SiMe_2_OCH_2_CH_2_OMe)_4_] (**2**) and [Ge(SiMe_2_OCH_2_CH_2_OMe)_4_] (**3**) with MOBu^*t*^ (M = Li, Na or K), resp., in almost quantitative yields, while M-**1** were prepared from deprotonation of [HC(SiMe_2_OCH_2_CH_2_OMe)_3_] (**1**) with LiBu^*t*^, NaCH_2_Ph and KCH_2_Ph, resp. X-ray crystallographic studies and DFT calculations in the gas-phase, including calculations of the NPA charges confirm the zwitterionic nature of these compounds, with the alkali metal cations being rigidly locked and charge separated from the anion by the internal OCH_2_CH_2_OMe donor groups. Natural bond orbital (NBO) analysis and the second order perturbation theory analysis of the NBOs reveal significant hyperconjugative interactions in M-**1**–M-**3**, primarily between the lone pair and the antibonding Si–O orbitals, the extent of which decreases in the order M-**1** > M-**2** > M-**3**. The experimental basicities and the calculated gas-phase basicities of M-**1**–M-**3** reveal the zwitterionic alkali metal methanides M-**1** to be significantly stronger bases than the analogous silanides M-**2** and germanium M-**3**.

## Abstract 2

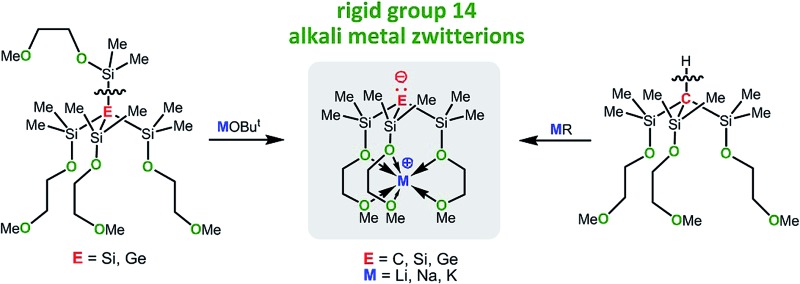
Zwitterionic group 14 complexes of the alkali metals of formula [E(SiMe_2_OCH_2_CH_2_OMe)_3_M], where E = C, Si or Ge and M = Li, Na or K, have been prepared, structurally characterized and their electronic nature was investigated by computational methods.

## Introduction

Carb- and silyl anions are of fundamental importance as synthetic intermediates and as anionic spectator ligands in organic and organometallic/inorganic synthesis. Very recently, significant efforts have been made to the design and synthesis of carb- and silyl anions that contain multiple neutral donor groups as these have proven to be excellent mono-anionic multidentate spectator ligands for main group and transition metal fragments.^1–4^ Anion and neutral donors in these species are covalently connected but electronically largely insulated from each other *via* a linker group.

**Scheme 1:**
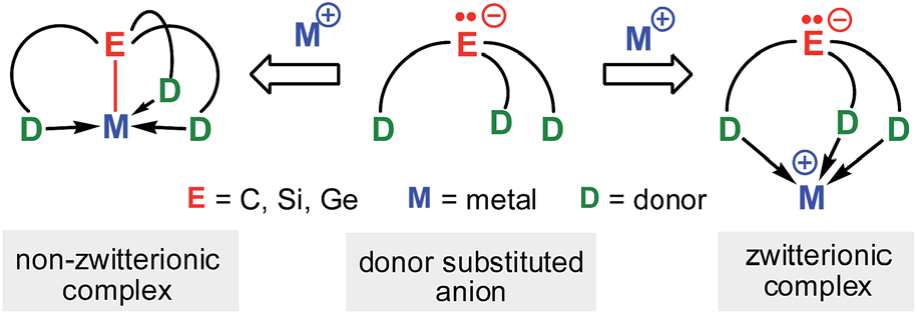
Coordination modes of donor substituted group 14 anions.

The interaction of such a mono-anionic ligand scaffold with a metal cation primarily depends on the chain length of the linker and may be described by two scenarios (Scheme 1); the anion, a strong σ-donor, binds in concert with the neutral donor groups to the metal cation, or the anion is fully charge separated from the metal centre, while the donor groups still coordinate. The latter scenario gives rise to an organometallic zwitterion of tripodal coordination geometry that contains a stereochemically active electron pair localized at the anion, also referred to as a “naked” anion.^5–7^

**Scheme 2:**
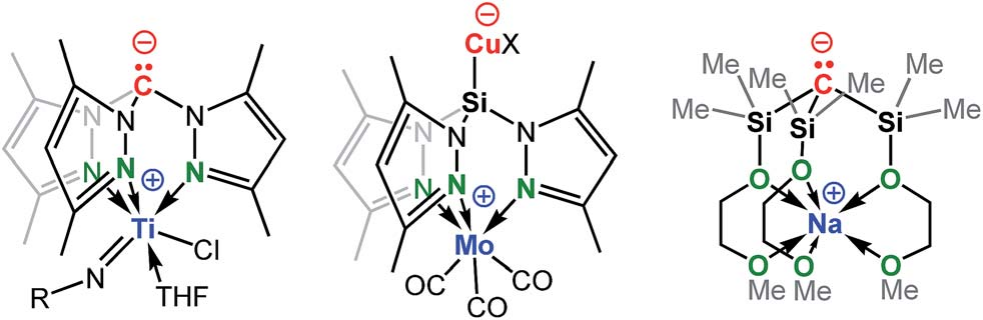
Examples of group 14 complexes that are zwitterionic.

Recent studies have demonstrated the potential of these types of zwitterions to exhibit novel reactivities at both the electrophilic metal cation and the “naked anion” (Scheme 2). For example, Mountford *et al.* reported a unique zwitterionic titanium tris(dimethylpyrazolyl)methanide complex to be a highly productive ethylene polymerization catalyst under commercially-relevant conditions (100 °C, MAO-activation).^5*f*^ Breher's group published the synthesis and electrochemistry of redox-switchable Cu–Mo-hetero-bimetallic zwitterions featuring the tris(dimethylpyrazolyl)silyl anion as the central ligand scaffold.^6*g*^ The quasi-reversible redox process was attributed to the Mo^0^/Mo^I^ redox couple of the metalloligand coordinated to various copper(i) halides. Krempner *et al.* reported the synthesis and structure of a zwitterionic sodium methanide and demonstrated its potential as a base component in Frustrated Lewis Pair (FLP) mediated heterolytic bond-cleavage of H_2_ with weak Lewis acids.^5m,8^ The latter findings led to the introduction of the “inverse” FLP concept, a novel approach that combines weak Lewis acids with strong Brønsted bases with the potential of activating small molecules.

Despite these advances, the chemistry of zwitterionic group 14 complexes is underexplored largely owing to the lack of suitable synthetic methods and the incompatibility of most organic functional groups with highly localized anionic charges. Moreover, only little is really understood in terms of what factors govern the charge density distribution and how to quantify charge separation within a given zwitterionic structure. For a thorough understanding of the fundamental physical and chemical properties and the design of tailor-made zwitterions for catalytic and materials applications further insights are needed particularly with regard to their electronic nature.

**Scheme 3:**
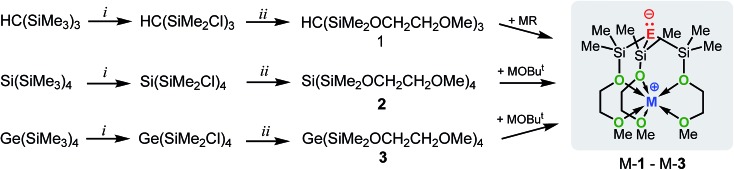
Synthesis of M-**1** (E = C; M = Li, Na or K), M-**2** (E = Si; M = Li, Na or K) and M-**3** (E = Ge; M = Li, Na or K). Conditions: (i) AlCl_3_/AcCl; (ii) HOCH_2_CH_2_OMe/NEt_3_; MR = Bu^*t*^Li, PhCH_2_Na or PhCH_2_K; MOBu^*t*^ (M = Li, Na or K).

In an attempt to address these questions, we report here a combined experimental and computational study on the synthesis, structures and basicities of zwitterions of general formula [E(SiMe_2_OCH_2_CH_2_OMe)_3_M], (M-**1**: E = C; M-**2**: E = Si, M-**3**: E = Ge; M = Li, Na or K) shown in Scheme 3. We envisioned such zwitterions to be ideal model compounds for investigating the impact of charge separation on the “naked” anions reactivity as the alkali metal ions would be rigidly locked and charge separated from the anions by coordinating OCH_2_CH_2_OMe donor groups as we recently demonstrated by the synthesis and structural characterization of the zwitterions M-**2** (M = Li, Na, K)^6*c*^ and Na-**1**.^5*m*^

## Results

### Syntheses and structures

The synthetic approach to M-**1**–M-**3** is illustrated in Scheme 3 and initially involves the formation of the alkoxy-substituted precursor compounds **1–3**.

Chlorodemethylation of HC(SiMe_3_)_3_, Si(SiMe_3_)_4_ and Ge(SiMe_3_)_4_ with AlCl_3_/acetyl chloride in hexanes selectively gave the chlorinated products HC(SiMe_2_Cl)_3_, Si(SiMe_2_Cl)_4_ and Ge(SiMe_2_Cl)_4_, resp., in excellent yields. Subsequent treatment with HOCH_2_CH_2_OMe in the presence of NEt_3_ as base afforded after purification *via* vacuum distillation **1–3** in good to excellent yields as colourless liquids.

Compounds Na-**1** and K-**1**, were generated by deprotonation of **1** with sodium and potassium benzyl, resp., while Li-**1** was prepared *via* deprotonation of **1** with Bu^*t*^Li in hexanes. Recrystallization of M-**1** (M = Li, Na, K) from hexanes gave analytically pure samples. Compounds M-**1** are highly air- and moisture-sensitive solids that readily dissolve in common organic solvents such as hexanes, benzene, THF and ether. Compounds **2** and **3** rapidly undergo selective Si–Si/Si–Ge bond cleavage in the presence of one equiv. of MOBu^*t*^ (M = Li, Na, K) to give M-**2** ^7*b*^ and M-**3** almost quantitatively as judged by ^1^H-NMR spectroscopy. Silanides M-**2** show moderate to good solubilities in hexanes, benzene or toluene, while germanides M-**3** are insoluble in hexanes and only sparingly soluble in benzene and toluene.

**Fig. 1:**
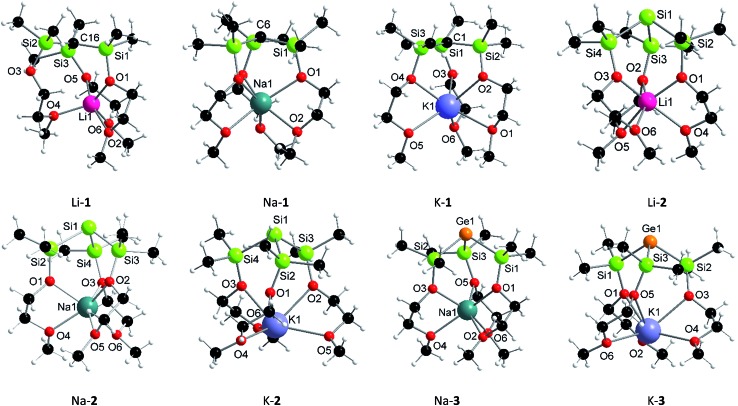
Solid-state structures of Li-**1**, Na-**1**, K-**1**, Li-**2**, Na-**2**, K-**2**, Na-**3** and K-**3** [white = hydrogen, black = carbon].

In addition to being characterized by multi-nuclei NMR spectroscopy and combustion analysis, M-**1**–M-**3** (except Li-**3**), were structurally determined by single X-ray crystallography (Fig. 1). Compounds Li-**2**, Na-**1**–Na-**3** and K-**1** exclusively feature discrete structures with all three pendant donors fully coordinating to the respective cation, whose coordination spheres are best described as distorted octahedral. Contrary, K-**2** and K-**3** feature linear infinite chains in which the monomeric subunits (Fig. 1) are held together *via* intermolecular Si···K [3.66 Å] and Ge···K [3.62 Å] contacts resulting in coordination number 7 for each potassium cation (Fig. S4†). Distinct from all other structures reported herein is carbon analogue, Li-**1**, as lithium in this case is penta-coordinated with only two of the three SiOC oxygen donors coordinating. Temperature dependent ^1^H spectroscopic investigations in solution, however, suggest all three MeOCH_2_CH_2_O donor arms to be equivalent. Even at –80 °C no spectral changes were detected indicating either a fluxional process with an on–off coordination of the MeOCH_2_CH_2_O donor arms or equal binding of all three donor arms to the central lithium cation resulting in a discrete hexa-coordinated species in solution.

### Basicity measurements

The solution basicities of M-**1**–M-**3**, were estimated from their reactions with various weakly acidic hydrocarbons in THF-D_8_ and C_6_D_6_. The progress of these acid–base reactions was monitored by means of ^1^H-NMR spectroscopy. From the integration of the respective ^1^H-NMR signals the conversion and the p*K*_a_ values of the corresponding acids [M-**1** – H]^+^–[M-**3** – H]^+^ was estimated. The results are summarized in Table 1; for clarity only the bases and not their corresponding acids, to which the obtained p*K*_a_ values actually refer, are shown.

**Table 1:** Estimated p*K*_a_ values of M-**1**–M-**3** (M = Li, Na, K) measured in C_6_D_6_ and THF-D_8_

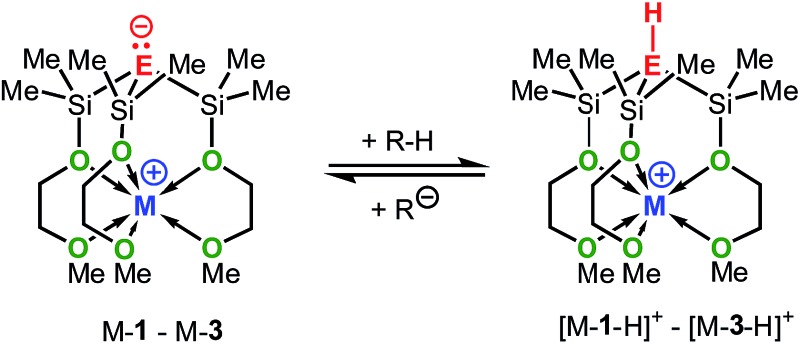		
Base	p*K*_a_ (C_6_D_6_)^*a*^	p*K*_a_ (THF-D_8_)^*a*^
Na-**1**/(2.2.2-cryptand)	—	29.2
K-**1**	22.2	24.5
Na-**1**	22.5	23.6
Li-**1**	23.0	23.6
K-**2**/(2.2.2-cryptand)	≈26 (±1)^*b*^	—
K-**2**	18.7	22.3
Na-**2**	≈16.0^*c*^	18.8
Li-**2**	18.0	19.8
K-**3**/(2.2.2-cryptand)	22.9	—
K-**3**	<16^*d*^	18.0
Na-**3**	<16^*d*^	<16^*d*^
Li-**3**	<16^*d*^	≈16.0^*c*^

^*a*^The calculated p*K*_a_ values refer to the conjugated acids.

^*b*^The p*K*_a_ value was estimated to be 26 ± 1, as no deprotonation of Ph_3_PCH_2_PPh_3_ [p*K*_a(DMSO)_ = 29.9] but 100% deprotonation of 9-Bu^*t*^-fluorene [p*K*_a(DMSO)_ = 24.4] was observed.

^*c*^Approximately 10% deprotonation of 9-Ph-fluorene.

^*d*^No conversion with 9-Ph-fluorene.

Despite many attempts, we were not able to extract accurate and reliable data from the NMR measurements of the germanides M-**3**. In C_6_D_6_ precipitation occurred after partial protonation of M-**3** by 9-PhS-fluorene (p*K*_a(DMSO)_ = 15.5) or 9-C_6_F_5_-fluorene (p*K*_a(DMSO)_ = 14.8) (see also Tables S1 and S2† for more details). Although precipitation could be avoided in THF-D_8_, unidentified secondary reactions or rapid decomposition prevented these compounds from accurately being determined. Nonetheless, that 9-Ph-fluorene was not deprotonated at all by Li-**3**, Na-**3** and K-**3**, while Na-**2** showed at least 10% deprotonation of 9-Ph-fluorene confirmed that germanides M-**3** are weaker bases than silanides M-**2**. Upon adding stoichiometric amounts of 2.2.2-cryptand to Na-**1**, K-**2** and K-**3**, resp., reddish to red-orange solutions were formed in THF-D_8_, while in C_6_D_6_ phase separation occurred with formation of two liquid phases. Notably, these mixtures exhibited much higher basicities (by *ca.* 4–5 p*K*_a_ units) than the corresponding cryptand-free solutions of Na-**1**, K-**2** and K-**3**.

**Scheme 4:**
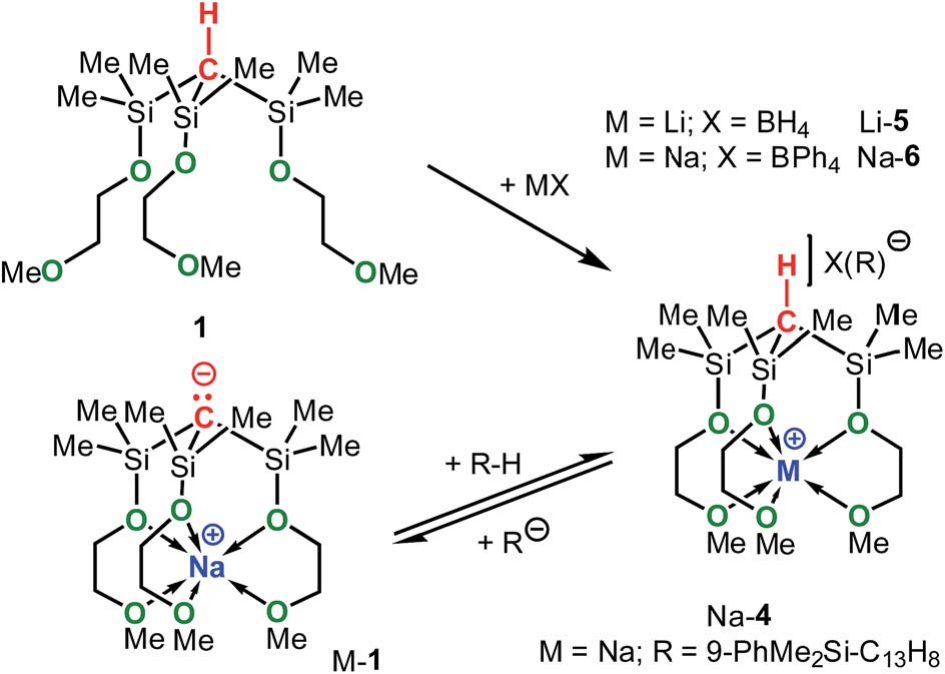
Synthesis of Na-**4**, Li-**5** and Na-**6**.

**Fig. 2:**
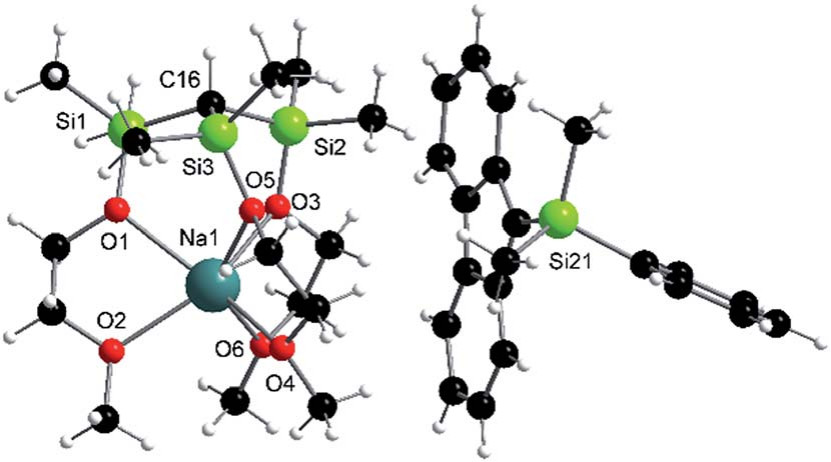
Solid-state structure of Na-**4**: selected average distances [Å] and angles [°]: Si–C, 1.87; Si–O, 1.66; Na–O(Si), 2.40; Na–O(C), 2.36; Si–C–Si, 113; Si–O–Na, 121; C–Si–O–Na, 35.

It should be mentioned that the solution basicities of M-**1**–M-**3** are estimated based on the assumption that the metal ion upon protonation of the zwitterion remains fully coordinated by the MeOCH_2_CH_2_O donor arms resulting in the formation of the cationic acids [M-**1** – H]^+^–[M-**3** – H]^+^ (Table 1). This notion is supported by the NMR spectroscopic characterization and the results of an X-ray analysis of salt Na-**4** (Scheme 4 and Fig. 2) prepared from the reaction of Na-**1** with PhMe_2_Si-fluorene, in C_6_D_6_. Further evidence is provided by the characterization of salts Na-**6** and Li-**5** prepared from reactions illustrated in Scheme 4.

### DFT calculations

To investigate the electronic nature of M-**1**–M-**3**, geometry optimizations on M-**1**–M-**3** and their conjugated acids [M-**1** – H]^+^–[M-**3** – H]^+^ (Table 2) have been carried out at the DFT/B3LYP/TZVP level of theory (Turbomole program suite). From the fully optimized gas-phase structures (see ESI for more details†) the HOMO energies, enthalpies (*H*) and Gibbs free energies (*G*) were calculated. The gas-phase basicities (GPBs) and proton affinities (PAs) of M-**1**–M-**3** were also calculated.^9^

**Table 2:** Calculated proton affinities, PA, [kcal mol^–1^], gas-phase basicities, GPB, [kcal mol^–1^], HOMO energies [eV] and selected NPA charges, *e*, [E^–^] of M-**1**–M-**3** (M = Li, Na, K), **1^–^–3^–^**, [M-**1** – H]^+^–[M-**3** – H]^+^, **1**, **2**-H and **3**-H

Comp.	*E*_HOMO_	*e*_(M^+^)_	*e*_(E^–^)_	Δ*e*_(E^–^)_ ^*a*^	GPB^*c*^	PA^*d*^	Comp.	*e*_(M^+^)_	*e*_(E)_
**1^–^**	–1.439	—	–2.188	–0.422^*e*^	–350.2	–351.7	**1**	—	–1.766
K-**1**	–4.256	+0.949	–2.189	–0.420	–280.6	–289.2	[K-**1** – H]^+^	+0.953	–1.769
Na-**1**	–4.335	+0.917	–2.168	–0.399	–282.3	–290.7	[Na-**1** – H]^+^	+0.918	–1.769
Li-**1** ^*b*^	–4.415	+0.899	–2.187	–0.413	–282.7	–291.9	[Li-**1** – H]^+^	+0.896	–1.774
**2^–^**	–1.111	—	–0.807	–0.601^*f*^	–339.7	–351.6	**2**-H	—	–0.206
K-**2**	–3.837	+0.945	–0.738	–0.567	–278.9	–286.3	[K-**2** – H]^+^	+0.950	–0.171
Na-**2**	–4.013	+0.913	–0.709	–0.556	–276.3	–283.8	[Na-**1** – H]^+^	+0.917	–0.153
Li-**2**	–4.200	+0.891	–0.683	–0.544	–274.0	–281.4	[Li-**2** – H]^+^	+0.898	–0.139
**3^–^**	–1.202	—	–0.770	–0.550^*g*^	–334.9	–344.3	**3**-H	—	–0.220
K-**3**	–3.914	+0.945	–0.698	–0.519	–272.7	–280.5	[K-**3** – H]^+^	+0.949	–0.179
Na-**3**	–4.082	+0.912	–0.665	–0.507	–269.1	–277.8	[Na-**3** – H]^+^	+0.917	–0.158
Li-**3**	–4.260	+0.890	–0.634	–0.493	–267.5	–276.4	[Li-**3** – H]^+^	+0.899	–0.141

^*a*^Δ*e*_(E^–^)_ = *e*_(E^–^)_ (base) – *e*_(E)_ (conjugated acid).

^*b*^Values for Li-**1** are Boltzmann averaged.

^*c*^ *G*_(H^+^)_ = –6.28 kcal mol^–1^, taken from ref. 12.

^*d*^ *H*_(H^+^)_ = 1.48 kcal mol^–1^, taken from ref. 12.

^*e*^From *e*_(E^–^)_ (**1^–^**-A) – *e*_(E)_ (**1**-A).

^*f*^From *e*_(E^–^)_ (**2^–^**-A) – *e*_(E)_ (**2**-H-A).

^*g*^From *e*_(E^–^)_ (**3^–^**-A) – *e*_(E)_ (**3**-H-A).

With the exception of Li-**1**, all calculated gas-phase structures feature discrete units with an octahedral coordination environment for the central metal cation, similar to what is seen for most structures in the solid state. For Li-**1**, two local minima were found, tetra-coordinated Li-**1**-A, which somewhat resembles the X-ray structure of Li-**1**, and Li-**1**-B, which is penta-coordinated and only slightly higher in energy than Li-**1**-A (Fig. S5 and S6†). For a comparative discussion of the computational results, Li-**1**-A and Li-**1**-B were sampled with Boltzmann-weighed averaging; only the averaged results are shown in Table 2.

**Scheme 5:**
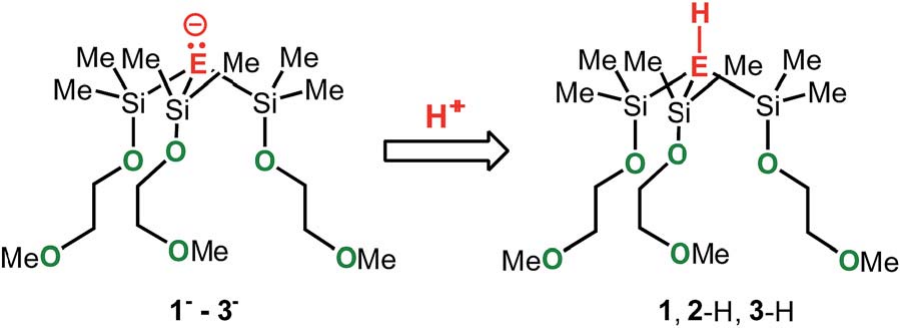
Structures of the metal free species **1^–^** (E = C), **2^–^** (E = Si), **3^–^** (E = Ge), **1** (E = C), **2**-H (E = Si), and **3**-H (E = Ge).

To elucidate the electronic impact of the metal on the naked anion's basicity, the metal-free anions **1^–^–3^–^** along with the respective conjugated acids **1**, **2**-H and **3**-H were calculated. The gas-phase basicities (GPBs) and proton affinities (PAs) were also calculated (Scheme 5). Two local minima markedly different in energy (denoted as A and B) were found for each of the structures **1^–^–3^–^**, **1**, **2**-H and **3**-H (see also Tables S15 and S16†). In the A conformers the pendant donor groups pointing inwards (largely in the same direction) similar to what is seen in the zwitterionic species. The B conformers have at least one donor arm pointing in the opposite direction, caused by rotation of the central E–Si bond (see also Fig. S7–S18†). The PAs and GPBs of **1^–^–3^–^** were calculated from the most stable conformers.

The natural bond orbital (NBO) analysis was performed with the optimized structures of the zwitterions Na-**1**, Na-**2** and Na-**3** (Table 3) at the B3LYP/def2-TZVP level of theory using Gaussian 9. The hybridization of the anionic carbon, C^–^, in Na-**1** can be described as being sp^2^ and the lone pair (LP) as p. Similarly, the anionic silicon, Si^–^ in Na-**2** is sp^2^ and the lone pair (LP) is p.^10^ In contrast, the anionic germanium, Ge^–^, in Na-**3** has strong p-character (87%), while the lone pair (LP) has strong s character (63%). Note also that the Si–C bond in Na-**1** is polar (73% C and 27% Si), while the polarities for Si–Si bond in Na-**2** and Si–Ge bond in Na-**3** were found to be about equal.

**Table 3:** NBO analysis (B3LYP/def2-TZVP) of Na-**1**, Na-**2** and Na-**3** with bond polarity (% Si, % E; E = C, Si, Ge), occupancy of the NBO's and hybridization of atoms (in % AO) and the lone pairs (LP)

Comp.	Bond	Occup.	Energy (au)	% E	% Si	AO % E			AO % Si		
						s	p	d	s	p	d
Na-**1**	E(LP)	1.676	–0.10925	—	—	0.54	99.45	0.01	—	—	—
E = C	E–Si	1.952	–0.49102	73.36	26.64	33.17	66.77	0.06	32.55	67.09	0.36
Na-**2**	E(LP)	1.452	–0.07666	—	—	0.96	98.96	0.08	—	—	—
E = Si	E–Si	1.923	–0.37829	51.51	48.49	32.98	66.55	0.46	30.06	69.50	0.44
Na-**3**	E(LP)	1.826	–0.31741	—	—	62.75	37.05	0.20	—	—	—
E = Ge	E–Si	1.910	–0.34554	47.70	52.30	12.41	87.01	0.57	30.71	69.04	0.25

Calculations of the NPA (Natural Population Analysis) charges^11^ of M-**1**–M-**3**, **1^–^–3^–^**, **1**, **2**-H, **3**-H and [M-**1** – H]^+^–[M-**3** – H]^+^ (Table 2) have been carried out from their fully optimized gas-phase structures. The NPA charges of the central anion, *e*_(E^–^)_, of M-**1** and **1^–^** range from –2.17 to –2.19. These values are approximately three times larger than those of the silanides and germanides M-**2**, M-**3**, **2^–^** and **3^–^** ranging from –0.63 to –0.81.

Given the relatively low-lying HOMOs of M-**1**, the NPA charges appear to be unreasonably high. Carbon, however, is significantly more electronegative than Ge and Si. As a result the central ^–^C(Si)_3_ framework is more polar compared to the central ^–^Si(Si)_3_ and ^–^Ge(Si)_3_ units leading to a markedly higher NPA charge for carbon.^13^ Note also that the calculated NPA charges of the central carbon of the conjugated acids [M-**1** – H]^+^ have high negative values, ranging from –1.76 to –1.69*e*, despite their overall charge being +1. Thus, two major factors appear to contribute to the calculated NPA charges of E^–^ (E = C, Si, Ge), the electronegativity difference of the three Si–E^–^ bonds and the lone pair at E^–^. To eliminate these electronegativity effects the NPA charge of E of the conjugated acid, *e*_(E)_, was subtracted from that of the anion E^–^ (see Table 2), according to the following equation:Δ*e*_(E^–^)_ = *e*_(E^–^)_ (base) – *e*_(E)_ (conjugate acid)

Accordingly, low Δ*e*_(E^–^)_ values indicate a higher degree of delocalization of the lone pair, while values close to –1 indicate the electron pair to be largely localized at E^–^. In the discussion section only the Δ*e*_(E^–^)_ values will be used as a relative measure of the electron pair density at E^–^.

## Discussion

### Synthetic aspects

**Scheme 6:**
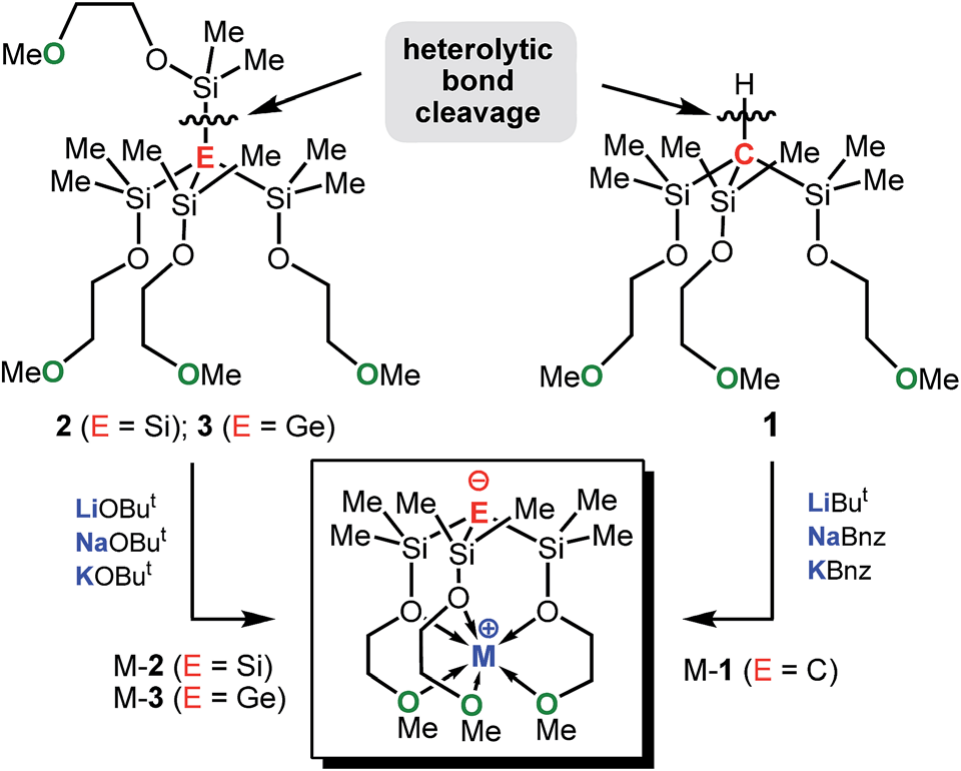
Synthetic approach to M-**1**–M-**3**.

The KOBu^*t*^-induced heterolytic Si–Si bond-cleavage, first discovered by Buncel *et al.* for phenyl-substituted disilanes^14^ and later expanded by Marschners group to a multitude of branched oligosilanes and silylgermanes^15^ represents one of the most powerful synthetic methods of selectively generating potassium silanides and germanides. This methodology can conveniently be applied to the less basic alkoxides LiOBu^*t*^ and NaOBu^*t*^ and even to earth alkaline metal alkoxides, allowing access to various donor substituted alkali and alkaline earth metal silanides.^6a,c,d^ Remarkable is the high regio-selectivity with which the Si–Si and Si–Ge bonds are cleaved heterolytically regardless of the metal alkoxide used. This highly efficient synthetic protocol enables quick and easy access to the zwitterions M-**2** and M-**3** in almost quantitative yields; *in situ* generated solutions of M-**2** and M-**3** can be used for subsequent chemistry without the need for further purification (Scheme 6). Since C–Si bonds are usually too strong to be cleaved heterolytically by alkali metal alkoxides, deprotonation of trisilyl-substituted carbosilanes of formula HC(SiR_3_)_3_ was the most promising synthetic strategy to introduce the metal cation.^16^ In fact, deprotonation of the carbosilane **1** proceeded selectively providing access to the zwitterionic alkali metal methanides M-**1** in good isolated yields. In particular the use of PhCH_2_Na and PhCH_2_K as base proved to be efficient in allowing potassium and sodium ions to be incorporated into the zwitterionic framework (Scheme 6).

### Bond parameters

We analysed the structural parameters of the central negatively charged ^–^ESi_3_ framework of M-**1**–M-**3** using the available X-ray data. Table 4 shows the average E–Si distances and Si–E–Si angles of M-**1**–M-**3** along with those of the structurally related compounds of formula (Me_3_Si)_3_EM(donor)_*n*_, available from the Cambridge Crystallographic Database.

**Table 4:** Average E–Si distances [Å] and Si–E–Si angles [°] of [(Me_3_Si)_3_E][M(donor)_*n*_] (E = C, Si, Ge; M = Li, Na, K) and M-**1**–M-**3** [M = Li, Na, K]

Compounds	C–Si	Si–C–Si
(Me_3_Si)_4_C	1.925	109.5
(Me_3_Si)_3_CH	1.888	114.4
(Me_3_Si)_3_CM(donor)_*n*_ ^*a*^	1.825	115.3
C(SiMe_2_OCH_2_CH_2_OMe)_3_M (M-**1**)	1.790	118.0

**Compounds**	**Si–Si**	**Si–Si–Si**
(Me_3_Si)_4_Si^*a*^	2.346	109.4
(Me_3_Si)_3_SiM(donor)_*n*_ ^*a*^	2.336	101.5
Si(SiMe_2_OCH_2_CH_2_OMe)_3_M (M-**2**)	2.320	98.0

**Compounds**	**Ge–Si**	**Si–Ge–Si**
(Me_3_Si)_4_Ge^*a*^	2.370	109.5
(Me_3_Si)_3_GeM(donor)_*n*_ ^*a*^	2.376	101.1
Ge(SiMe_2_OCH_2_CH_2_OMe)_3_M (M-**3**)	2.370	97.5

^*a*^Average distances and angles were calculated from relevant structures found in the Cambridge Crystallographic Database with experimental *R*-values ≤7.

Analysis of these bond parameters revealed that the zwitterionic methanides M-**1** are different from their silicon and germanium analogues M-**2** and M-**3**. Despite the presence of an electron pair, the central anionic CSi_3_-framework of M-**1** is nearly trigonal planar, while the SiSi_3_ and GeSi_3_ units of M-**2** and M-**3** are pyramidal with average Si–E–Si angles clearly below the ideal tetrahedral angle. As expected, similar trends are seen for the non-zwitterionic species (Me_3_Si)_3_EM(donor)_*n*_ and [Ph_3_E][K(18-crown-6)] (E = C, Si, Ge). Thus, the silyl-substituted carbanions, (Me_3_Si)_3_C^–^, adopt relatively large average Si–C–Si angles of *ca.* 115° and the resonance-stabilized phenyl-substituted carbanions, Ph_3_C^–^, are almost ideally trigonal planar, while the silyl and germyl anions, (Me_3_Si)_3_Si^–^, (Me_3_Si)_3_Ge^–^, Ph_3_Si^–^ and Ph_3_Ge^–^, exhibit strong pyramidalization.

Of further note are the average C–Si distances [1.790 Å] of M-**1**, which are significantly shortened (>5%) relative to those of carbosilanes, (Me_3_Si)_3_CH and (Me_3_Si)_4_C.^17^ Both, shortening of the C–Si distances and planarization of the central CSi_3_-skeleton of M-**1** can be attributed to the acceptor properties of the silyl groups capable of electronically interacting with the lone pair of the carbanion through negative hyperconjugation.^18^ Both the zwitterionic silanides and germanides M-**2** and M-**3** seem to largely lack such stabilizing interactions as reflected in a strong pyramidalization of the central anions as well as insignificant changes in the average Si–Si- and Ge–Si-distance of the central ^–^ESi_3_ framework relative to those of (Me_3_Si)_4_Si and (Me_3_Si)_4_Ge, resp. On the other hand, the average Si–C [1.94 Å] and Ge–C [2.02 Å] distances of the triphenyl-substituted anions, Ph_3_Si^–^, and Ph_3_Ge^–^, are significantly longer than those of their conjugated acids, Ph_3_SiH [1.85 Å] and Ph_3_GeH [1.95 Å], respectively.^19^ Considering such an elongation to be expected for group 14 anions that lack stabilization by resonance or negative hyperconjugation, it seems reasonable to assume that even zwitterions M-**2** and M-**3** are somewhat stabilized by negative hyperconjugation. The decrease of these stabilizing interactions in the order C > Si > Ge is also evident from comparing the results of the gas-phase geometry optimizations of the zwitterions M-**1**–M-**3** with those of their conjugated acids [M-**1** – H]^+^ and [M-**3** – H]^+^, resp. (Tables S6, S9 and S12†). Similar structural trends are seen for the aforementioned compounds (Me_3_Si)_3_EM(donor)_*n*_ (Table 3). The Si–E bond shortening of the anionic units (Me_3_Si)_3_E^–^ (E = C, Si), however, is less pronounced compared to M-**1** and M-**2**, which can be attributed to the absence of the electron-withdrawing OCH_2_CH_2_OMe groups in the former compounds.

### ^–^E···M^+^ charge separation

The calculated and experimentally observed E···M distances of M-**1**–M-**3** together with those of the structurally related compounds of general formula (Me_3_Si)_3_EM(donor)_*n*_ are listed in Table 5. In the latter species, the metal cations are in close proximity to their counter anions owing to attractive electrostatic cation–anion interactions, and the E–M distances are primarily a function of the size of cation and anion as well as the coordination number of the metal cation. On the contrary, the intramolecular E–M distances of zwitterions M-**1**–M-**3** are significantly elongated ranging from 3.03 Å (C···Li) for Li-**1** (calc.) up to 4.14 Å (Ge···K) for K-**3** (calc.), clearly confirming the zwitterionic nature of these highly reactive species. In an attempt to quantify the degree of charge separation in these zwitterions, the calculated E···M distances of M-**1**–M-**3**, which except for Li-**1** have all hexa-coordinated metal cations were taken and the percentage elongation toward the E–M distance of (Me_3_Si)_3_EM was calculated (Table 5).

**Table 5:** M–E distances [Å] of M-**1**–M-**3** (from X-ray data and DFT calculations) and (Me_3_Si)_3_EM(donor)_*n*_ [E = C, Si, Ge; M = Li, Na, K] (from the Cambridge Crystallographic DataBase)

Comp.	M···E (X-ray)	M···E (calc.)	Comp.	M–E (X-ray)^*a*^	Elongation^*b*^ [%]
Li-**1**	3.30	3.03	(Me_3_Si)_3_CLi	2.13	42
Na-**1**	3.23	3.18	(Me_3_Si)_3_CNa	2.48	28
K-**1**	3.32	3.34	(Me_3_Si)_3_CK	2.92	14
Li-**2**	3.92	3.74	(Me_3_Si)_3_SiLi	2.52	48
Na-**2**	4.00	3.94	(Me_3_Si)_3_SiNa	2.94	34
K-**2**	4.50	4.07	(Me_3_Si)_3_SiK	3.34	22
Li-**3**	—	3.88	(Me_3_Si)_3_GeLi	2.62	48
Na-**3**	4.05	4.01	—^*c*^	—	—
K-**3**	4.53	4.14	(Me_3_Si)_3_GeK	3.37	23

^*a*^Values are taken from the Cambridge Crystallographic Database and only those structures with the shortest M–E bonds were selected.

^*b*^Percent elongation of the E–M distance of M-**1**–M-**3**, derived from DFT from calculations, toward the E–M distance of (Me_3_Si)_3_EM, derived from the X-ray data.

^*c*^No X-ray data available.

From these data it seems that the elongation of the E–M distance is the largest for zwitterionic structures with lithium as cation. Of the anions, germanium seems to provide the highest degree of charge separation. Interestingly, the latter appears to correlate with the solubilities of M-**1**–M-**3** in organic solvent as Li-**3** (elongation 48%) shows the lowest solubility and is essentially insoluble in hexanes, while K-**1** (elongation 14%) is well soluble in hexanes.

### The anion, E^–^

**Fig. 3:**
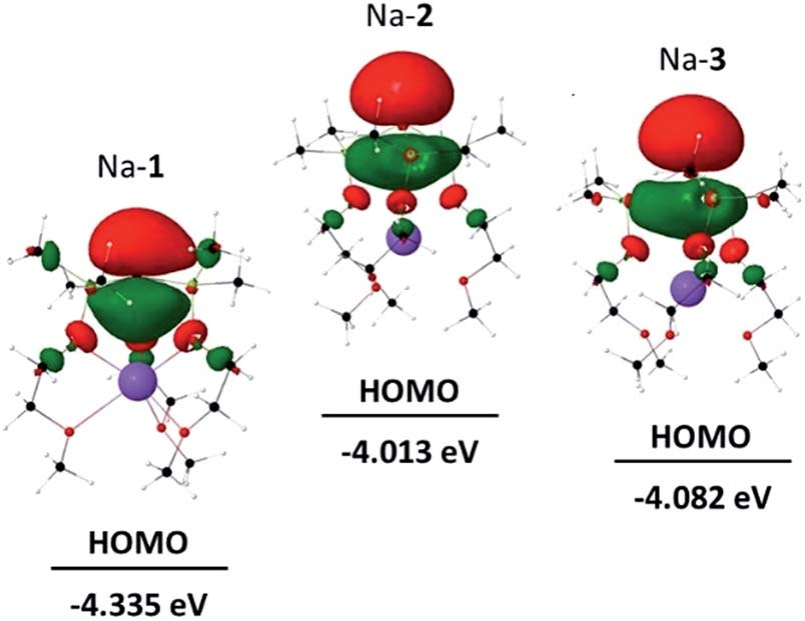
HOMO of Na-**1**, Na-**2** and Na-**3**.

**Fig. 4:**
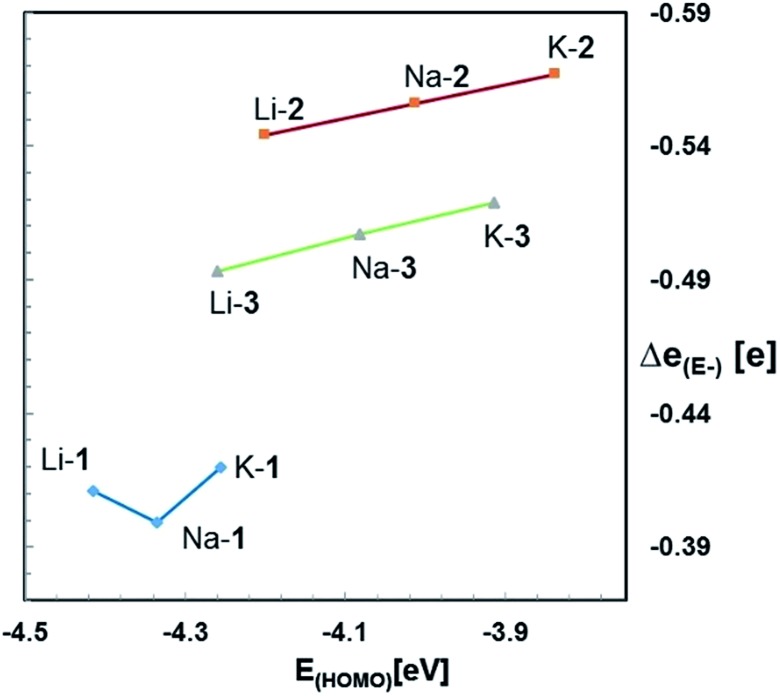
Plot of *E*_HOMO_ *versus* NPA charges [Δ*e*_(E^–^)_] for M-**1**–M-**3** (M = Li, Na, K).

Fig. 3 shows the HOMOs of Na-**1**–Na-**3**, as representative examples of all zwitterions. The HOMOs largely reside at the formally negatively charged atoms E^–^, regardless of its identity. These findings are consistent with reports from the Breher group, who found the HOMOs of the zwitterions [C(1,3-Me_2_-pyrazolyl)_3_Li × THF] and [Si(1,3-Me_2_-pyrazolyl)_3_Li]_∞_ also to reside at the anions.^6*b*^ Furthermore, the HOMO energies of each series of zwitterions correlate reasonably well with the NPA charges of their central anions, E^–^ (Fig. 4). Both numerical values increase progressively from Li to Na and K, independent of the identity of anion, E^–^. An exception is Li-**1**, the only structure in which the metals coordination number deviates from 6.

Note that the HOMO energies as well as the NPA charges [Δ*e*_(E^–^)_] of E^–^ of M-**2** and M-**3** also seem to correlate with the size of the counter-cation; that is the smaller the size of M^+^ the lower the HOMO energy of the respective zwitterion and its NPA charge, Δ*e*_(E^–^)_. We rationalize this in terms of an electronic communication between cation and anion occurring primarily through the pendant donor groups. The lithium cation, the smallest of the series more strongly polarizes the donating oxygen of the pendant donor groups than sodium and potassium. This in turn further strengthens the (Si)O→M dative bond interaction, and thereby further lowers both, the negative charge of E^–^ and the energy of the HOMO. In simple terms, the smaller lithium cation pulls slightly more electron density away from E^–^ than sodium and potassium. Such a conclusion appears to be consistent with the charge distribution in the metal free anions **1^–^**-A, **2^–^**-A, and **3^–^**-A, which lack such interactions, and therefore exhibit slightly higher negative NPA charges of the anion E^–^ than those of the metal-containing zwitterions M-**1**–M-**3**, resp.

### Hyperconjugation

α-Trialkylsilyl-substituted carbanions are stabilized by donor–acceptor interactions between the lone pair p-orbital of the carbanion and the relatively low lying σ*-orbitals of the Si–alkyl bonds. The extent of these so-called negative hyperconjugative interactions is a sensitive function of the LP-C–Si–CH_3_ torsion angle and the C–Si distance. Optimal overlap between the LP p-orbital and the antibonding Si–C orbital occurs in a coplanar conformation; any deviation from the latter weakens these interactions.

**Fig. 5:**
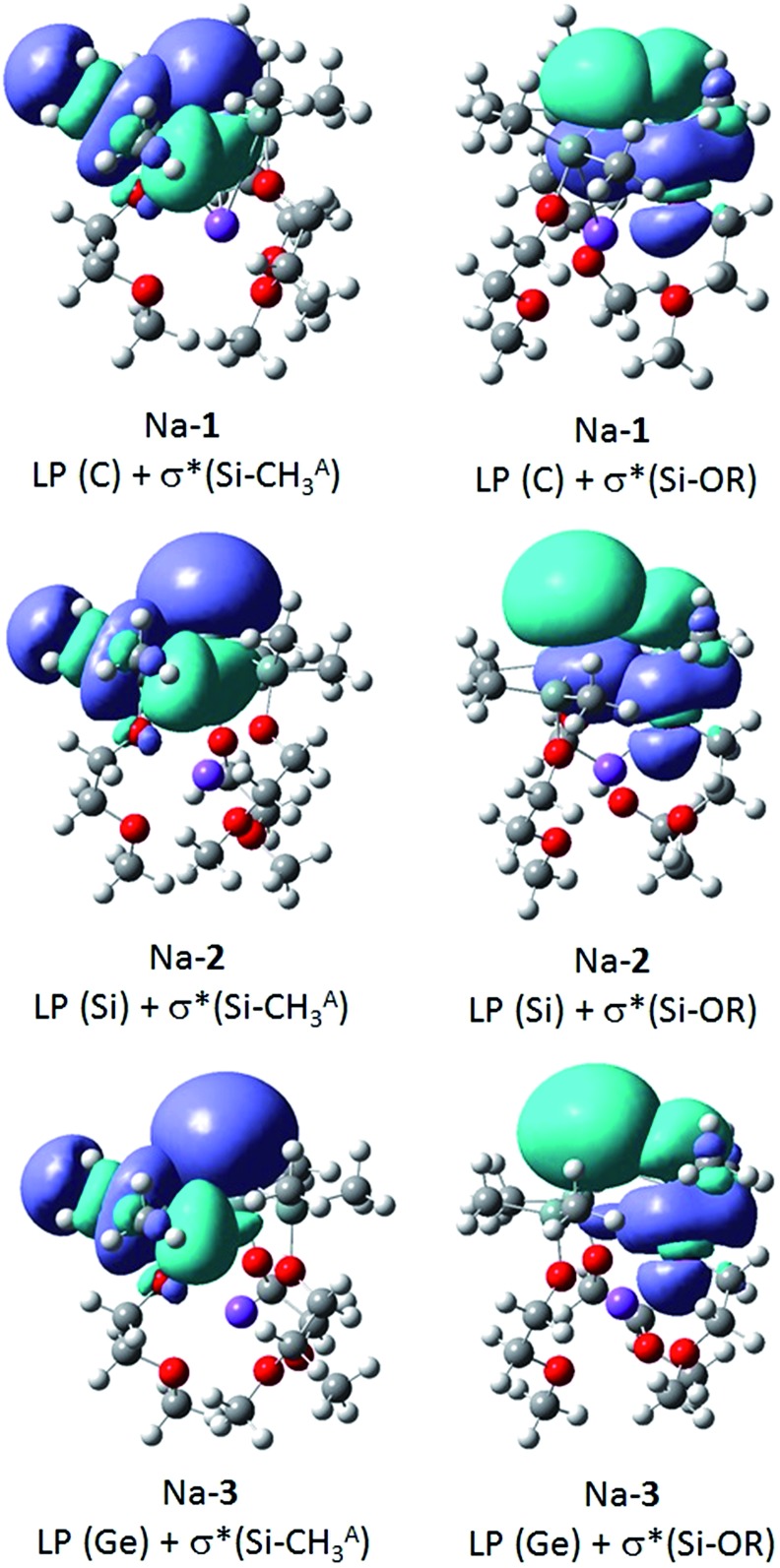
Donor–acceptor interaction of the LP (E) with the antibonding Si–CHA3 and Si-OR orbitals of Na-**1**, Na-**2** and Na-**3**.

To identify negative hyperconjugative interactions in our zwitterions, the second order perturbation theory analysis of the NBO's were carried out for Na-**1**, Na-**2** and Na-**3**. The results shown in Table 6 and Fig. 5 demonstrate the conformational dependence of hyperconjugative interactions in these molecules.

**Table 6:** Stabilization energies of selected donor–acceptor interactions derived from the second order perturbation analysis of the NBO's of Na-**1**, Na-**2** and Na-**3**

Comp.	Donor NBO	Acceptor NBO	Energy [kcal mol^–1^]	Torsion angle^*b*^ [°]
Na-**1**	LP (C)	σ* (Si–CHA3)	5.0	45
	LP (C)	σ* (Si–CHB3)	—^*a*^	80
	LP (C)	σ* (Si-OR)	14.9	165
Na-**2**	LP (Si)	σ* (Si–CHA3)	1.2	50
	LP (Si)	σ* (Si–CHB3)	—^*a*^	70
	LP (Si)	σ* (Si-OR)	11.2	170
Na-**3**	LP (Ge)	σ* (Si–CHA3)	—^*a*^	45
	LP (Ge)	σ* (Si–CHB3)	—^*a*^	75
	LP (Ge)	σ* (Si-OR)	6.6	165

^*a*^Below the threshold of 0.5 kcal mol^–1^.

^*b*^Estimated torsion angles for LP-E–Si–CH_3_ or LP-E–Si–OR, where E = C, Si or Ge and LP = lone pair.

**Fig. 6:**
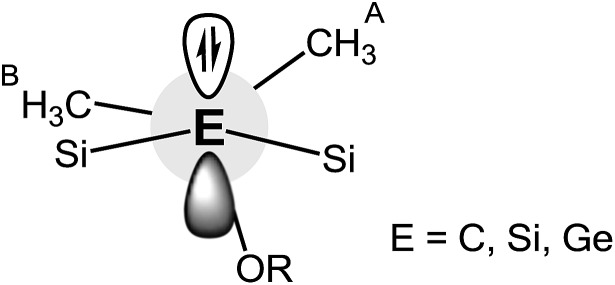
Representation of the conformational arrangement around the central Si–E^–^ bond in Na-**1**, Na-**2** and Na-**3**.

Thus, the stabilization energy for the donor–acceptor interaction of the LP (C) with the three antibonding Si–CHB3 orbitals in Na-**1** is below the threshold of 0.5 kcal mol^–1^, because the CHB3 groups are nearly perpendicularly (torsion angles *ca.* 80°) oriented towards the LP (Fig. 6). The three CHA3 groups, on the other hand, are oriented towards the LP nearly gauche (torsion angles *ca.* 45°), which results in a notable stabilization of *ca.* 5 kcal mol^–1^ for each CHA3 group in Na-**1**. In contrast, the stabilization energy for Na-**2** is only 1.2 kcal mol^–1^ for each CHA3 group and that for Na-**3** is below the threshold of 0.5 kcal mol^–1^, which can be attributed to the significantly longer Si–Si^–^ and Si–Ge^–^ bond lengths in Na-**2** and Na-**3**, resp., as compared to that of Si–C^–^ in Na-**1**.

The strongest donor–acceptor interactions, however, are seen with the three antibonding Si-OR orbitals. The oxygen is nearly in an anti-periplanar orientation relative to the LP (torsion angles of 165–170°), which strengthens orbital overlap. Note also that the stabilization energy resulting from this hyperconjugative interaction decreases in the order Na-**1** > Na-**2** > Na-**3**, again due to an increase of the central Si–E^–^ distance in the order Na-**1** < Na-**2** < Na-**3**.

Taken all together, negative hyperconjugative interactions in Na-**1** are most pronounced with an estimated overall stabilization energy of *ca.* 60 kcal mol^–1^. These interactions cause the central Si_3_C^–^ framework to be almost planar and release some of the negative charge at the central carbanion, C^–^. Hyperconjugation in Na-**2** and Na-**3** with overall stabilization energies of 37 kcal mol^–1^ and 20 kcal mol^–1^, respectively, is significantly weaker causing the charges to be largely localized Si^–^ and Ge^–^ and the geometry of the central Si_3_Si^–^ and Si_3_Ge^–^ units to be pyramidal.

### The metal cation, M^+^

The metal cations NPA charges, *e*_(M^+^)_, of all zwitterionic structures, M-**1**–M-**3**, (Table 2) are very similar to each other, and only slightly increase in the order Li (0.89–0.90) < Na (0.90–0.91) < K (0.95) regardless of the nature of the anion. Upon protonation of M-**1**–M-**3** to [M-**1** – H]^+^–[M-**3** – H]^+^ the NPA charges, *e*_(M^+^)_, only marginally differ, most of them slightly increase. In other words, the degree of charge separation as well as the nature of anion, E^–^, do not seem to affect the metals charge. Therefore, it is reasonable to assume that electronic communication between anion and metal cation may only occur *via* the linker groups leading to a significant reduction of the charge at E^–^. This notion is in line with the results of the second order perturbation theory analysis of the NBO's of Na-**1**, Na-**2** and Na-**3**. It is further supported by a comparison of the X-ray data of Na-**1** with its protonated salt Na-**4**. Upon quenching the charge by protonation of Na-**1** the Si–C and Na–O_(Si)_ average distances increase from 1.79 to 1.87 and 2.37 to 2.40 Å, resp., while the Si–O average distances decrease from 1.70 to 1.66 Å.

### Basicity trends

Both, the computational and the experimental data clearly demonstrate the methanides M-**1** to be the strongest and the germanides M-**3** the weakest bases in the gas phase and in solution as well, regardless of the nature of the alkali metal counter cation.^20^ These results are in agreement with previous studies on experimental gas- and solution-phase acidities of CH_4_/SiH_4_ and (Me_3_Si)_3_CH/(Me_3_Si)_3_SiH revealing the silanes to be the stronger acids.^21,22^ Furthermore, the GPBs (and PAs) of the silanides M-**2** and germanides M-**3** correlate reasonably well with the NPA charges of E^–^; both numerical values increase progressively in the order Li < Na < K. This suggest that the more charge is localized at E^–^ the higher its affinity to bind to a proton, resulting in an increased gas-phase basicity of the respective zwitterion. It also appears to indicate that the basicity of these zwitterions is governed by the size of the cation. Such correlations, however, are not evident for the zwitterionic methanides M-**1**. The latter exhibit much lower NPA charges than the silyl and germyl derivatives M-**2**/**3**, despite their higher GPB's, PA's and experimental basicities. The lower NPA charges of M-**1**, however, are in agreement with their low-lying HOMOs and the results of the second order perturbation theory analysis of the NBO's.

**Fig. 7:**
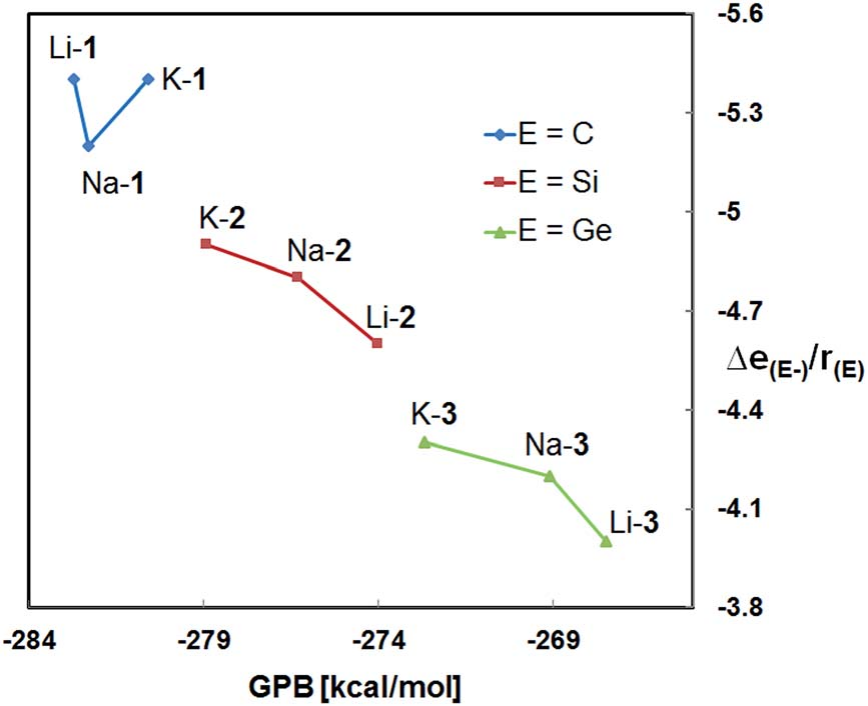
Plot of the calculated gas phase basicities (GPBs) *versus* the relative NPA charges of E^–^ divided by the covalent radius of E [Δ*e*_(E^–^)_/*r*_(E)_] of M-**1**–M-**3** (M = Li, Na, K).

These seemingly inconsistent observations raise the question why α-silyl stabilized carbanions, may they be zwitterionic or not, are stronger bases then their silicon and germanium counterparts?^23^ Recently, Apeloig and co-worker stated that silicon (despite being less electronegative than carbon) accommodates a negative charge more effectively and enables a better dispersion of charge than carbon.^22^ We agree and note specifically that carbon is significantly smaller (*r*_cov_ = 0.76 Å) than silicon (*r*_cov_ = 1.11 Å) and germanium (*r*_cov_ = 1.2 Å) resulting in a higher concentration of charge at the “naked” carbanion relative to those of the “naked” silyl and germyl anions (Fig. 7). Even though it may be an oversimplification to exclusively account size effects for the observed basicity trends, the structural changes occurring upon protonation to [M-**1** – H]^+^ (pyramidalization of the central carbon of the CSi_3_ framework) cannot be accounted for. According to the results of the DFT calculations, the energy differences between the conjugated acids [M-**1** – H]^+^ and [M-**2** – H]^+^ are smaller than those between the zwitterionic bases M-**1** and M-**2**.

**Scheme 7:**
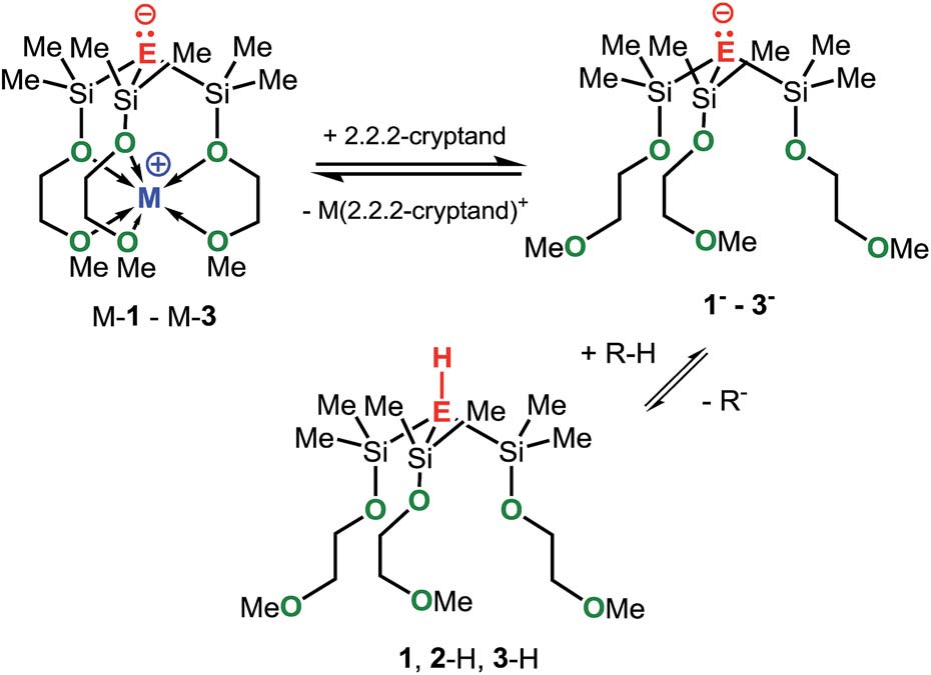
Interactions of M-**1**–M-**3** with 2.2.2-cryptand.

As mentioned before, the mixtures Na-**1**/2,2,2-cryptand, K-**2**/2,2,2-cryptand and K-**3**/2,2,2-cryptand showed significantly higher basicities than Na-**1**, K-**2** and K-**3**, respectively, (Table 1). We attribute the increase in basicity to the formation of the metal-free anions **1^–^**, **2^–^** and **3^–^**, respectively, which upon protonation generate the metal-free acids **1**, **2**-H and **3**-H, as illustrated in Scheme 7. In fact, the computational results are in line with the generation of **1^–^–3^–^**, as their calculated GPBs and PAs are significantly higher than those of the zwitterions M-**1**–M-**3**. Generally, cryptands are known to be excellent sequestering agents for group 1 and 2 metal cations and our recent studies with crown ethers have shown that Na-**2** and 18-crown-6, are in equilibrium with the metal free anion **2^–^** and the cationic crown ether complex [Na-18-crown-6]^+^.^6*c*^

In comparing the basicities of the zwitterions M-**1** and M-**2** with those of other stable alkali metal methanides and silanides (Table 7), the order in basicity for each series of methanides and silanides, regardless whether in solution or in the gas phase, was found to be as follows:
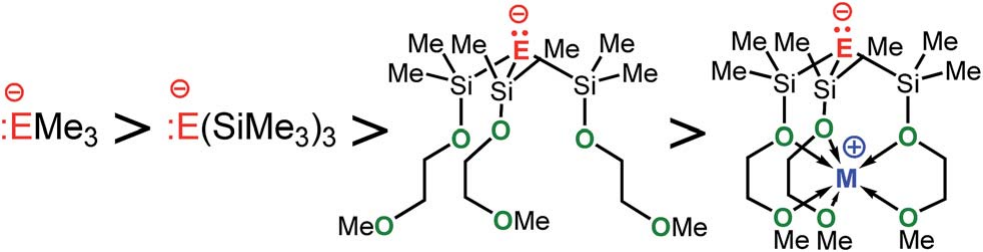


**Table 7:** Basicities of selected carb- and silyl anions

Base	p*K*_a_ ^*a*^	Base	GPB [kcal mol^–1^]
Me_3_CLi	>50^*b*^	Me_3_C^–^	–396.6^*h*^
(Me_3_Si)_3_CCs	36.8^*c*^ ^,^ ^*d*^	(Me_3_Si)_3_C^–^	–355.1
**1^–^** [Na-2,2,2-crypt]^+^	29.2^*c*^	**1^–^**	–348.8
K-**1**	24.5^*c*^	K-**1**	–280.6
Me_3_Si^–^	44.9^*e*^	Me_3_Si^–^	–376.2^*h*^
(Me_3_Si)_3_SiLi	29.4^*f*^	(Me_3_Si)_3_Si^–^	–349.3
**2^–^** [K-2,2,2-crypt]^+^	26 ± 1^*g*^	**2^–^**	–339.7
K-**2**	22.3^*c*^	K-**2**	–278.9

^*a*^The calculated p*K*_a_ values refer to the conjugated acids.

^*b*^Extrapolated.

^*c*^Measured in THF.

^*d*^Taken from ref. 24.

^*e*^Calculated for THF as solvent, ref. 25.

^*f*^Measured in diethyl ether, ref. 22.

^*g*^Measured in C_6_D_6_.

^*h*^Taken from ref. 26.

Apparently, silyl-substituted methanides and silanides such as (Me_3_Si)_3_C^–^ and (Me_3_Si)_3_Si^–^ benefit from hyperconjugative interactions leading to a significant decrease of their basicities as opposed to Me_3_SiLi and Me_3_CLi, strong bases that lack such interactions. That the metal free anions **1^–^** and **2^–^** are considerably weaker bases than (Me_3_Si)_3_C^–^ and (Me_3_Si)_3_Si^–^, respectively, can be explained by the presence of the electron-withdrawing alkoxide donor groups, which significantly reduce the electron density at E^–^ of the anions **1^–^** and **2^–^**. The further decrease in basicity that is seen with our zwitterions M-**1** and M-**2**, however, strongly suggests the metal cation to account for. As mentioned before, the metal must have significantly reduced the electron density of the “naked” anion E^–^, most likely by electronic cation–anion interactions *via* the linker groups. The experimental and computational results seem to favour electronic cation–anion interaction *via* the linker over “through space” as upon protonation only the structural parameter of the pendant donor arms significantly change, the metals NPA charges do not.

## Concluding remarks

The primary goal of this project was to comparatively study the electronic properties of a range of structurally rigid zwitterionic group 14 complexes of the alkali metals using experimental as well as theoretical methods. It was crucial to ensure that the various alkali metal ions are rigidly locked and insulated from the group 14 anions by appropriate donor bridges. Incorporating OCH_2_CH_2_OMe groups as pendant donors resulted in most cases in the formation of discrete zwitterions with the metal cations being in a rigid octahedral coordination environment. In fact, the X-ray data of M-**1**–M-**3** as well as a structural comparison with related non-zwitterionic species confirmed the alkali metal cations to be charge separated from their counter anions, C^–^, Si^–^ and Ge^–^, resp.

The methanides M-**1** are markedly different from their heavier homologues M-**2** and M-**3**, structurally as well as electronically. The latter zwitterions showed strong pyramidalization of the central anionic ^–^ESi_3_ framework and only a weak shortening of the average E–Si distances, whereas M-**1** exhibited significant shortening of the central ^–^C–Si bonds and a flattening of the central ^–^CSi_3_ framework primarily caused by negative hyperconjugative interactions. As a result, the HOMOs of M-**1**, which largely reside at the carbanion, are lower in energy, and also the calculated NPA charges of C^–^ are lower than those of Si^–^ in M-**2** and Ge^–^ in M-**3**. Clearly, that is consistent with the results of the second order perturbation theory analysis of the NBO's of Na-**1**, Na-**2** and Na-**3**, demonstrating the electron pair at the carbanion to be partially delocalized, while those of the corresponding silyl and germyl anions are largely localized. Despite partial delocalization of their electron pairs and lower NPA charges (Δ*e*_(E^–^)_), the methanides M-**1** are stronger bases than the silanides M-**2** and germanides M-**3**. This is attributed to the larger concentration of charge at the carbanion (charge to size ratio) of M-**1** relative to that of the anions in M-**2** and M-**3**. It was also found that the zwitterions M-**1**–M-**3** exhibit relatively low solution and gas-phase basicities as compared to their non-zwitterionic counterparts (Table 7), an observation that again is attributed to a reduced electron density of the “naked” anion E^–^, caused primarily by three factors: (1) negative hyperconjugative interaction, which decrease in the order M-**1** ≫ M-**2** > M-**3**; (2) the electron-withdrawing character of the pendant OCH_2_CH_2_OMe donor groups, and (3) electronic cation–anion interactions *via* the linker groups.

On a final note, the calculated GPBs of M-**1**–M-**3** are fairly similar to those of classical organosuperbases such as ^*t*^BuN=P[N=P(NMe_2_)_3_]_3_ (–288 kcal mol^–1^)^27^, [(Me_2_N)_2_C=N]_3_P=NBu^*t*^ (–279 kcal mol^–1^)^28^, HMPN (–270 kcal mol^–1^)^29^ and Verkade's base (–261 kcal mol^–1^)^30^, which have found use in organic synthesis and organocatalysis.^31,32^ Given the similar basicities, the ease of tuning basicity and the steric protection of the “naked” anion, E^–^, by proper choice of the metal cation and substituents, these types of zwitterions may offer new opportunities to be used in base and frustrated Lewis pair (FLP) catalysis as well as in the activation of small molecules. Studies along these lines are currently underway.
