# Supplementary material for: Electronic nature of zwitterionic alkali metal methanides, silanides and germanides – a combined experimental and computational approach
Source: Chem Sci. 2016 Oct 7;8(2):1316–28. doi: 10.1039/c6sc02390h (PMC5360169; doi:10.1039/c6sc02390h)
Supplement: Supplementary file 1 [file SC-008-C6SC02390H-s001.pdf]

Electronic Supplementary Material (ESI) for Chemical Science.
This journal is © The Royal Society of Chemistry 2016

Supporting Information

Electronic Nature of Zwitterionic Group 14 Anions of the Alkali Metals - A Combined Experimental and Computational Approach

Hui Li, Adelia J. A. Aquino, David B. Cordes, William L. Hase, Clemens Krempner*.

Texas Tech University, Department of Chemistry & Biochemistry, Box 41061, Lubbock, Texas, 79409-1061.

1. General Remarks S3

2. Synthetic Procedures S4

- **2.1. Synthesis of [C($SiMe_2OCH_2CH_2OMe)_3Li$] (Li-1)** S4
- **2.2. Synthesis of [C($SiMe_2OCH_2CH_2OMe)_3K$] (K-1)** S5
- **2.3. Synthesis of Ge($SiMe_2Cl)_4$** S5
- **2.4. Synthesis of Ge($SiMe_2OCH_2CH_2OMe)_4$ (3)** S6
- **2.5. Synthesis of [Ge($SiMe_2OCH_2CH_2OMe)_3Li$] (Li-3)** S7
- **2.6. Synthesis of [Ge($SiMe_2OCH_2CH_2OMe)_3Na$] (Na-3)** S8
- **2.7. Synthesis of [Ge($SiMe_2OCH_2CH_2OMe)_3K$] (K-3)** S9
- **2.8. Synthesis of [HC($SiMe_2OCH_2CH_2OMe)_3Na$][9-$SiMe_2Ph$-$C_{13}H_8$] (Na-4)** S10
- **2.9. Synthesis of [HC($SiMe_2OCH_2CH_2OMe)_3Li$][BH_4] (Li-5)** S11
- **2.10. Synthesis of [HC($SiMe_2OCH_2CH_2OMe)_3Na$][BPh_4] (Na-6)** S12

3. Estimation of the Basicities of M-1 – M-3 .... S13

4. X-ray crystallography S16

5. DFT Calculations S24

- **5.1. Results of calculations for M-1 and [M-1-H]$^+$ (M = Li, Na, K)** S25
- **5.2. Results of calculations for M-2 and [M-2-H]$^+$ (M = Li, Na, K)** S29
- **5.3. Results of calculations for M-3 and [M-3-H]$^+$ (M = Li, Na, K)** S32
- **5.4. Results of calculations for the metal free anions 1^-, 2^-, 3^-, 1, 2-H and 3-H** S35
- **5.5. Results of calculations for the metal free anions 7^-, 8^-, 9^-, 7-H, 8-H and 9-H** S44
- **5.6. B3-LYP/TZVP Cartesian coordinates [Å]** S46

6. References S128

1. General Remarks

All air- and moisture-sensitive manipulations were carried out using standard vacuum line, Schlenk or cannula techniques or in a Vacuum Atmospheres OMNI inert atmosphere drybox containing an atmosphere of purified nitrogen. THF, THF-D_8, toluene, diethyl ether and hexanes were distilled under nitrogen from alkali metals and stored over 4 Å molecular sieves prior to use. C_6D_6, THF-D_8, toluene-D_8, CD_2Cl_2 and $MeOCH_2CH_2OH$ were dried and stored over 4 Å molecular sieves prior to use. NEt_3 and CH_2Cl_2 were dried over CaH_2, distilled under nitrogen and stored over 4 Å molecular sieves. All deuterated solvents were purchased from Cambridge Isotope Labs. $Ph_2PCH_2PPh_2$ (Alfa Aesar), Bu^tLi (Acros), Bu^tOK (Alfa Aesar), Bu^tONa (TCI), Bu^tOLi (Aldrich), fluorene (Aldrich), $LiBH_4$ (Alfa Aesar), $NaBPh_4$ (Alfa Aesar) and 4,7,13,16,21,24-Hexaoxa-1,10-diazabicyclo[8.8.8]hexacosane [denoted as 2.2.2-cryptand] (Aldrich) were purchased from commercial sources and used without further purification. The compounds $Ge(SiMe_3)_4$ [1], $Si(SiMe_2Cl)_4$ [2], $Si(SiMe_2OCH_2CH_2OMe)_4$ (**2**) [3], $Si(SiMe_2OCH_2CH_2OMe)_3Li$ (Li-**2**) [3], $Si(SiMe_2OCH_2CH_2OMe)_3Li$ (Na-**2**) [3], $Si(SiMe_2OCH_2CH_2OMe)_3Li$ (K-**2**), [3], $HC(SiMe_3)_3$ [4], $HC(SiMe_2Cl)_3$ [4], $HC(SiMe_2OCH_2CH_2OMe)_3$ (**1**) [4], $C(SiMe_2OCH_2CH_2OMe)_3Na$ (Na-**1**) [4], Na-benzyl [4], K-benzyl [5], 9-Bu^t-fluorene [4], 9-Pr^i-fluorene [4], 9-Bu^n-fluorene [4], 9-Ph-fluorene [6], 9-Me_3Si-fluorene [7], 9-$PhMe_2Si$-fluorene [8] and 9-Ph_3Si-fluorene [8] were synthesized according to the literature procedures. The ^1H, ^{23}Na, ^7Li, ^{13}C, ^{31}P and ^{29}Si NMR spectra were obtained from a Varian Unity Inova 500 and JOEL ECS 400. All measurements, unless noted otherwise, were carried out at 298 K and NMR chemical shifts were given in ppm. The ^{23}Na NMR spectra were referenced to a 0.1 M solution of NaCl in D_2O (δ = 0), ^7Li NMR spectra were referenced to a 0.1 M solution of LiCl in D_2O (δ = 0) and the ^{29}Si NMR spectra to TMS (δ = 0 ppm). ^{29}Si NMR spectra were obtained by using the INEPT pulse sequence. The ^1H-NMR spectra were referenced to the residual protonated solvent for ^1H and the ^{13}C NMR spectra were referenced to the deuterated solvent peaks. The following abbreviations were used to describe peak multiplicities in the reported NMR spectroscopic data: 's' for singlet, "d" for doublet, "t" for triplet, "quart.' for quartet, "sept" for septet, "m" for multiplet and "br" for broadened resonances. Elemental analyses were performed using a Perkin Elmer 2400 Series II CHNS/O Analyzer.

2. Synthetic Procedures

2.1. Synthesis of $C(Si(CH_3)_2OCH_2CH_2OCH_3)_3Li$ (Li-1)

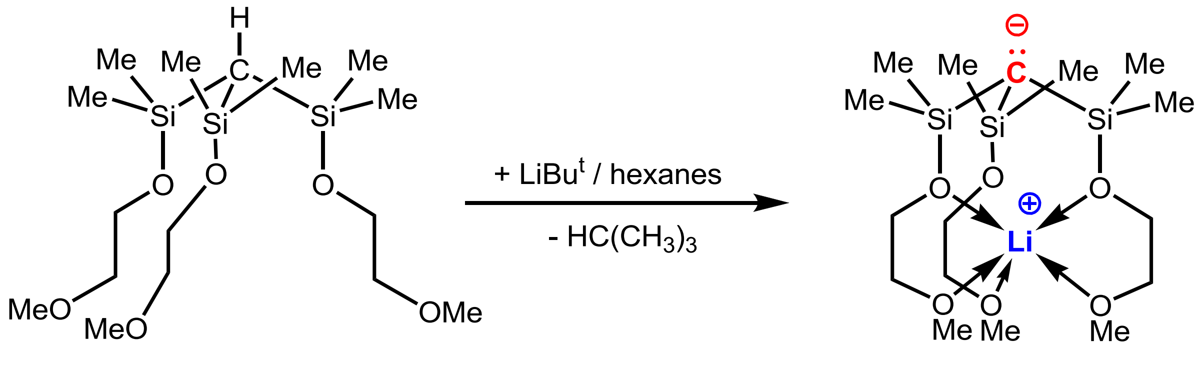

In a Glove box a 25 mL Schlenk flask equipped with a magnetic stir bar was charged with $HC(SiMe_2OCH_2CH_2OCH_3)_3$ (**1**) (120 mg, 0.29 mmol) and 1 mL of hexane. The resulting solution was pre-cooled in a freezer and then a hexane solution of $LiBu^t$ (0.2 mL, 1.5 M, 0.30 mmol) was added with stirring. After stirring the resulting solution for 2 hours at room temperature, the solvent was removed under vacuum to give raw Li-**1** as a colorless crystalline material. Recrystallization from hexanes gave an analytically pure sample of Li-**1** (92 mg, 76%). ^{1}H NMR (C_6D_6, 400 MHz): δ 0.53 (s, $Si(CH_3)_2$, 18 H), 2.95 (s, OCH_3, 9 H), 3.10 (t, $^3J_{H-H}$ = 4.8 Hz, CH_2O, 6 H), 3.51 (t, $^3J_{H-H}$ = 4.8 Hz, $SiOCH_2$, 6 H) ppm. ^{13}C{H} NMR (C_6D_6, 125.7 MHz): δ 4.3 ($Si(CH_3)_2$) , 60.2 (OCH_3), 61.9 (OCH_2), 74.4 ($\underline{C}H_2OCH_3$) ppm, signal from CSi_3 was not observed. ^{29}Si{H} NMR (C_6D_6, 99.3 MHz): δ 12.2 ($Si(CH_3)_2O$) ppm. ^{7}Li{H} NMR (C_6D_6, 194.2 MHz): δ -0.58 ppm. Anal. calc. for $C_{16}H_{39}O_6Si_3Li$ (418.67): C, 45.90; H, 9.39. Found: C, 46.20; H, 9.35%.

2.2. Synthesis of [C(SiMe$_2$OCH$_2$CH$_2$OMe)$_3$K] (K-1)

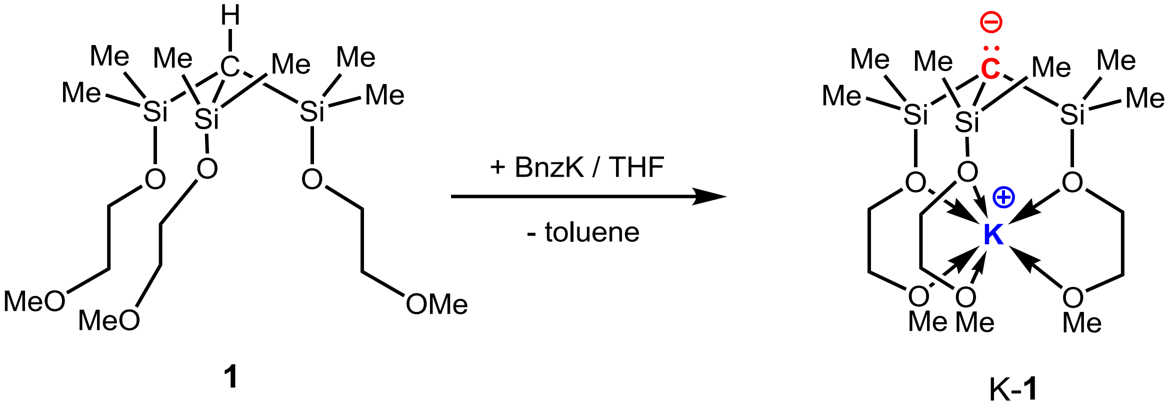

In a Glove box a 25 mL Schlenk flask equipped with a magnetic stir bar was charged with benzyl potassium (30mg, 0.26 mmol), HC(Si(CH$_3$)$_2$OCH$_2$CH$_2$OCH$_3$)$_4$ (**1**) (100 mg, 0.24 mmol) and 1 mL of THF. After stirring the resulting solution for 3 hours, the solvent was removed under vacuum. The solid residue recrystallized from hexanes to give K-**1** (46 mg, 42%). ^{1}H NMR (C$_6$D$_6$, 300 MHz): δ 0.54 (s, Si(CH$_3$)$_2$, 18 H), 2.87 (s, OCH$_3$, 9 H), 3.04 (t, $^3J_{H-H}$ = 4.5 Hz, CH$_2$O, 6 H), 3.47 (t, $^3J_{H-H}$ = 4.5 Hz, SiOCH$_2$, 6 H) ppm. ^{13}C{H} NMR (C$_6$D$_6$, 125.7 MHz): δ 3.6 (Si(CH$_3$)$_2$), 57.3(OCH$_3$), 60.1 (OCH$_2$), 75.0 ($\underline{C}$H$_2$OCH$_3$) ppm, signal from CSi$_3$ was not observed. ^{29}Si{H} NMR (C$_6$D$_6$, 99.3 MHz): δ 8.7 (Si(CH$_3$)$_2$O) ppm. Anal. calc. for C$_{16}$H$_{39}$O$_6$Si$_3$K (450.83): C, 42.63; H, 8.72. Found: C, 42.79; H, 8.84%.

2.3. Synthesis of Ge(SiMe$_2$Cl)$_4$

A 100 mL three-neck round bottom flask equipped with a reflux condenser, addition funnel and large magnetic stir bar was charged with Ge(SiMe$_3$)$_4$ (3 g, 8.2 mmol), AlCl$_3$ (6 g, 45 mmol) and 20 mL of dry hexanes. After the vigorously stirred suspension was heated (ca. 55-60°C), acetyl chloride (3 mL) dissolved in 10 mL of dry hexanes was slowly added via addition funnel within an hour. After the resulting emulsion (two liquid phases) was vigorously stirred with heating (ca. 55-60°C) overnight, 100 mL hexanes were added and the mixture was cooled to room temperature.

Then the upper phase was carefully separated from the viscous lower phase by decantation via cannula and the lower phase was extracted twice with hexanes. The solvent and other volatiles of the combined extracts were removed under vacuum to leave a solid. Vacuum distillation of the semi-solid using a Kugelrohrofen gave 2.6 g (71 %) of the title compound as a crystalline material. ^{1}H NMR (C_6D_6, 400 MHz): δ 0.63 (s, $Si(CH_3)_2$, 24 H) ppm. $^{13}C\{H\}$ NMR (C_6D_6, 125.7 MHz): δ 7.1 ($Si(CH_3)_2$) ppm. $^{29}Si\{H\}$ NMR (C_6D_6, 99.3 MHz): δ 30.1 ($Si(CH_3)_2$) ppm. Anal. calc. for $C_{12}H_{36}Si_4Ge$ (366.11): C, 39.44; H, 9.93. Found: C, 39.28; H, 9.89%.

2.4. Synthesis of $Ge(SiMe_2OCH_2CH_2OMe)_4$ (3)

A 50 mL Schlenk flask equipped with a magnetic stir bar was charged with NEt_3 (10.8 ml, 5.72 mmol) $Ge(Si(CH_3)_2Cl)_4$ (600 mg, 1.34 mmol) and hexanes (30 mL) and cooled to 0-5°C. Upon adding dropwise $HOCH_2CH_2SCH_3$ (2 ml, 25.4 mmol) via syringe a white suspension immediately formed, which was allowed to stir overnight at room temperature (the progress of the reaction was monitored by ^{1}H-NMR spectroscopy). Then the suspension was centrifuged, the liquid phase decanted and the solid phase washed two times with ca. 5 mL of hexanes. The organic phases were combined and solvent and excess $HOCH_2CH_2OCH_3$ were removed under high vacuum (ca. 10^{-1} mbar). Then 5 mL of hexanes were added to the residue, the mixture was centrifuged, the liquid phase decanted and the solvent evaporated under vacuum. Distillation of the liquid residue using a Kugelrohrofen afforded 470 mg (58%) of **3** as a colorless liquid. ^{1}H NMR (C_6D_6, 400 MHz): δ 0.60 (s, $Si(CH_3)_2$, 24 H), 3.15 (s, OCH_3, 12 H), 3.39 (t, $^{3}J_{H\text{-}H}$ = 5.5 Hz, CH_2O, 8 H) 3.80 (t, $^{3}J_{H\text{-}H}$ = 5.5 Hz, $SiOCH_2$, 8 H) ppm. $^{13}C\{H\}$ NMR (C_6D_6, 125.7 MHz): δ 4.6 ($Si(CH_3)_2$), 58.6 (O CH_3), 63.0 (OCH_2), 74.3 ($\underline{C}H_2OCH_3$) ppm. $^{29}Si\{H\}$ NMR (C_6D_6, 99.3 MHz): δ 25.1 ($Si(CH_3)_2O$) ppm. Anal. calc. for $C_{20}H_{52}O_8Si_4Ge$ (605.56): C, 39.67; H, 8.65. Found: C, 39.26; H, 8.77%.

2.5. Synthesis of [Ge(SiMe$_2$OCH$_2$CH$_2$OMe)$_3$Li] (Li-3)

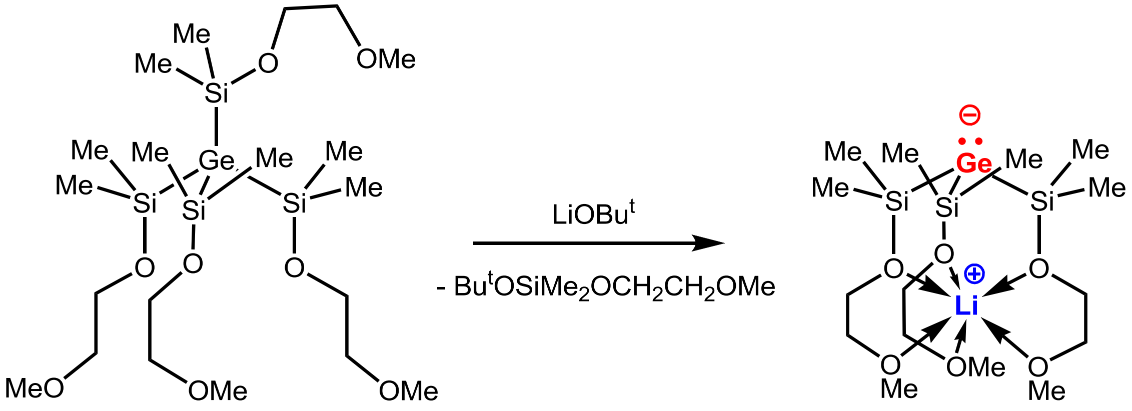

In a Glove box a 25 mL Schlenk flask equipped with a magnetic stir bar was charged with Bu^{t}OLi (14 mg, 0.17 mmol), Ge(Si(CH$_3$)$_2$OCH$_2$CH$_2$OCH$_3$)$_4$ (**3**) (100 mg, 0.17 mmol) and 1 mL of THF. After stirring the resulting solution for 1.5 hours, the solvent was removed under vacuum to give Li-**3** a yellow crystalline material nearly quantitatively (by ^{1}H and ^{13}C NMR). Recrystallization from toluene gave an analytically pure sample of Li-**3** (64 mg, 81%). ^{1}H NMR (C$_6$D$_6$, 400 MHz): δ 0.66 (s, Si(CH$_3$)$_2$, 18 H), 2.94 (s, OCH$_3$, 9 H), 2.99 (t, $^3J_{H\text{-}H}$ = 4.8 Hz, CH$_2$O, 6 H), 3.39 (t, $^3J_{H\text{-}H}$ = 4.8 Hz, SiOCH$_2$, 6 H) ppm. ^{13}C{H} NMR (C$_6$D$_6$, 125.7 MHz): δ 5.8 (Si(CH$_3$)$_2$), 58.5 (OCH$_3$), 61.1 (OCH$_2$), 73.6 ($\underline{C}$H$_2$OCH$_3$) ppm. ^{29}Si{H} NMR (C$_6$D$_6$, 99.3 MHz): δ 47.9 (Si(CH$_3$)$_2$(CH$_3$)$_2$O) ppm. ^{7}Li{H} NMR (C$_6$D$_6$, 194.2 MHz): δ -1.1 ppm. Anal. calc. for C$_{15}$H$_{39}$O$_6$Si$_3$GeLi (479.30): C, 37.59; H, 8.20. Found: C, 38.11; H, 8.09 %.

2.6. Synthesis of [Ge(SiMe$_2$OCH$_2$CH$_2$OMe)$_3$Na] (Na-3)

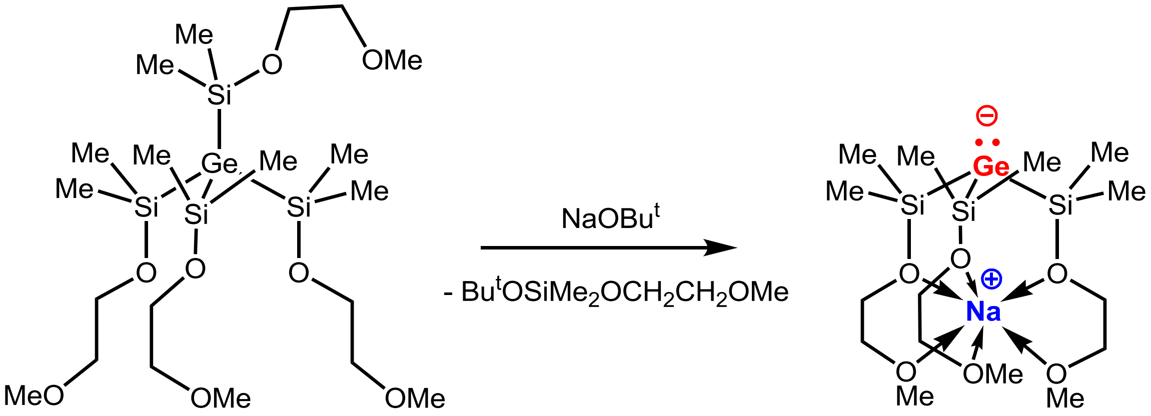

Na-**3**

In a Glove box a 25 mL Schlenk flask equipped with a magnetic stir bar was charged with Bu^{t}ONa (17 mg, 0.18 mmol), Ge(Si(CH$_3$)$_2$OCH$_2$CH$_2$OCH$_3$)$_4$ (**3**) (100 mg, 0.17 mmol) and 1 mL of THF. After stirring the resulting solution for 1.5 hours, the solvent was removed under vacuum to give raw Na-**3** as a red crystalline material nearly quantitatively (by ^{1}H and ^{13}C NMR). Recrystallization from hexanes gave an analytically pure sample of Na-**3** (68 mg, 83%). ^{1}H NMR (C$_6$D$_6$, 400 MHz): δ 0.70 (s, Si(CH$_3$)$_2$, 18 H), 2.88 (s, OCH$_3$, 9 H), 2.93 (t, $^3J_{H\text{-}H}$ = 4.4 Hz, CH$_2$O, 6 H), 3.38 (t, $^3J_{H\text{-}H}$ = 4.4 Hz, SiOCH$_2$, 6 H) ppm. ^{13}C{H} NMR (C$_6$D$_6$, 125.7 MHz): δ 5.7 (Si(CH$_3$)$_2$), 58.1 (OCH$_3$), 60.8(OCH$_2$), 74.4 (C̲H$_2$OCH$_3$) ppm. ^{29}Si{H} NMR (C$_6$D$_6$, 99.3 MHz): δ 45.4 (Si(CH$_3$)$_2$O) ppm. ^{23}Na{H} NMR (C$_6$D$_6$, 132.2 MHz): δ 0.4 ppm. Anal. calc. for C$_{15}$H$_{39}$O$_6$Si$_3$GeNa (495.39): C, 36.37; H, 7.94. Found: C, 36.59; H, 7.99%.

2.7. Synthesis of [Ge(SiMe$_2$OCH$_2$CH$_2$OMe)$_3$K] (K-3)

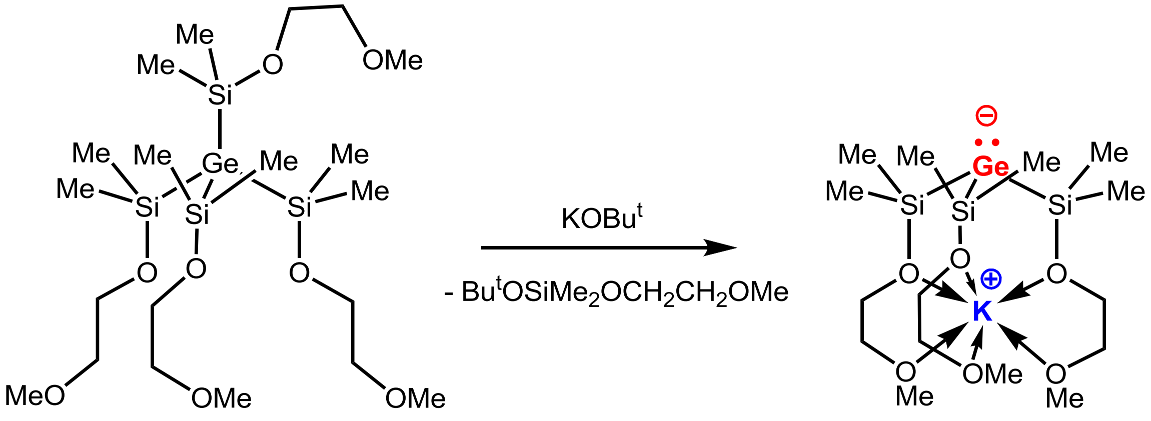

In a Glove box a 25 mL Schlenk flask equipped with a magnetic stir bar was charged with Bu^{t}OK (20 mg, 0.18 mmol), Ge(Si(CH$_3$)$_2$OCH$_2$CH$_2$OCH$_3$)$_4$ (**3**) (100 mg, 0.17 mmol) and 1 mL of THF. After stirring the resulting solution for 1.5 hours, the solvent was removed under vacuum to give K-**3** as a brown crystalline material nearly quantitatively (by ^{1}H and ^{13}C NMR). Recrystallization from hexanes gave an analytically pure sample of K-**3** (67 mg, 79%). ^{1}H NMR (C$_6$D$_6$, 400 MHz): δ 0.73 (s, Si(CH$_3$)$_2$, 18 H), 2.86 (s, OCH$_3$, 9 H), 2.97 (t, $^3J_{H-H}$ = 4.4 Hz, CH$_2$O, 6 H), 3.44 (t, $^3J_{H-H}$ = 4.4 Hz, SiOCH$_2$, 6 H) ppm. ^{13}C{H} NMR (C$_6$D$_6$, 125.7 MHz): δ 5.7 (Si(CH$_3$)$_2$), 57.6 (OCH$_3$), 60.9 (OCH$_2$), 74.9 (<u>C</u>H$_2$OCH$_3$) ppm. ^{29}Si{H} NMR (C$_6$D$_6$, 99.3 MHz): δ 41.1 (Si(CH$_3$)$_2$) ppm. Anal. calc. for C$_{15}$H$_{39}$O$_6$Si$_3$GeK (511.46): C, 35.22; H, 7.69. Found: C, 35.67; H, 8.08 %.

2.8. Synthesis of [HC($SiMe_2OCH_2CH_2OMe$)$_3$Na][9-$SiMe_2Ph$-$C_{13}H_8$] (Na-4)

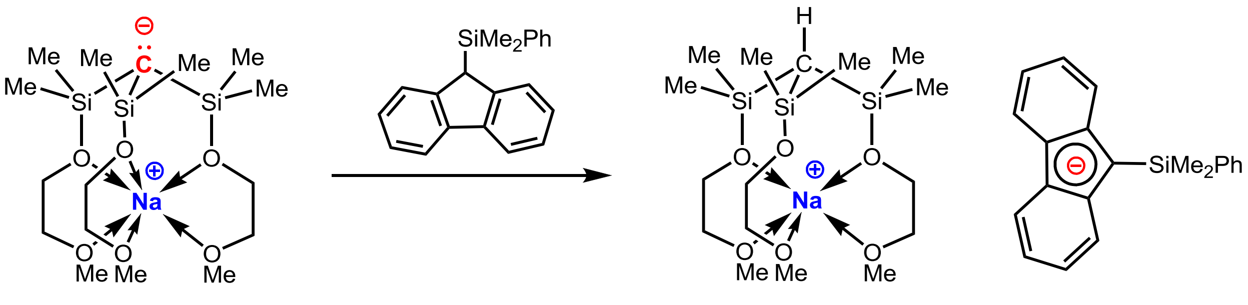

Na-**1** Na-**4**

In a Glove box a 25 mL Schlenk flask equipped with a magnetic stir bar was charged with 9-$SiMe_2Ph$-fluorene (55 mg, 0.18 mmol), Na-**1** (80 mg, 0.18 mmol) and 1 mL of THF. After stirring the resulting solution for 3 hours, the solvent was removed under vacuum and the solid residue was washed twice with hexanes to give 123 mg of Na-**4** as a pale yellow powder (91%). The compound is thermally labile and slowly decomposes upon storage in the box. ^{1}H NMR (THF-D_8, 400 MHz): δ -0.25 (b, $HCSi_3$, 1 H) 0.26 (s, $Si(CH_3)_2O$, 18 H), 0.90 (s, $Si(CH_3)_2Ph$, 6 H) 3.15 (m, CH_2O and OCH_3, 15 H), 3.43 (t, $^{3}J_{H-H}$ = 4.6 Hz, $SiOCH_2$, 6 H), 6.85, 7.11, 7.29, 7.38, 7.81, 7.82, 8.22 (arom. H, 13 H) ppm. $^{13}C\{H\}$ NMR (THF-D_8, 125.7 MHz): δ 1.6 ($Si(CH_3)_2O$), 2.2 ($Si(CH_3)_2Ph$), 10.1 (CSi_3), 58.9 (OCH_3), 62.1 (OCH_2), 74.2 ($\underline{C}H_2OCH_3$), 81.1 (C^- from $CSi(CH_3)_2Ph$) 111.1, 119.2, 119.4, 120.8, 126.8, 127.8, 127.9, 135.3, 144.8, 146.2 (arom. C) ppm. $^{29}Si\{H\}$ NMR (THF-D_8, 99.3 MHz): δ 17.5 ($Si(CH_3)_2O$), -16.4 ($Si(CH_3)_2Ph$) ppm. $^{23}Na\{H\}$ NMR (THF-D_8, 132.2 MHz): δ -9.0 ppm.

2.9. Synthesis of [HC(SiMe$_2$OCH$_2$CH$_2$OMe)$_3$Li][BH$_4$] (Li-5)

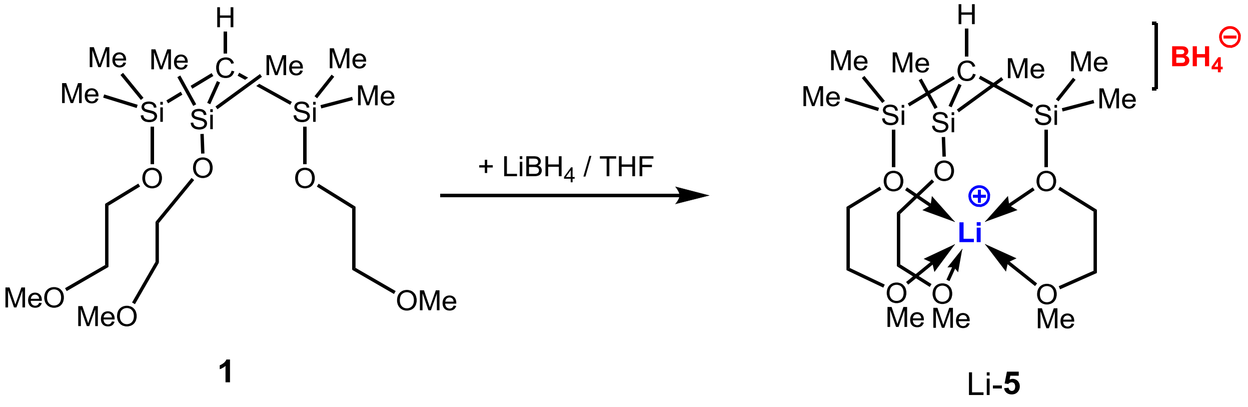

In a Glove box a 25 mL Schlenk flask equipped with a magnetic stir bar was charged with LiBH$_4$ (5 mg, 0.23 mmol), HC(Si(CH$_3$)$_2$OCH$_2$CH$_2$OCH$_3$)$_3$ (**1**) (100 mg, 0.24 mmol) and 1 mL of THF. The resulting suspension was stirred until it became a clear solution (approximately after a week). Then, the solvent was removed under vacuum to leave a crystalline residue, which was washed twice with hexanes and dried under vacuum. Yield (90 mg, 86%). ^{1}H NMR (C$_6$D$_6$, 400 MHz): δ 0.31 (b, HCSi$_3$, 1 H) 0.56 (quart., $^{1}J_{H\text{-}B}$ = 81.2 Hz, BH$_4$, 4 H), 1.16 (s, SiMe$_2$O, 18 H), 3.10 (t, $^{3}J_{H\text{-}H}$ = 4.6 Hz, CH$_2$O, 6 H), 3.19 (s, OCH$_3$, 9 H), 3.35 (t, $^{3}J_{H\text{-}H}$ = 4.6 Hz, SiOCH$_2$, 6 H), ppm. ^{13}C{H} NMR (C$_6$D$_6$, 100.6 MHz): δ 0.6 (SiMe$_2$O), 9.4 (CSi$_3$), 58.6 (OMe), 60.9 (OCH$_2$), 72.5 (C̲H$_2$OMe) ppm. ^{29}Si{H} NMR (C$_6$D$_6$, 79.5 MHz): δ 18.6 ppm. ^{7}Li{H} NMR (C$_6$D$_6$, 155.5 MHz): δ 2.5 ppm.^{11}B{H} NMR (C$_6$D$_6$, 128.3 MHz): -40.2 (quintet, $^{1}J_{H\text{-}B}$ = 81.2 Hz) ppm. Anal. calc. for C$_{16}$H$_{44}$O$_6$Si$_3$BLi (434.525): C, 44.23; H, 10.21. Found: C, 43.97; H, 10.08 %.

2.10. Synthesis of $[HC(SiMe_2OCH_2CH_2OMe)_3Na][BPh_4]$ (Na-6)

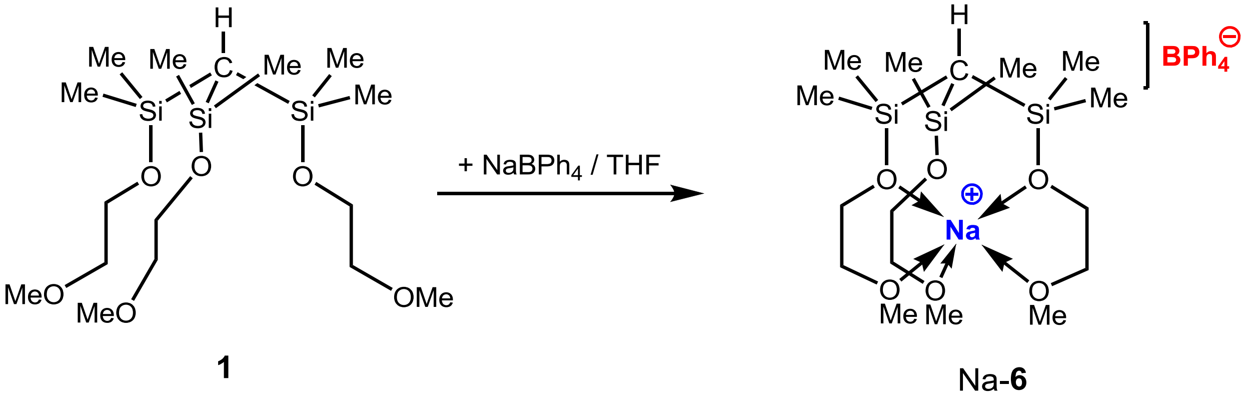

In a Glove box a 25 mL Schlenk flask equipped with a magnetic stir bar was charged with $NaBPh_4$ (50 mg, 0.15 mmol), $HC(Si(CH_3)_2OCH_2CH_2OCH_3)_3$ (**1**) (60 mg, 0.15 mmol) and 2 mL of CH_2Cl_2. The resulting suspension was stirred overnight until it became a clear solution. Then, the solvent was removed under vacuum to leave crystalline residue, which was washed twice with small amounts of benzene and dried under vacuum. Yield (98 mg, 89%). ^{1}H NMR (C_6D_6, 400 MHz): δ -1.38 (b, $HCSi_3$, 1 H) -0.11 (s, $SiMe_2O$, 18 H), 2.69 (t, $^{3}J_{H\text{-}H}$ = 4.6 Hz, CH_2O, 6 H), 2.78 (s, OCH_3, 9 H), 2.89 (t, $^{3}J_{H\text{-}H}$ = 4.6 Hz, $SiOCH_2$, 6 H), 7.27 (t, *p*-H 4 H), 7.44 (t, *m*-H, 8 H), 8.16 (b, *o*-H, 8 H) ppm. ^{1}H NMR ($CDCl_3$, 400 MHz): δ -0.72 (b, $HCSi_3$, 1 H) 0.22 (s, $SiMe_2O$, 18 H), 3.29 (s, OCH_3, 9 H), 3.33 (t, $^{3}J_{H\text{-}H}$ = 4.6 Hz, CH_2O, 6 H), 3.55 (t, $^{3}J_{H\text{-}H}$ = 4.6 Hz, $SiOCH_2$, 6 H), 6.89 (m, *p*-H, 4 H), 7.04 (m, *m*-H, 8 H), 7.40 (b, *o*-H, 8 H) ppm. $^{13}C\{H\}$ NMR ($CDCl_3$, 100.6 MHz): δ 1.8 ($SiMe_2O$), 7.9 (CSi_3), 51.2 (OMe), 61.4 (OCH_2), 73.1 ($\underline{C}H_2OMe$), 121.6, 125.5, 136.4, 163.6, 164.1, 164.6, 165.1 (aromatic C) ppm. $^{29}Si\{H\}$ NMR ($CDCl_3$, 79.5 MHz): δ 21.2 ($SiMe_2O$), ppm. $^{23}Na\{H\}$ NMR ($CDCl_3$, 105.8 MHz): δ 0.1 ppm. $^{11}B\{H\}$ NMR ($CDCl_3$, 128.3 MHz): δ -7.5 ppm. Anal. calc. for $C_{40}H_{60}O_6Si_3BNa$ (754.958): C, 63.64; H, 8.01. Found: C, 63.61; H, 7.96 %.

3. Estimation of the Basicities of M-1 – M-3

General procedure: In the glove box, a J-Young NMR tube was charged with one equiv. (ca. 0.02 mmol) of the respective zwitterion M-**1** , M-**2** or M-**3**, one equivalent (ca. 0.02 mmol) of the hydrocarbon acid (Ind-H) and ca. 0.4 mL of solvent (THF-D_8 or C_6D_6). For some experiments (see Tables S1 and S2) one equivalent of 2.2.2-cryptand was added as well. ^{1}H NMR spectra of the resulting solutions were taken immediately and after ca. 3 hours if not mentioned otherwise. The ratios product to starting material were estimated from integrating the respective signals in ^{1}H-NMR spectrum. The relative pK_a-values were calculated according to the following equation:

$$\mathrm{pKa(acid)} = \mathrm{pKa(IndH)} + \lg\frac{[\mathrm{acid}][\mathrm{Ind}^-]}{[\mathrm{base}][\mathrm{IndH}]}$$

M-**1** + IndH ⇄ [M-**1**-H]$^+$ + Ind$^-$ (eq. 1)

M-**2** + IndH ⇄ [M-**2**-H]$^+$ + Ind$^-$ (eq. 2)

M-**3** + IndH ⇄ [M-**3**-H]$^+$ + Ind$^-$ (eq. 3)

[Na(2.2.2-cryptand)]$^+$ + **1**$^-$ + IndH ⇄ [Na(2.2.2-cryptand)]$^+$ + **1** + Ind$^-$ (eq. 4)

[K(2.2.2-cryptand)]$^+$ + **2**$^-$ + IndH ⇄ [K(2.2.2-cryptand)]$^+$ + **2**-H + Ind$^-$ (eq. 5)

[K(2.2.2-cryptand)]$^+$ + **3**$^-$ + IndH ⇄ [K(2.2.2-cryptand)]$^+$ + **3**-H + Ind$^-$ (eq. 6)

Table S1. Estimated pK_a values of M-**1** - M-**3** (M = Li, Na, K) derived from acid-base measurements by ^{1}H-NMR spectroscopy in THF-D_8.

Acid [IndH]	pk$_a$ [DMSO] [a]	Base [M-**1** – M-**3**]	conv. [%]	pk$_a$ [calc.[[a]
$Ph_3PCH_2PPh_3$	29.9	Na-**1**/(2.2.2-cryptand)	30	29.2
9-Bu^t-fluorene	24.4	K-**1**	10	-
9-Pr^i-fluorene	23.2	K-**1**	82	24.5
9-Bu^t-fluorene	24.4	Na-**1**	0	-
9-Pr^i-fluorene	23.2	Na-**1**	62	23.6
9-Bu^n-fluorene	22.2	Na-**1**	86	23.8
9-Bu^t-fluorene	24.4	Li-**1**	0	-
9-Pr^i-fluorene	23.2	Li-**1**	62	23.6
9-Bu^n-fluorene	22.2	K-**2**	53	22.3
9-Me_3Si-fluorene	21.5	K-**2**	70	22.2
9-Ph_3Si-fluorene	18.6	Li-**2**	80	19.8
9-Ph-fluorene	17.9	Li-**2**	100	-
9-Ph_3Si-fluorene	18.6	Na-**2**	56	18.8
9-Ph_3Si-fluorene	18.6	K-**3**	33	18.0
9-Ph-fluorene	17.9	Li-**3**	9	“15.9”
9-Ph-fluorene	17.9	Na-**3**	0	-

[a] It is customary among chemists to use the term "pK_a of base" with the actual meaning of the pK_a of the conjugate acid of the respective base.

Table S2. Estimated pK_a values of M-**1** - M-**3** (M = Li, Na, K) derived from acid-base measurements by ^{1}H-NMR spectroscopy in C_6D_6.

Acid [Ind-H]	pK_a [DMSO]	Base [M-**1** – M-3]	Conv. [%]	pK_a [calc.] [a]
$Ph_3PCH_2PPh_3$	29.9	K-**2**/(2.2.2-cryptand)	0	-
9-Bu^t-fluorene	24.2	K-**2**/(2.2.2-cryptand)	100	-
9-Pr^i-fluorene	23.2	K-**3**/(2.2.2-cryptand)	42	22.9
9-Pr^i-fluorene	23.2	Li-**1**	45	23.0
9-Bu^n-fluorene	22.2	Li-**1**	69	22.9
9-Bu^t-fluorene	24.2	Na-**1**	0	**-**
9-Pr^i-fluorene	23.2	Na-**1**	0	**-**
9-Bu^n-fluorene	22.2	Na-**1**	59	22.5
9-Bu^n-fluorene	22.2	K-**1**	50	22.2
9-Ph_3Si-fluorene	18.6	K-**2**	53	18.7
9-Ph_3Si-fluorene	18.6	Li-**2**	33	18.0
9-Ph_3Si-fluorene	18.6	Na-**2**	0	-
9-Ph-fluorene	17.9	Na-**2**	10	“16.0”
9-Ph-fluorene	17.9	Li-**3**	0	-
9-Ph-fluorene	17.9	Na-**3**	0	-
9-Ph-fluorene	17.9	K-**3**	0	-

[a] It is customary among chemists to use the term "pK_a of base" with the actual meaning of the pK_a of the conjugate acid of the respective base.

4. X-ray crystallography

X-ray diffraction data for all compounds were obtained on a Bruker Smart Apex II CCD diffractometer, using graphite-monochromated Mo-Kα radiation (**λ** = 0.71073 Å) at low temperature (150 K for Li-**1**, Na-**3**, and Na-**4**; 100 K for K-**1** and K-**3**). Intensity data were collected using ω-steps accumulating area detector images spanning at least a hemisphere of reciprocal space. All the data were corrected for Lorentz polarization effects. A multi-scan absorption correction was applied using SADABS [9]. Structures were solved by direct methods and refined by full-matrix least-squares against F^2 (SHELXTL) [10]. All hydrogen atoms were assigned riding isotropic displacement parameters and constrained to idealized geometries.

The flack parameter in Li-**1** was found to be meaningless, due to weak anomalous scattering by the crystals. In K-**1**, as refined as a pseudo-merohedral twin with a twin law of -1 0 0 0 -1 0 0 0 1 and a refined twin fraction of 0.44. The RIGU command was also used as a general constraint to the ellipsoids of the structure.

Crystallographic data for the five structures are listed in Tables S3 and S4. CCDC-1000501 (Li-**1**), CCDC-1013011 (K-**1**), CCDC–1000506 (Na-**3**), 1000507 (K-**3**) and CCDC-926164 (Na-**4**) contain the supplementary crystallographic data for this paper. These data can be obtained free of charge from the Cambridge Crystallographic Data Centre via http://www.ccdc.cam.ac.uk/data_request/cif, or from the Cambridge Crystallographic Data Centre, 12 Union Road, Cambridge CB2 1EZ, UK; fax: (+44) 1223-336-033; e-mail: deposit@ccdc.cam.ac.uk.

Table S3. Crystallographic data for complexes Li-**1** and K-**1**.

	Li-**1**	K-**1**
Formula	$C_{16}H_{39}LiO_6Si_3$	$C_{16}H_{39}KO_6Si_3$
Molecular weight	418.68	450.84
Crystal size (mm^3)	0.27 × 0.20 × 0.08	0.48 × 0.39 × 0.15
Crystal appearance	Colorless chunk	Colorless block
Crystal system	Monoclinic	Monoclinic
Space group	*Cc* (No. 9)	$P2_1/n$ (No. 14)
a (Å)	9.531(5)	8.698(6)
b (Å)	17.403(8)	18.417(12)
c (Å)	15.039(7)	16.289(11)
α		
β (°)	90.873(6)	90.200(10)
γ		
Volume (Å^3)	2494(2)	2609(3)
Z	4	4
Temperature (K)	150(2)	100(2)
μ (mm^{-1})	0.214	0.365
θ range (°)	2.34 to 27.78	1.106 to 25.816
Reflections collected	14494	26816
Unique reflections (R_{int})	5737 (0.1480)	4942 (0.1401)
R1 [$I>2\sigma(I)$]	0.0762	0.0699
wR2 indices (all data)	0.1585	0.1863
GOF	0.949	0.936
Data/restraints/parameters	5737 / 2 / 244	4942 / 369 / 237

Table S4. Crystallographic data for complexes Na-**3** and K-**3** and Na-**4**

	Na-**3**	K-**3**	Na-**4**
Formula	$C_{15}H_{39}GeNaO_6Si_3$	$C_{15}H_{39}GeKO_6Si_3$	$C_{37}H_{59}NaO_6Si_4$
Molecular weight	495.31	511.42	735.19
Crystal size (mm^3)	0.36 × 0.21 × 0.15	0.34 × 0.28 × 0.25	0.37 × 0.21 × 0.07
Crystal appearance	Yellow prism	Yellow chunk	Orange prism
Crystal system	Monoclinic	Monoclinic	Monoclinic
Space group	$P2_1/c$ (No. 14)	$P2_1/n$ (No. 14)	$C2/c$ (No. 15)
a (Å)	15.729(2)	8.141(2)	23.272(4)
b (Å)	9.9209(13)	19.245(6)	9.7485(17)
c (Å)	17.212(2)	16.727(5)	37.173(6)
Α			
β (°)	90.3515(14)	90.528(4)	94.149(2)
Γ			
Volume (Å^3)	2685.8(6)	2620.5(13)	8411(3)
Z	4	4	8
Temperature (K)	150(2)	100(2)	150(2)
μ (mm^{-1})	1.313	1.489	0.191
θ range (°)	2.37 to 27.88	1.06 to 27.91	1.10 to 27.92
Reflections collected	31353	27981	49332
Unique reflections (R_{int})	6374 (0.0292)	6104 (0.0539)	9999 (0.0613)
R1 [$I>2\sigma(I)$]	0.0266	0.0960	0.0779
wR2 indices (all data)	0.0682	0.2175	0.1178
GOF	1.035	1.279	1.035
Data/restraints/parameters	6374 / 0 / 244	6104 / 0 / 245	9999 / 0 / 444

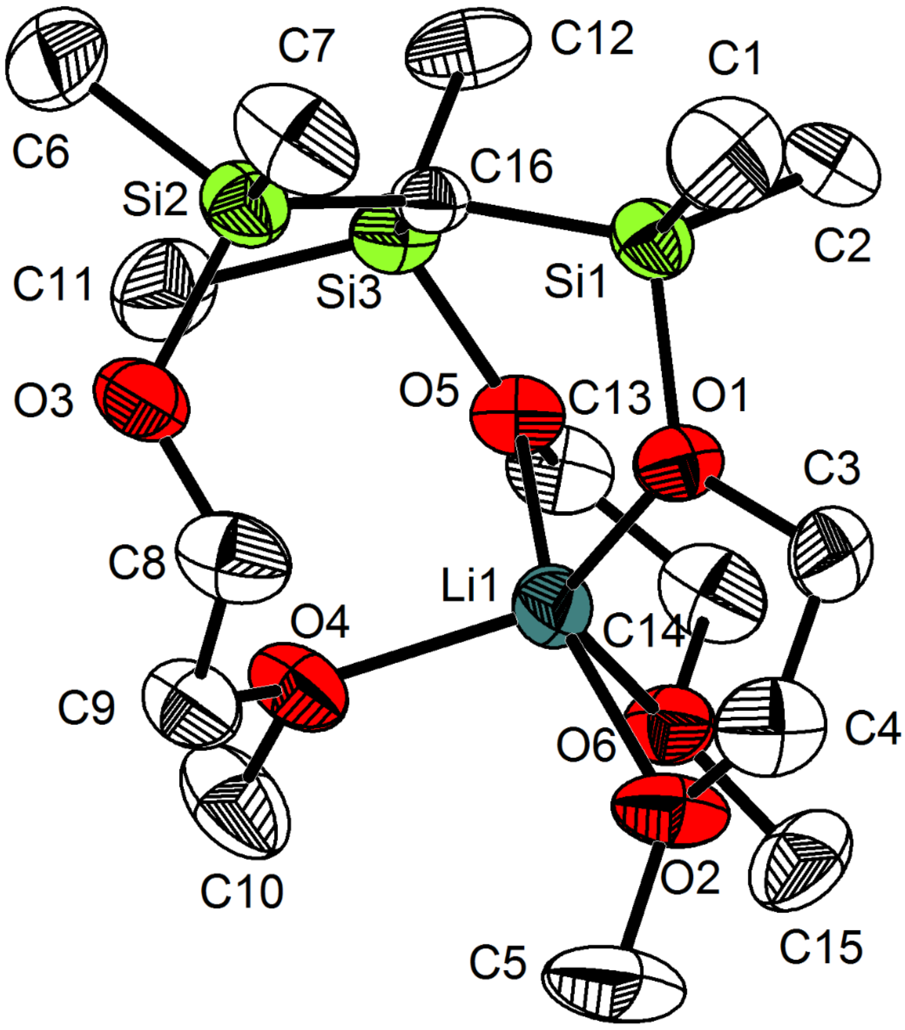

Figure S1. Solid-state structure of Li-**1**.

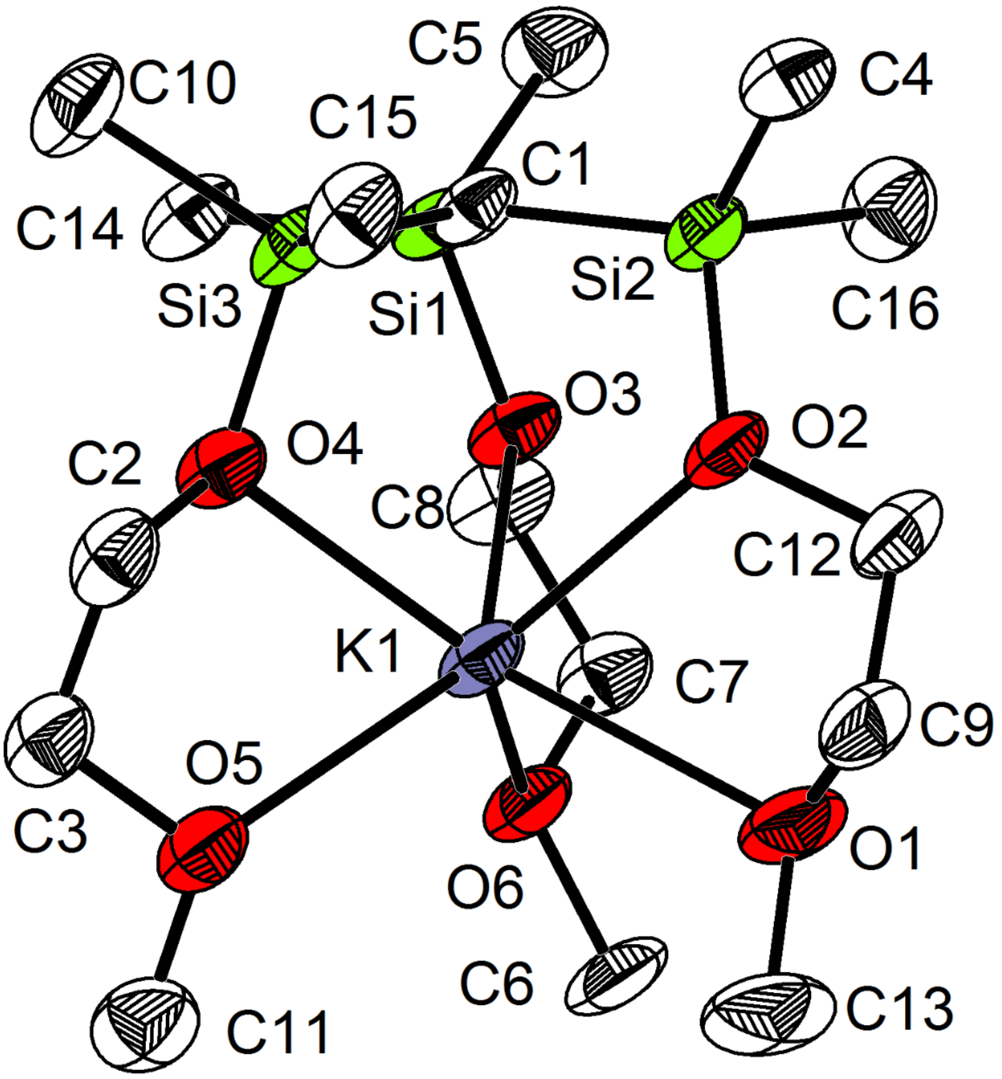

Figure S2. Solid-state structure of K-**1**.

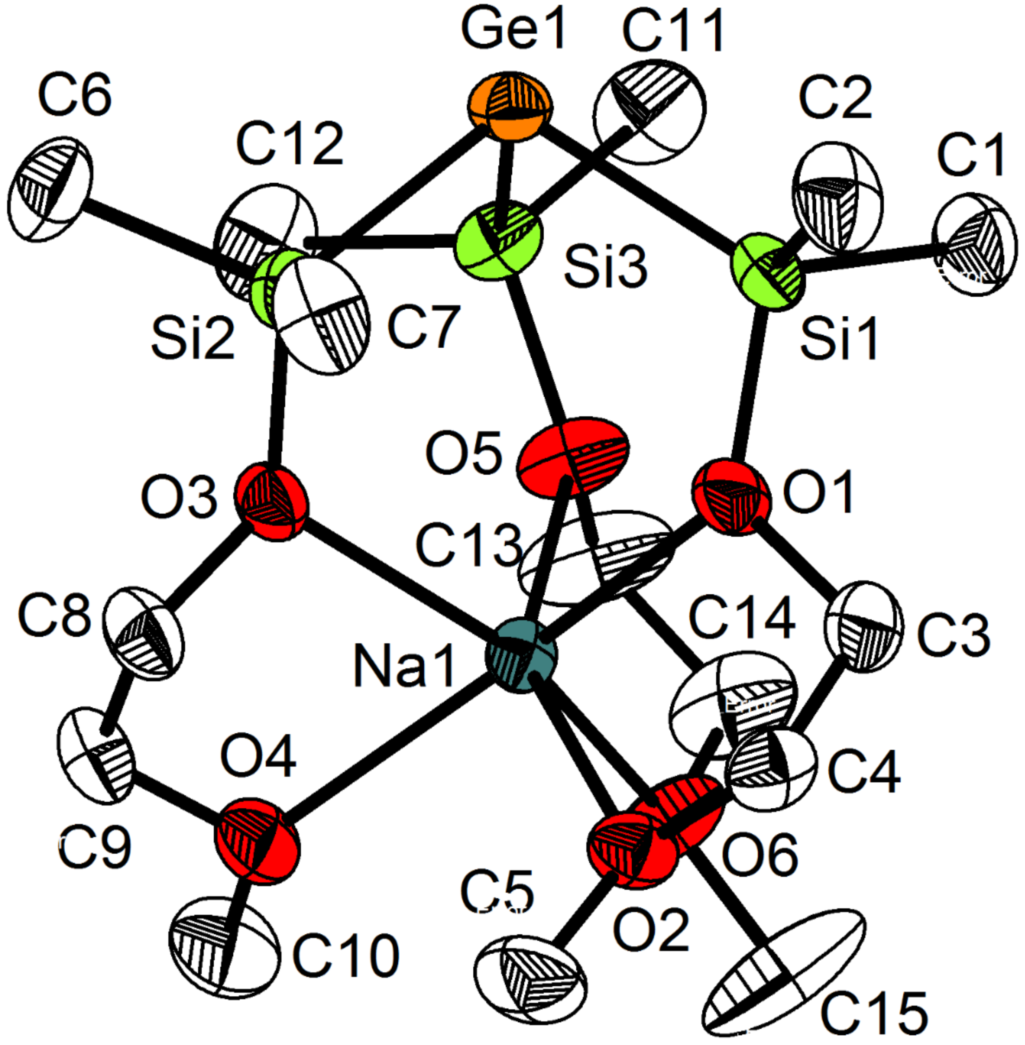

Figure S3. Solid-state structure of Na-**3**.

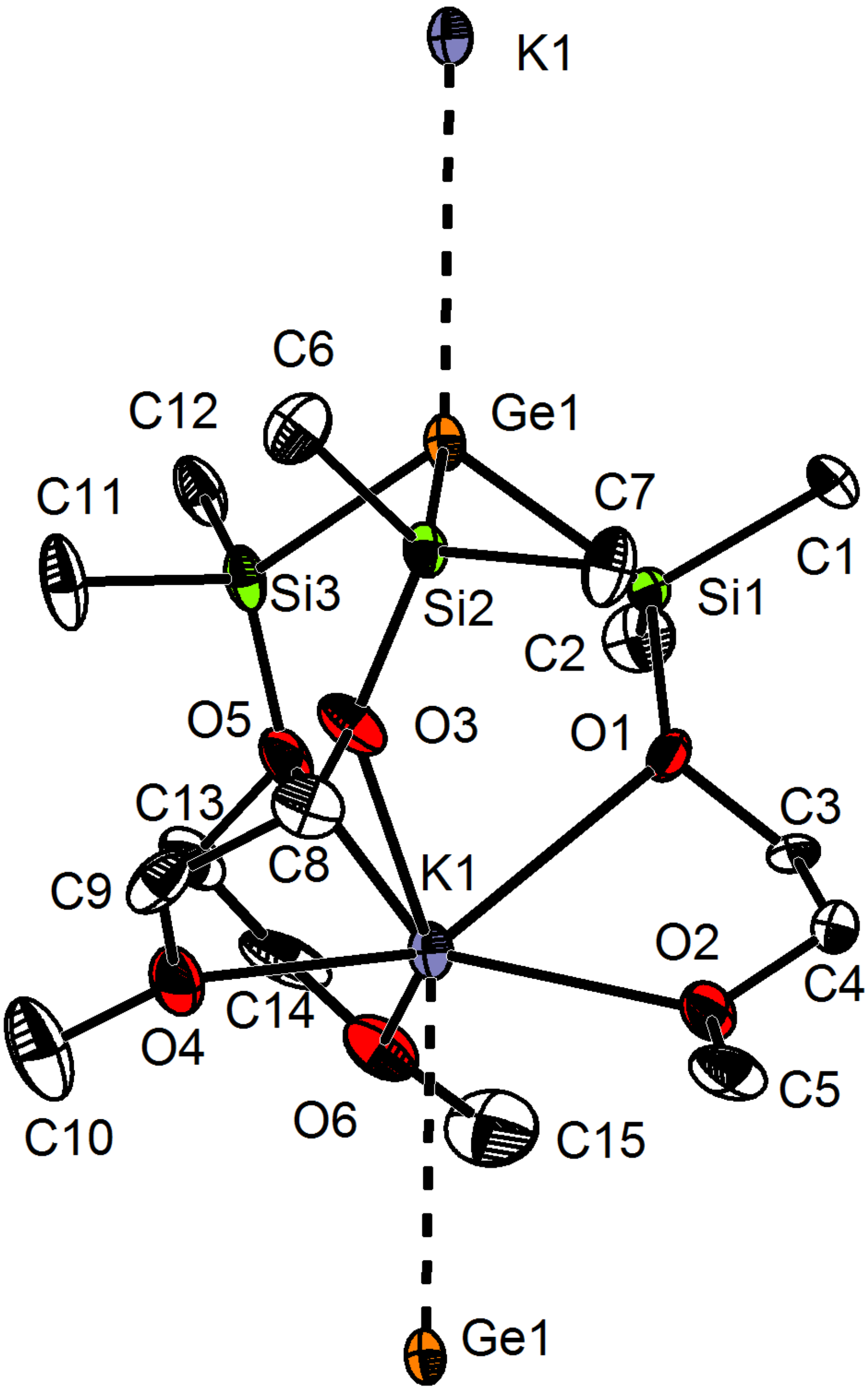

Figure S4. Solid-state structure of K-**3**.

Table S5. Selected average distances [Å] and angles [°] of M-**1** –M-**3**.

M-**1** [b]	Li-**1**	Na-**1**	K-**1**	M-**2** [b]	Li-**2**	Na-**2**	K-**2**	M-**3** [b]	Na-**3**	K-**3**
CN [a]	5	6	6	CN	6	6	7	CN	6	7
C-Si	1.78	1.79	1.80	Si-Si	2.32	2.32	2.32	Ge-Si	2.37	2.37
Si-O	1.71	1.70	1.70	Si-O	1.69	1.68	1.68	Si-O	1.68	1.67
O-M	2.01	2.37	2.69	O-M	2.08	2.32	2.74	O-M	2.32	2.75
C···M	3.30	3.23	3.32	Si···M	3.92	4.00	4.50	Ge···M	4.05	4.53
Si-C-Si	119	116	119	Si-Si-Si	96	99	100	Si-Ge-Si	97	98
C-Si-O	106	107	108	Si-Si-O	113	113	113	Ge-Si-O	113	114
Si-O-M	118	110	107	Si-O-M	126	122	125	Si-O-M	123	125

[a] CN = coordination number; [b] M = metal.

5. DFT Calculations

The calculations were performed at density functional theory level through the functional B3-LYP [11] and the TZVP [12, 13] basis set. Geometry optimizations were performed for all systems studied in this work. Gas-phase basicity (GB) and proton affinity (PA) computations were carried out at the same level of theory by means of harmonic vibrational frequency calculations to get corresponding enthalpies and Gibbs free energies. The atomic charges were calculated using natural population analysis [14]. All calculations were performed using the Turbomole [15] suite program. The natural bond orbital (NBO) analysis [16] was performed with the optimized structures of the zwitterions Na-**1**, Na-**2** and Na-**3** (Table 3) at the B3-LYP/def2-TZVP [17, 18] level of theory using Gaussian 9 code [19].

5.1. Results of calculations for M-1 and [M-1-H]+ (M = Li, Na, K)

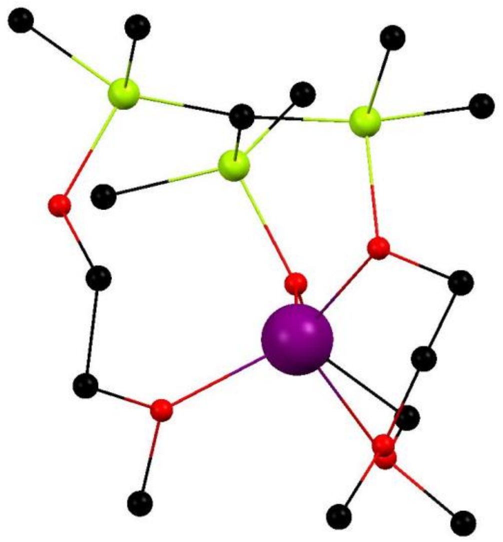

Figure S5. Calculated gas-phase structure of **Li-1**-A (purple = lithium; green = silicon; black = carbon; red = oxygen).

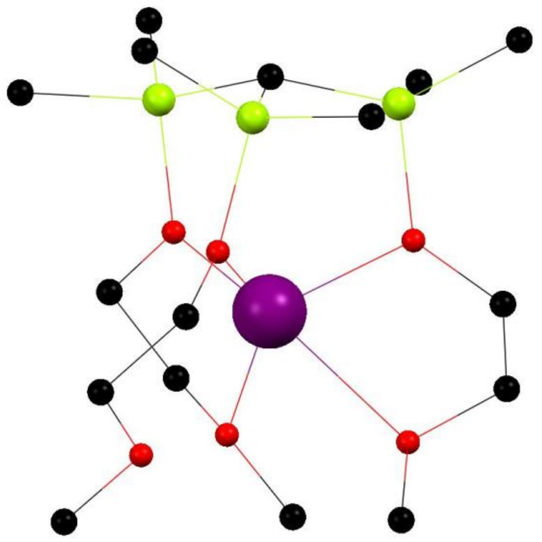

Figure S6. Calculated gas-phase structure of Li-**1**-B (purple = lithium; green = silicon; black = carbon; red = oxygen).

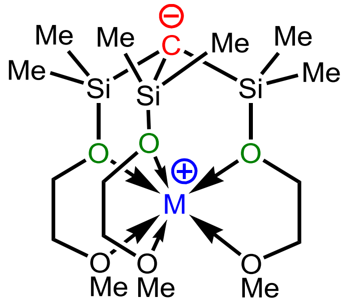

M-**1**

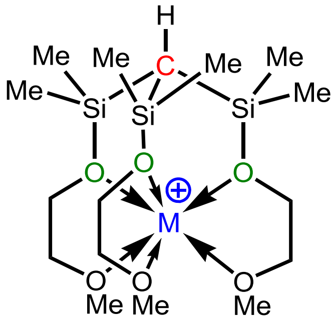

[M-**1**-H]$^+$

Table S6. Calculated average distances [Å] and angles [°] of M-**1** and [M-**1**-H]$^+$ (M = Li, Na, K).

	Li-**1**-A	Li-**1**-B	Na-**1**	K-**1**	[Li-**1**-H]$^+$	[Na-**1**-H]$^+$	[K-**1**-H]$^+$
CN	4	5	6	6	6	6	6
C-Si	1.81	1.81	1.81	1.81	1.90	1.91	1.91
Si-O	1.76	1.76	1.75	1.75	1.71	1.71	1.71
O-M	1.98	2.07	2.38	2.75	2.19	2.42	2.81
C···M	3.03	2.99	3.18	3.34	3.43	3.56	3.88
Si-C-Si	119.5	115.6	117.4	119.2	112.5	114.1	115.5
C-Si-O	-	103.9	105.1	106.6	101.6	103.1	105.1
Si-O-M	-	110.2	109.7	106.8	122.1	120.0	119.6
C-Si-O-M	-	20.8	24.7	27.1	33.5	34.3	37.2
C-H	-	-	-	-	1.10	1.10	1.10

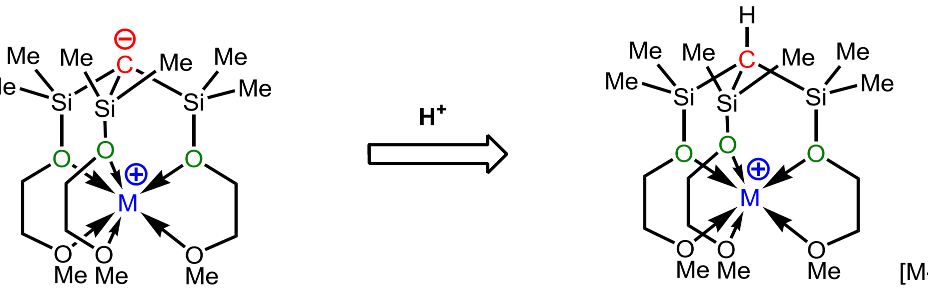

Table S7. Calculated absolute energies [au], HOMO energies [eV], gas-phase basicities [GPB in kcal/mol] and proton affinities [PA in kcal/mol] of M-**1** and [M-**1**-H]$^+$ (M = Li, Na, K).

Comp.	Li-**1**-A	Li-**1**-B	Na-**1**	K-**1**
E	-1960.381346	-1960.381167	-2115.12458	-2552.719709
E_{HOMO}	-4.402	-4.469	-4.335	-4.256
G	-1229864.646	-1229865.514	-1326969.934	-1601567.854
H	-1229795.277	-1229795.459	-1326898.947	-1601494.632
GPB	-282.9	-282.0	-282.3	-280.6
PA	-291.9	-291.8	-290.7	-289.2
Comp.	[Li-**1**-H]$^+$	-	[Na-**1**-H]$^+$	[K-**1**-H]$^+$
E	-1960.857087	-	-2115.598906	-2553.191823
G	-1230153.848	-	-1327258.551	-1601854.708
H	-1230085.719	-	-1327188.14	-1601782.359

Table S8. Calculated NPA charges [e] of M-**1** and $[M\text{-}\mathbf{1}\text{-}H]^+$ (M = Li, Na, K).

Comp.	Li-**1**-A	Li-**1**-B	Na-**1**	K-**1**
e (M)	+0.900	+0.894	+0.917	+0.949
e (C^-)	-2.195	-2.155	-2.168	-2.189
e (Si)	+2.087	+2.081	+2.082	+2.089
e (C)	-1.085	-1.084	-1.086	-1.088
e (O)	-0.977	-0.991	-0.974	-0.966
Comp.	$[Li\text{-}\mathbf{1}\text{-}H]^+$		$[Na\text{-}\mathbf{1}\text{-}H]^+$	$[K\text{-}\mathbf{1}\text{-}H]^+$
e (M)	+0.896		+0.918	+0.953
e (C^-)	-1.774		-1.769	-1.769
e (H)	+0.265		+0.263	+0.261
e (Si)	+2.085		+2.080	+2.082
e (C)	-1.103		-1.103	-1.103
e (O)	-0.980		-0.975	-0.968

5.2. Results of calculations for M-2 and [M-2-H]$^+$ (M = Li, Na, K)

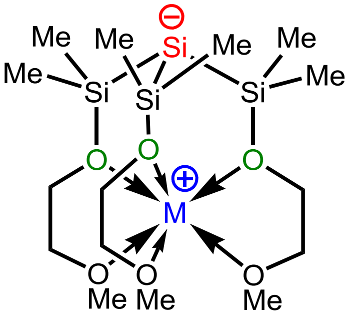

M-**2**

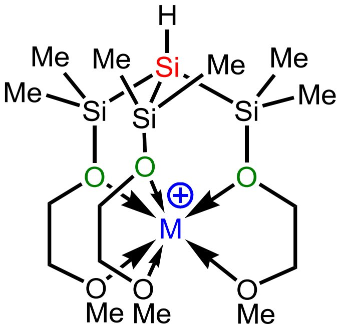

[M-**2**-H]$^+$

Table S9. Calculated average distances [Å] and angles [°] of M-**2** and [M-**2**-H]$^+$ (M = Li, Na, K).

Comp.	Li-**2**	Na-**2**	K-**2**	[Li-**2**-H]$^+$	[Na-**2**-H]$^+$	[K-**2**-H]$^+$
CN (M)	6	6	6	6	6	6
Si-Si	2.35	2.35	2.35	2.36	2.37	2.38
Si-O	1.76	1.75	1.75	1.72	1.72	1.71
O-M	2.07	2.35	2.71	2.27	2.45	2.82
Si···M	3.79	3.94	4.07	3.72	3.74	3.94
Si-Si-Si	97.5	101.2	105.8	107.4	110.0	112.9
Si-Si-O	112.3	113.6	115.7	99.9	101.5	104.1
Si-O-M	120.5	119.9	114.4	128.3	125.6	122.6
Si-Si-O-M	21.9	21.0	21.0	29.2	23.7	24.7
Si-H	-	-	-	1.49	1.50	1.50

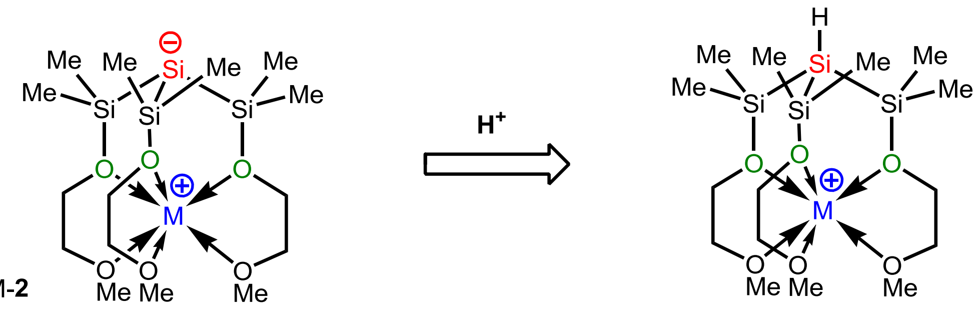

Table S10. Calculated absolute energies [au], HOMO energies [eV], gas-phase basicities [GPB in kcal/mol] and proton affinities [PA in kcal/mol] of M-**2**, and [M-**2**-H]$^+$ (M = Li, Na, K).

Comp.	Li-**2**	Na-**2**	K-**2**
E	-2211.745006	-2366.486518	-2804.077403
E_{HOMO}	-4.200	-4.013	-3.837
G	-1387602.653	-1484706.84	-1759302.046
H	-1387530.78	-1484633.075	-1759225.98
GPB	-274.0	-276.3	-278.9
PA	-281.4	-283.8	-286.3
Comp.	[Li-**2**-H]$^+$	[Na-**2**-H]$^+$	[K-**2**-H]$^+$
E	-2212.201368	-2366.946799	-2804.541748
G	-1387882.999	-1484989.394	-1759587.22
H	-1387810.643	-1484915.362	-1759510.76

Table S11. Calculated NPA charges [e] of M-**2**, and $[M\text{-}\mathbf{2}\text{-}H]^+$ (M = Li, Na, K).

Comp.	Li-**2**	Na-**2**	K-**2**
e (M)	+0.891	+0.913	+0.945
e (E)	-0.683	-0.709	-0.738
e (Si)	+1.584	+1.581	+1.581
e (C)	-1.084	-1.081	-1.080
e (O)	-0.992	-0.978	-0.970
Comp.	$[Li\text{-}\mathbf{2}\text{-}H]^+$	$[Na\text{-}\mathbf{2}\text{-}H]^+$	$[K\text{-}\mathbf{2}\text{-}H]^+$
e (M)	+0.898	+0.917	+0.950
e (E)	-0.139	-0.153	-0.171
e (H)	-0.131	-0.136	-0.139
e (Si)	+1.643	+1.640	+1.643
e (C)	-1.085	-1.084	-1.085
e (O)	-0.976	-0.974	-0.968

5.3. Results of calculations for M-3 and [M-3-H]$^+$ (M = Li, Na, K)

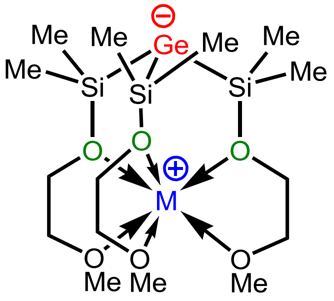

M-**3**

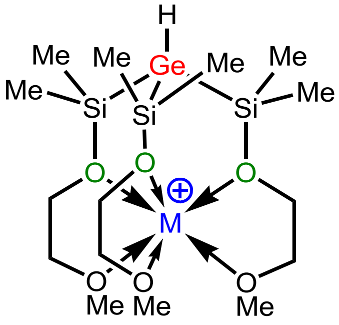

[M-**3**-H]$^+$

Table S12. Calculated average distances [Å] and angles [°] of M-**3** and [M-**3**-H]$^+$ (M = Li, Na, K).

Comp.	Li-**3**	Na-**3**	**K**-3	[Li-**3**-H]$^+$	[Na-**3**-H]$^+$	[K-**3**-H]$^+$
CN (M)	6	6	6	6	6	6
Ge-Si	2.42	2.42	2.42	2.41	2.41	2.42
Si-O	1.76	1.75	1.75	1.72	1.72	1.71
O-M	2.08	2.35	2.71	2.28	2.46	2.82
Ge···M	3.88	4.01	4.14	3.74	3.74	3.93
Si-Ge-Si	95.3	99.1	103.9	107.1	109.7	112.8
Ge-Si-O	112.5	114.0	116.7	99.7	101.1	103.6
Si-O-M	120.5	119.7	115.5	129.0	125.4	122.2
Ge-Si-O-M	27.5	24.8	20.9	27.4	24.8	25.0
Ge-H	-	-	-	1.54	1.55	1.55

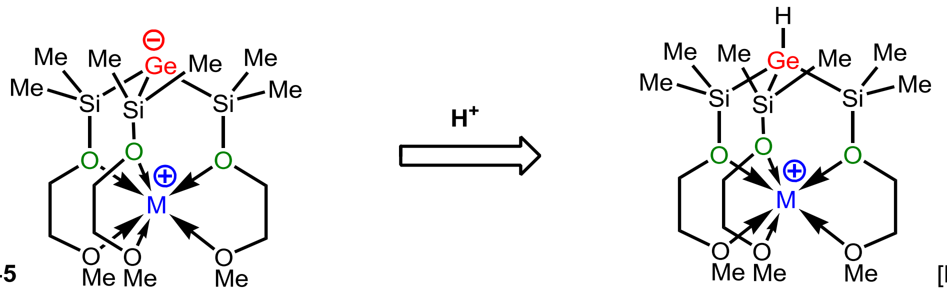

M-**5** [M-**5**-H]$^+$

Table S13. Calculated absolute energies [au], HOMO energies [eV], gas-phase basicities [GPB in kcal/mol] and proton affinities [PA in kcal/mol] of M-**3** and [M-**3**-H]$^+$ (M = Li, Na, K).

Comp.	Li-**3**	Na-**3**	K-**3**
E	-3999.227947	-4153.969879	-4591.560881
E_{HOMO}	-4.260	-4.082	-3.914
E_{HOMO-1}	-5.523	-5.506	-5.504
G	-2509267.985	-2606372.612	-2880967.572
H	-2509194.52	-2606297.148	-2880890.103
GPB	-267.5	-269.1	-272.7
PA	-276.4	-277.8	-280.5
Comp.	[Li-**3**-H]$^+$	[Na-**3**-H]$^+$	[K-**3**-H]$^+$
E	-3999.674531	-4154.420523	-4592.015832
G	-2509541.831	-2606648.037	-2881246.567
H	-2509468.379	-2606573.441	-2881169.138

Table S14. Calculated NPA charges [e] of M-**3**, and $[M\text{-}\mathbf{3}\text{-}H]^+$ (M = Li, Na, K).

Comp.	Li-**3**	Na-**3**	K-**3**
e (M)	+0.890	+0.912	+0.945
e (E)	-0.634	-0.665	-0.698
e (Si)	+1.578	+1.574	+1.574
e (C)	-1.088	-1.085	-1.083
e (O)	-0.993	-0.979	-0.971
Comp.	$[Li\text{-}\mathbf{3}\text{-}H]^+$	$[Na\text{-}\mathbf{3}\text{-}H]^+$	$[K\text{-}\mathbf{3}\text{-}H]^+$
e (M)	+0.899	+0.917	+0.949
e (E)	-0.141	-0.158	-0.179
e (H)	-0.125	-0.127	-0.130
e (Si)	+1.647	+1.647	+1.651
e (C)	-1.088	-1.089	-1.088
e (O)	-0.978	-0.977	-0.971

5.4. Results of calculations for the metal free anions 1^-, 2^-, 3^-, 1, 2-H and 3-H

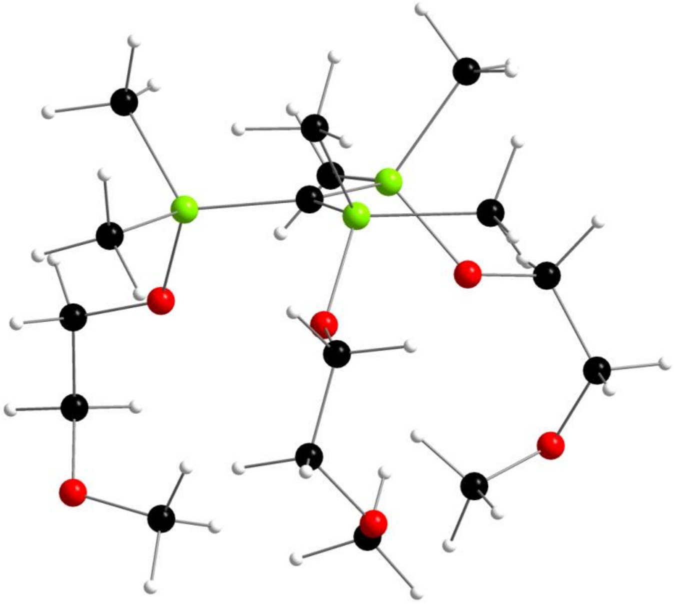

Figure S7. Calculated gas-phase structures of **1**$^-$-A (white = hydrogen; green = silicon; black = carbon; red = oxygen).

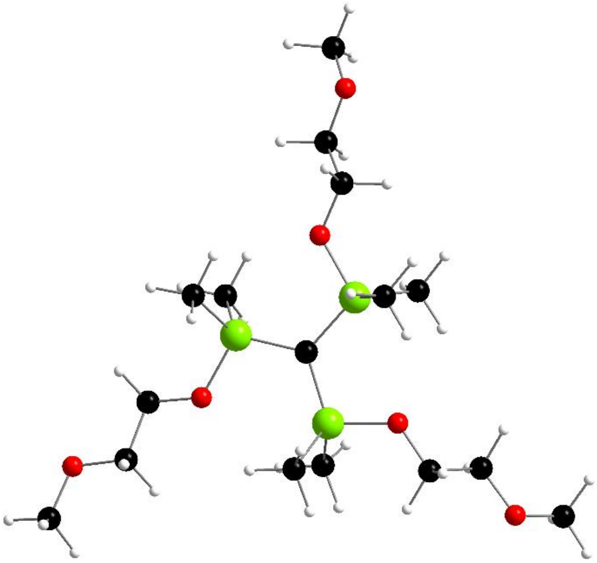

Figure S8. Calculated gas-phase structures of **1**$^-$-B (white = hydrogen; green = silicon; black = carbon; red = oxygen).

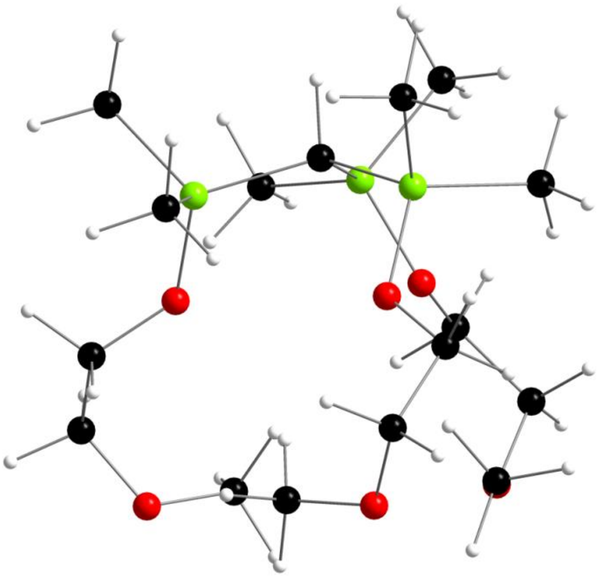

Figure S9. Calculated gas-phase structures of **1**-A (white = hydrogen; green = silicon; black = carbon; red = oxygen).

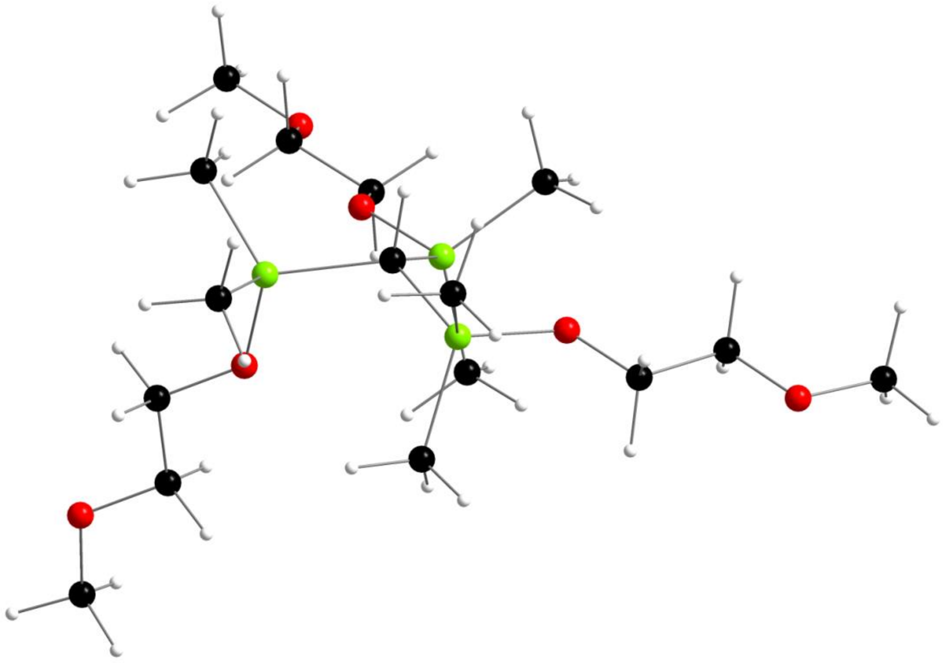

Figure S10. Calculated gas-phase structures of **1**-B (white = hydrogen; green = silicon; black = carbon; red = oxygen).

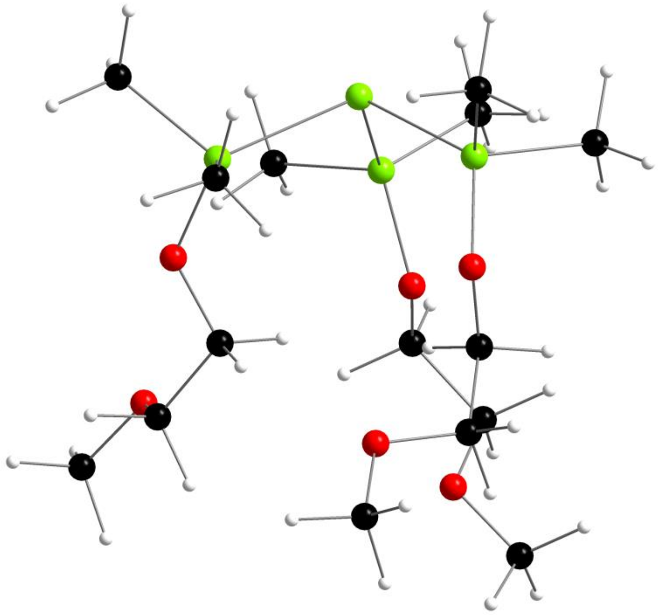

Figure S11. Calculated gas-phase structures of **2**⁻-A (white = hydrogen; green = silicon; black = carbon; red = oxygen).

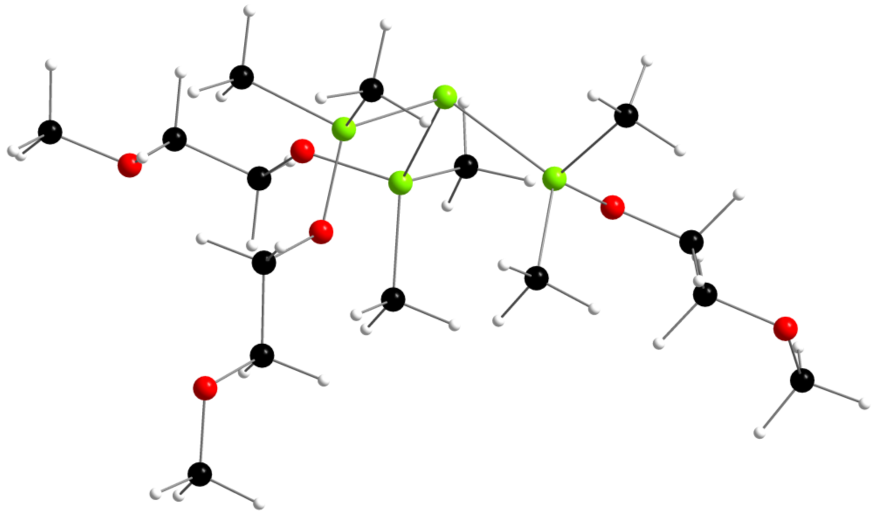

Figure S12. Calculated gas-phase structures of **2**⁻-B (white = hydrogen; green = silicon; black = carbon; red = oxygen).

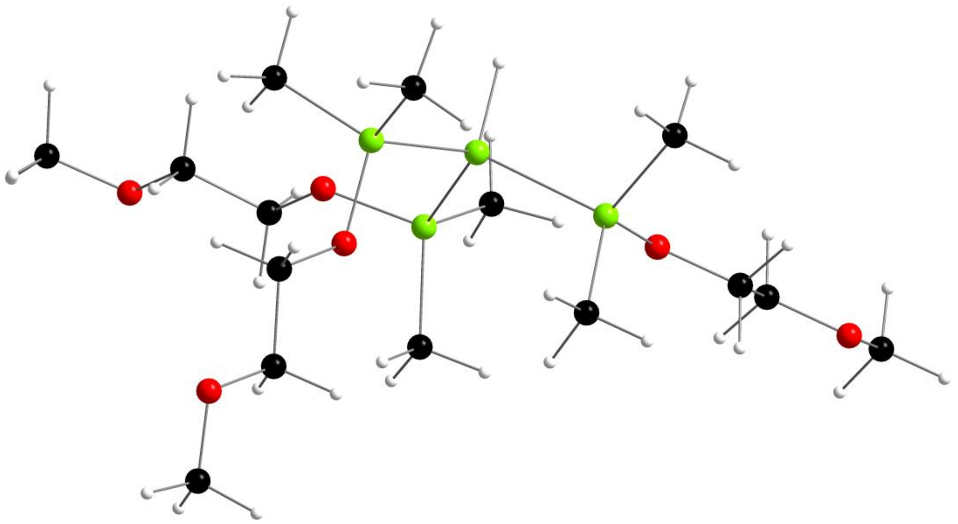

Figure S13. Calculated gas-phase structures of **2**-H-B (white = hydrogen; green = silicon; black = carbon; red = oxygen).

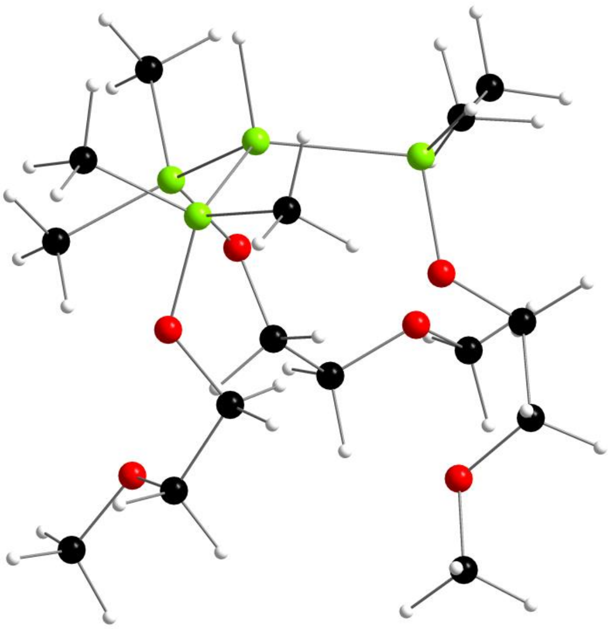

Figure S14. Calculated gas-phase structures of **2**-H-A (white = hydrogen; green = silicon; black = carbon; red = oxygen).

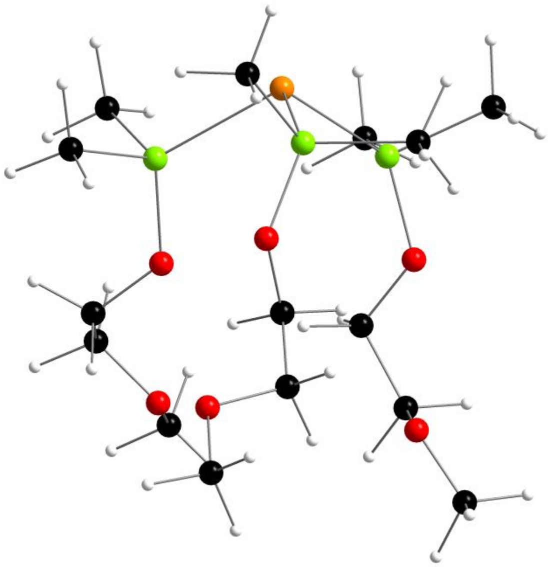

Figure S15. Calculated gas-phase structures of **3**⁻-A (white = hydrogen; green = silicon; black = carbon; red = oxygen; orange = germanium).

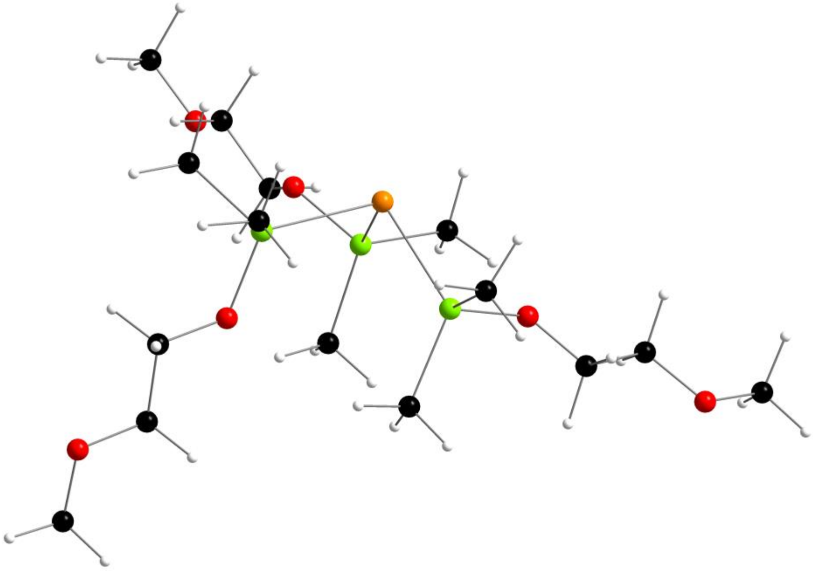

Figure S16. Calculated gas-phase structures of **3**⁻-B (white = hydrogen; green = silicon; black = carbon; red = oxygen; orange = germanium).

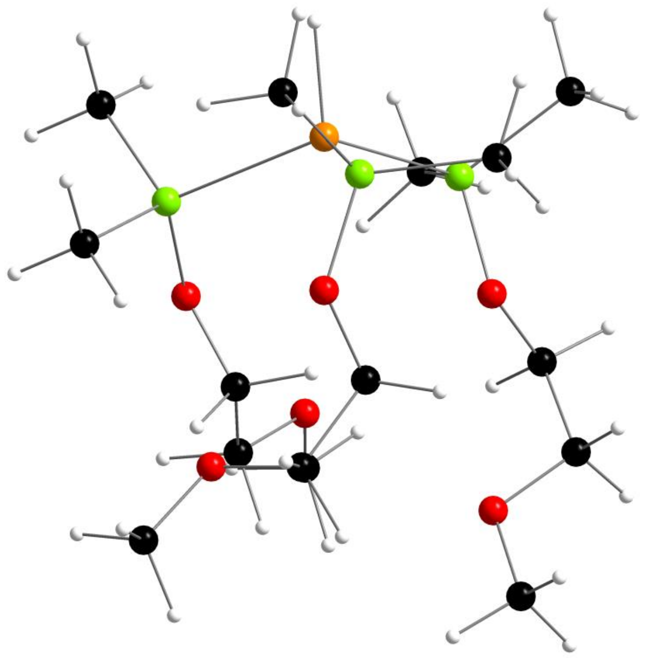

Figure S17. Calculated gas-phase structures of **3**-H-A (white = hydrogen; green = silicon; black = carbon; red = oxygen; orange = germanium).

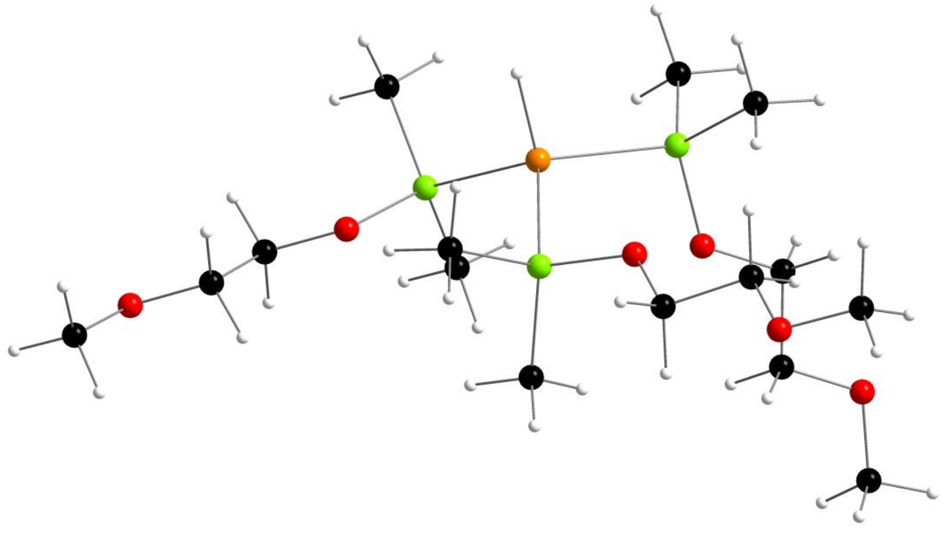

Figure S18. Calculated gas-phase structures of **3**-H-B (white = hydrogen; green = silicon; black = carbon; red = oxygen; orange = germanium).

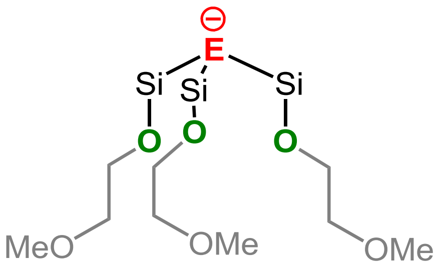

1⁻, **2⁻**, **3⁻**

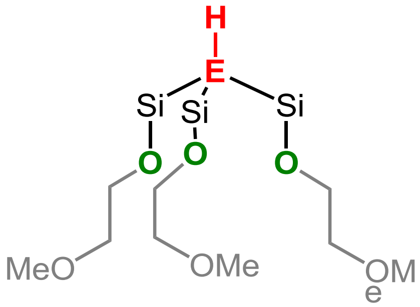

1, **2**-H, **3**-H

Table S15. Calculated average distances [Å] and angles [°] of the metal-free anions, **1**⁻, **2**⁻ and **3**⁻, and their conjugated acids **1**, **2**-H and **3**-H.

Comp.	**1**⁻-A	**1**⁻-B	**2**⁻-A	**2**⁻-B	**3**⁻-A	**3**⁻-B
E-Si	1.81	1.80	2.36	2.36	2.43	2.43
Si-O	1.73	1.73	1.72	1.73	1.72	1.73
Si-E-Si	119.8	120.0	104.9	99.3	103.1	98.2
E-Si-O	110.6	106.9	120.2	110.0	121.6	109.7
Comp.	**1**-A	**1**-B	**2**-H-A	**2**-H-B	**3**-H-A	**3**-H-B
E-H	1.10	1.10	1.51	1.50	1.56	1.55
E-Si	1.91	1.90	2.38	2.37	2.43	2.41
Si-O	1.69	1.69	1.69	1.70	1.69	1.69
Si-E-Si	115.4	114.1	113.3	111.7	114.4	112.1
E-Si-O	106.9	105.3	108.9	104.3	108.5	104.1

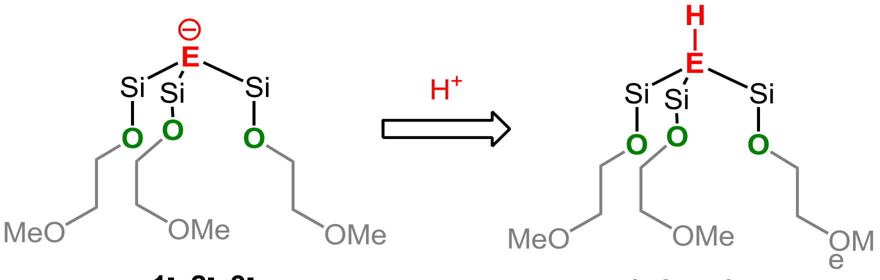

1⁻, **2⁻**, **3⁻** → **1**, **2**-H, **3**-H

Table S16. Calculated absolute energies [au], HOMO energies [eV], gas-phase basicities [GPB in kcal/mol] and proton affinities [PA in kcal/mol] of **1**⁻, **2**⁻, **3**⁻, **1**, **2**-H and **3**-H.

Comp.	**1**⁻-B	**1**⁻-A	**2**⁻-A	**2**⁻-B	**3**⁻-A	**3**⁻-B
E	-1952.81871	-1952.81745	-2204.17386	-2204.17820	-3991.65716	-3991.66274
E_{HOMO}	-0.9176	-1.439	-0.861	-1.111	-0.993	-1.202
G	-1225128.66	-1225125.09	-1382859.48	-1382864.55	-2504523.30	-2504530.00
H	-1225055.01	-1225052.56	-1382783.23	-1382785.84	-2504447.88	-2504450.58
GPB	-348.8	-	-	-339.7	-	-334.9
PA	-352.3	-	-	-351.6	-	-344.3
Comp.	**1**-B	**1**-A	**2**-H-B	**2**-H-A	**3**-H-B	**3**-H-A
E	-1953.39895	-1953.38706	-2204.74599	-2204.74685	-3992.21956	-3992.22140
G	-1225483.69	-1225472.96	-1383214.47	-1383210.52	-2504874.93	-2504871.25
H	-1225408.76	-1225401.41	-1383135.30	-1383135.96	-2504793.36	-2504793.36

Table S17. Calculated NPA charges [e] of **1**$^{-}$, **2**$^{-}$, **3**$^{-}$, **1**, **2**-H and **3**-H.

Comp.	**1**$^{-}$-A	**1**$^{-}$-B	**2**$^{-}$-A	**2**$^{-}$-B	**3**$^{-}$-A	**3**$^{-}$-B
e (E)	-2.188	-2.061	-0.807	-0.709	-0.770	-0.660
e (Si)	+2.106	+1.984	+1.610	+1.596	+1.603	+1.583
e (C)	-1.086	-1.130	-1.084	-1.085	-1.079	-1.084
e (O)	-0.943	-0.871	-0.948	-0.944	-0.949	-0.945
Comp.	**1**-A	**1**-B	**2**-H-A	**2**-H-B	**3**-H-A	**3**-H-B
e (E)	-1.766	-1.762	-0.206	-0.141	-0.220	-0.146
e (H)	+0.249	+0.256	-0.161	-0.156	-0.153	-0.149
e (Si)	+2.096	+2.089	+1.664	+1.650	+1.672	+1.657
e (C)	-1.101	-1.099	-1.082	-1.081	-1.088	-1.085
e (O)	-0.953	-0.950	-0.945	-0.946	-0.946	-0.949

5.5. Results of calculations for the metal free anions 7¯, 8¯, 9¯, 7-H, 8-H and 9-H

Table S18. Calculated av. distances [Å] and angles [°] of ¯E($SiMe_3$)$_3$ and HE($SiMe_3$)$_3$ (E = C, Si, Ge).

Compound	¯C($SiMe_3$)$_3$ (**7**¯)	¯Si($SiMe_3$)$_3$ (**8**¯)	¯Ge($SiMe_3$)$_3$ (**9**¯)
E-Si	1.83	2.38	2.45
Si-CH_3	1.93	1.93	1.93
Si-E-Si	119.7	102.0	100.3
Comp.	HC($SiMe_3$)$_3$ (**7**-H)	HSi($SiMe_3$)$_3$ (**8**-H)	HGe($SiMe_3$)$_3$ (**9**-H)
E-Si	1.92	2.38	2.43
Si-CH_3	1.90	1.90	1.90
Si-E-Si	114.8	113.0	113.5

Table S19. Calculated NPA charges [e] of ¯E($SiMe_3$)$_3$ and HE($SiMe_3$)$_3$ (E = C, Si, Ge).

Compound	¯C($SiMe_3$)$_3$ (**6**¯)	¯Si($SiMe_3$)$_3$ (**7**¯)	¯Ge($SiMe_3$)$_3$ (**8**¯)
e (E)	-2.124	-0.678	-0.654
e (Si)	+1.867	+1.325	+1.322
e (C)	-1.052	-1.042	-1.044
Comp.	HC($SiMe_3$)$_3$ (**6**-H)	HSi($SiMe_3$)$_3$ (**7**-H)	HGe($SiMe_3$)$_3$ (**8**-H)
e (E)	-1.691	-0.105	-0.128
e (H)	+0.248	-0.160	-0.152
e (Si)	+1.813	+1.359	+1.373
e (C)	-1.061	-1.041	-1.044

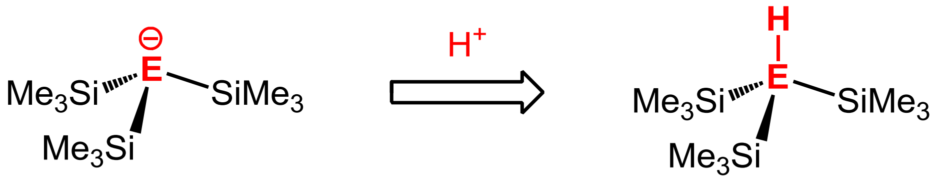

Table S20. Calculated absolute energies [au], HOMO energies [eV], gas-phase basicities [ΔG_B in kcal/mol] and proton affinities [ΔH_{PA} in kcal/mol] of $^{-}E(SiMe_3)_3$ and $HE(SiMe_3)_3$ (E = C, Si, Ge).

Comp.	$^{-}C(SiMe_3)_3$ (**7**$^{-}$)	$^{-}Si(SiMe_3)_3$ (**8**$^{-}$)	$^{-}Ge(SiMe_3)_3$ (**9**$^{-}$)
E	-1265.672843	-1517.049041	-3304.533534
E_{HOMO}	-0.590	-0.682	-0.778
G	-794044.9347	-951790.7426	-2073457.08
H	-793996.7535	-951739.3945	-2073404.219
GPB	-355.1	-349.3	-342.8
PA	-364.1	-356.7	-350.4
Comp.	$HC(SiMe_3)_3$ (**7**-H)	$HSi(SiMe_3)_3$ (**8**-H)	$HGe(SiMe_3)_3$ (**9**-H)
E	-1266.265089	-1517.625792	-3305.100080
G	-794406.2862	-952146.3907	-2073806.2220
H	-794359.3980	-952094.5646	-2073753.1050

5.6. B3-LYP/TZVP Cartesian coordinates [Å]

[Li($MeOCH_2CH_2OSiMe_2)_3$C] (Li-**1**-B)

C	2.8044057	-2.9873036	-0.7144778
Si	1.5040104	-2.0489628	0.3100591
C	1.9498073	-2.4685070	2.1123918
C	-0.2290699	-2.1462751	-0.2051599
Si	-0.5962621	-1.4952465	-1.8539048
C	0.7413739	-1.6200041	-3.2043720
O	1.8835582	-0.3412097	0.1563348
C	3.1307590	0.3073851	0.2636071
C	3.1848193	1.4325389	-0.7524372
O	2.0393972	2.2578648	-0.5686030
C	1.9561665	3.3101221	-1.5169777
O	-0.7974054	0.2370680	-1.6104520
C	-1.2228323	1.1688204	-2.5843193
C	-2.1063653	2.2201460	-1.9446328
O	-1.3690893	2.8836072	-0.9272248
C	-2.1495381	3.8045418	-0.1878972
C	-2.1781274	-2.1059342	-2.7193539
Si	-1.4500194	-1.7527944	1.0725542
C	-1.5044644	-2.8336606	2.6365332
O	-0.9900669	-0.1417067	1.5974935
C	-1.5857254	0.6560065	2.5948484
C	-0.5364795	1.5971406	3.1512199
O	0.0594667	2.2972898	2.0638474
C	1.0740926	3.1967605	2.4800905
C	-3.2796994	-1.5830867	0.5708823
Li	0.1686381	0.7924400	0.1525829

H	-2.5156563	4.6211925	-0.8245945
H	-1.5125681	4.2108718	0.5951933
H	-3.0125962	3.3121729	0.2781088
H	1.8561065	2.6770540	3.0480323
H	0.6597738	3.9968350	3.1070415
H	1.5154855	3.6205257	1.5815604
H	2.7837782	4.0205555	-1.3935809
H	1.0050261	3.8123502	-1.3544239
H	1.9803533	2.9201905	-2.5421826
H	-1.9841297	0.0551348	3.4223250
H	-2.4178199	1.2366359	2.1747318
H	-0.9965253	2.3046462	3.8563496
H	0.2339054	1.0257341	3.6861260
H	2.7679031	-2.7629186	-1.7796538
H	2.6272037	-4.0602117	-0.5971306
H	3.8196438	-2.7884072	-0.3572513
H	3.0234960	-2.3217367	2.2651116
H	1.7258331	-3.5112240	2.3488522
H	1.4221509	-1.8342856	2.8263229
H	-3.0818937	-1.9627216	-2.1282218
H	-2.0741594	-3.1784386	-2.9064514
H	-2.3246204	-1.6226753	-3.6902384
H	-3.8774453	-1.3154412	1.4478824
H	-3.6732295	-2.5260312	0.1847066
H	-3.4358077	-0.8128787	-0.1860273
H	-1.7985421	0.6861230	-3.3832239
H	-0.3555900	1.6540900	-3.0497866
H	3.9692854	-0.3726462	0.0707883
H	3.2598782	0.7152071	1.2748262

H	-2.4422426	2.9395464	-2.7070918
H	-2.9936298	1.7410305	-1.5090455
H	-0.5315665	-2.9658453	3.1073469
H	-1.8768936	-3.8249484	2.3630009
H	-2.1931337	-2.4251564	3.3829016
H	4.1061471	2.0189195	-0.6225386
H	3.1787349	1.0181167	-1.7694576
H	0.3784439	-1.1493102	-4.1232477
H	0.9737864	-2.6608451	-3.4411306
H	1.6682625	-1.1202211	-2.9183602

[Li($MeOCH_2CH_2OSiMe_2$)$_3$C] (Li-**1**-A)

Si	5.0819167	2.4672337	0.4727102
Si	4.5038025	2.0419805	-2.6379024
Si	2.3654294	3.2673071	-0.7177749
O	5.8743148	4.0589987	0.4585579
O	7.2751430	6.5166738	0.1817138
O	4.8271404	3.4520900	-3.5908931
O	4.7790507	6.2081760	-2.2544933
O	2.7854033	4.8167354	-0.0018584
O	3.9155643	7.0362011	0.9226983
C	6.5215031	1.2222707	0.5507478
H	6.1368076	0.2091841	0.4113266
H	7.0091267	1.2487756	1.5292930
H	7.2826141	1.3909077	-0.2113676
C	4.2961936	2.3995938	2.2071859
H	3.8181932	1.4318776	2.3785745
H	3.5446841	3.1775325	2.3498670

H	5.0621315	2.5285648	2.9782798
C	6.9226019	4.4429932	1.3302160
H	7.4634555	3.5698925	1.7122998
H	6.5204624	4.9855707	2.1955333
C	7.9210056	5.3220981	0.6060360
H	8.7580824	5.5623699	1.2785707
H	8.3214337	4.7845983	-0.2641969
C	8.1105013	7.3451245	-0.6053667
H	8.9984196	7.6685650	-0.0463965
H	7.5297034	8.2238470	-0.8849203
H	8.4406529	6.8314360	-1.5171963
C	3.2339917	1.1574813	-3.7337408
H	2.3314890	1.7461229	-3.8963764
H	2.9395606	0.2069228	-3.2809731
H	3.6723112	0.9467235	-4.7128399
C	6.0958814	1.0020447	-2.7789875
H	6.9847917	1.5200050	-2.4133959
H	6.2632351	0.7691399	-3.8345058
H	6.0171855	0.0580949	-2.2358920
C	5.8973619	4.3149151	-3.3103970
H	6.7105000	4.1523729	-4.0333882
H	6.3205407	4.1509155	-2.3086239
C	5.4926414	5.7711421	-3.4208085
H	6.3862025	6.4004914	-3.5398139
H	4.8552048	5.9005055	-4.3026782
C	4.1891786	7.4899623	-2.4378169
H	3.4224328	7.4562284	-3.2198147
H	4.9462726	8.2331519	-2.7172626
H	3.7376700	7.7797247	-1.4914150

C	1.3512940	3.7225264	-2.2599282
H	1.9778095	4.1225200	-3.0588257
H	0.6069636	4.4805199	-1.9959691
H	0.8049884	2.8619015	-2.6515652
C	1.1087265	2.4968452	0.4880474
H	1.5279495	2.3168396	1.4788794
H	0.7908000	1.5300048	0.0880195
H	0.2096329	3.1111565	0.6006801
C	1.9153995	5.8242073	0.4619173
H	1.0154108	5.4097724	0.9318180
H	1.5915460	6.4643608	-0.3691848
C	2.6615732	6.6514441	1.4898715
H	2.0790666	7.5414812	1.7640292
H	2.8414913	6.0593433	2.3962686
C	4.6848334	7.8577509	1.7934400
H	4.1940241	8.8253751	1.9510927
H	5.6563876	7.9979543	1.3270762
H	4.8258306	7.3708347	2.7659600
C	3.9686704	2.4574034	-0.9439292
Li	4.6501675	5.3529370	-0.3914053

$[Li(MeOCH_2CH_2OSiMe_2)_3CH]^+$ [Li-**1**-H]$^+$

C	-0.2624048	2.7352742	-3.1100635
Si	-0.8472632	1.7225967	-1.6289982
C	-2.7338026	1.7421197	-1.6785278
C	-0.0164466	2.2707721	-0.0067111
Si	1.8122772	1.7494118	0.0863716
C	2.7950545	1.7796066	-1.5240891

O	-0.3176996	0.1014013	-1.7544471
C	-0.5254708	-0.7379649	-2.8869115
C	0.6018431	-1.7446409	-2.9582959
O	0.6953715	-2.4029239	-1.6971590
C	1.6767941	-3.4382485	-1.6884902
O	1.6812258	0.1274845	0.6124201
C	2.7760765	-0.6947870	1.0059333
C	2.2807053	-1.7149841	2.0076189
O	1.1592172	-2.3901726	1.4430258
C	0.6777048	-3.4467564	2.2721245
C	2.7854233	2.7806223	1.3313709
Si	-1.0042653	1.7401334	1.5314437
C	-2.5891405	2.7419154	1.7442662
O	-1.3647196	0.1105675	1.1593403
C	-2.2434283	-0.7261828	1.9055714
C	-2.8529266	-1.7469823	0.9696672
O	-1.7969121	-2.4054683	0.2735347
C	-2.2651389	-3.4626904	-0.5624235
C	-0.1036097	1.7936870	3.1883266
Li	0.0102470	-1.1589131	0.0073910
H	1.4389875	-4.2248553	2.3911627
H	-0.2003535	-3.8611497	1.7843777
H	0.3978286	-3.0716072	3.2628307
H	-2.9907788	-3.0921688	-1.2953082
H	-2.7373811	-4.2499420	0.0344414
H	-1.4005981	-3.8641408	-1.0843439
H	1.4119702	-4.2308880	-2.3959536
H	1.7083110	-3.8401677	-0.6794749
H	2.6648807	-3.0448364	-1.9525510

H	-3.0526536	-0.1544914	2.3720595
H	-1.6836262	-1.2303559	2.7002561
H	-3.4411879	-2.4705589	1.5479178
H	-3.5202275	-1.2582683	0.2485207
H	0.8150463	2.7043076	-3.2666598
H	-0.5481722	3.7813557	-2.9654914
H	-0.7493888	2.4034400	-4.0307660
H	-3.0693280	1.3841857	-2.6562240
H	-3.1254553	2.7554643	-1.5615693
H	-3.1856736	1.1079426	-0.9162576
H	2.3829287	2.7429381	2.3427082
H	2.7839558	3.8265546	1.0110046
H	3.8317678	2.4670642	1.3693002
H	-0.7800829	1.4461164	3.9745611
H	0.1876503	2.8135576	3.4506475
H	0.7855485	1.1648051	3.2071915
H	3.5772071	-0.1107029	1.4712819
H	3.1909376	-1.2000536	0.1276820
H	-0.5408224	-0.1655136	-3.8204153
H	-1.4862353	-1.2540576	-2.7902306
H	3.0835064	-2.4275060	2.2341496
H	1.9820657	-1.2232185	2.9418764
H	-3.2619286	2.6825184	0.8898014
H	-2.3339964	3.7950200	1.8946205
H	-3.1400930	2.4246005	2.6334633
H	0.3977717	-2.4695230	-3.7562110
H	1.5515785	-1.2431230	-3.1831890
H	3.8152931	1.4374603	-1.3277615
H	2.8726770	2.7932837	-1.9244803

H	2.3674073	1.1365556	-2.2927176
H	-0.0220404	3.3694421	-0.0134378

[Na(MeOCH$_2$CH$_2$OSiMe$_2$)$_3$C] (Na-**1**)

C	2.7202967	-2.8437979	-1.5066044
Si	1.7799162	-1.8251656	-0.2020030
C	2.8908534	-1.9005479	1.3441689
C	0.0084393	-2.1345868	0.0126983
Si	-1.0659571	-1.8549662	-1.4182682
C	-0.2819299	-1.9562483	-3.1521224
O	1.8929297	-0.1585814	-0.7344601
C	3.0535719	0.5926642	-0.9949760
C	2.6993035	1.7625645	-1.8912078
O	1.6760402	2.5443118	-1.2733080
C	1.2815097	3.6469868	-2.0738111
O	-1.5927230	-0.1889447	-1.2777327
C	-2.3711331	0.5547435	-2.1831728
C	-2.9850395	1.7357902	-1.4573366
O	-1.9493328	2.5193417	-0.8613058
C	-2.4588930	3.6330792	-0.1449575
C	-2.6595338	-2.8859180	-1.5602031
Si	-0.6950180	-1.8459198	1.6564766
C	-0.0184468	-2.8644308	3.1151682
O	-0.3144786	-0.1761850	2.0258220
C	-0.6777516	0.5684543	3.1622468
C	0.2658204	1.7459378	3.3070822
O	0.2323297	2.5316150	2.1142019
C	1.1168688	3.6393985	2.1712266

C	-2.5880432	-1.9498752	1.8474981
Na	-0.0157671	1.0457949	0.0055890
H	-2.9781069	4.3312690	-0.8137418
H	-1.6141902	4.1387919	0.3197739
H	-3.1536655	3.3159008	0.6419146
H	2.1497802	3.3153951	2.3479028
H	0.8256111	4.3388295	2.9649116
H	1.0715182	4.1456647	1.2085306
H	2.1128569	4.3481612	-2.2206383
H	0.4687627	4.1537854	-1.5566017
H	0.9201970	3.3174192	-3.0555937
H	-0.6164856	-0.0237635	4.0857289
H	-1.7099087	0.9356828	3.0749959
H	-0.0310174	2.3610861	4.1685070
H	1.2884561	1.3831214	3.4741586
H	2.2651691	-2.7963080	-2.4955889
H	2.7323554	-3.8927522	-1.1981260
H	3.7619791	-2.5192264	-1.5956336
H	3.9075853	-1.5906453	1.0834309
H	2.9541762	-2.9174349	1.7385053
H	2.5350259	-1.2464564	2.1424348
H	-3.2798829	-2.8357666	-0.6658863
H	-2.3932538	-3.9344011	-1.7203255
H	-3.2674578	-2.5705316	-2.4142898
H	-2.8744440	-1.6606747	2.8634539
H	-2.9462205	-2.9696575	1.6890259
H	-3.1109573	-1.2923039	1.1508730
H	-3.1927001	-0.0369515	-2.6102139
H	-1.7580212	0.9178826	-3.0198384

H	3.8252253	0.0022764	-1.5079366
H	3.4933458	0.9681850	-0.0605284
H	-3.5602736	2.3516854	-2.1631181
H	-3.6651641	1.3772490	-0.6738158
H	1.0662937	-2.8128432	3.2054194
H	-0.2887117	-3.9141945	2.9707527
H	-0.4542710	-2.5446280	4.0671630
H	3.5895668	2.3836052	-2.0661645
H	2.3383768	1.3917077	-2.8593891
H	-1.0207223	-1.6797954	-3.9109301
H	0.0473074	-2.9731181	-3.3782134
H	0.5750772	-1.2887821	-3.2578948

$[Na(MeOCH_2CH_2OSiMe_2)_3CH]^+$ [Na-**1**-H]$^+$

C	-3.0234777	2.8958868	0.5904690
Si	-1.5439070	1.8032548	1.0137335
C	-1.3498292	1.8416603	2.8905732
C	0.0010428	2.2755984	0.0006349
Si	-0.1017151	1.8039823	-1.8436091
C	-1.8226425	1.8366401	-2.6174635
O	-1.8442958	0.1905162	0.5336810
C	-2.9282890	-0.6026557	1.0129435
C	-3.2362323	-1.7029200	0.0209224
O	-2.0696937	-2.5004785	-0.1758056
C	-2.3017960	-3.6145770	-1.0357151
O	0.4669553	0.1915668	-1.8650896
C	0.5919103	-0.5971521	-3.0468729
C	1.6032854	-1.7001632	-2.8246910

O	1.1900039	-2.5041419	-1.7204583
C	2.0479860	-3.6226492	-1.5021372
C	1.0028754	2.9001396	-2.9108349
Si	1.6498220	1.8021287	0.8329347
C	2.0308282	2.9050569	2.3161362
O	1.3778992	0.1935361	1.3436430
C	2.3326086	-0.5934026	2.0539570
C	1.6250966	-1.6964414	2.8099731
O	0.8863275	-2.4991366	1.8898183
C	0.2419880	-3.6063140	2.5182908
C	3.1802596	1.8229763	-0.2713084
Na	0.0065124	-1.2832115	-0.0009362
H	2.0311599	-4.3005214	-2.3617472
H	1.6825109	-4.1448538	-0.6201677
H	3.0794111	-3.2994460	-1.3236836
H	-0.4382821	-3.2698914	3.3082455
H	0.9784823	-4.2920580	2.9496538
H	-0.3324889	-4.1247551	1.7529982
H	-3.0328315	-4.3009291	-0.5958779
H	-1.3515949	-4.1290081	-1.1641958
H	-2.6655329	-3.2866791	-2.0155501
H	2.8911672	0.0095405	2.7783803
H	3.0520819	-1.0284166	1.3513251
H	2.3707442	-2.3083328	3.3335919
H	0.9437606	-1.2699923	3.5573519
H	-3.3187645	2.8384787	-0.4566064
H	-2.7870242	3.9402107	0.8146693
H	-3.8910837	2.6330761	1.2014410
H	-2.2828348	1.5165610	3.3596799

H	-1.1543000	2.8558435	3.2470769
H	-0.5492633	1.1919119	3.2440347
H	2.0540448	2.8617738	-2.6277959
H	0.6730315	3.9401824	-2.8345044
H	0.9261702	2.6233411	-3.9655740
H	4.0486618	1.4958649	0.3078292
H	3.4000040	2.8330720	-0.6247738
H	3.0857698	1.1675840	-1.1370484
H	0.9298877	0.0050272	-3.8976281
H	-0.3789423	-1.0327367	-3.3071897
H	-3.8360163	-0.0036417	1.1468370
H	-2.6678088	-1.0410534	1.9826341
H	1.6707782	-2.3118752	-3.7335847
H	2.5939615	-1.2742069	-2.6213825
H	1.2651364	2.8731308	3.0905828
H	2.1287465	3.9430951	1.9854221
H	2.9851231	2.6301608	2.7732873
H	-4.0552192	-2.3198573	0.4129106
H	-3.5560529	-1.2737398	-0.9368577
H	-1.7564814	1.5183764	-3.6616754
H	-2.2371082	2.8474716	-2.6210543
H	-2.5258233	1.1779051	-2.1075329
H	0.0009125	3.3760679	0.0009126

$[K(MeOCH_2CH_2OSiMe_2)_3C]$ (K-**1**)

C	2.5896182	2.9978862	1.5716224
Si	1.7850869	1.8127430	0.3155921
C	2.9423037	1.8658518	-1.1989014

C	0.0092275	1.9802557	-0.0063843
Si	-1.1568494	1.8326282	1.3735737
C	-0.4210551	1.9117153	3.1307890
O	2.0229041	0.2135849	0.9870155
C	3.2566651	-0.3882256	1.2912597
C	3.0416203	-1.6200665	2.1468541
O	2.2622706	-2.5858517	1.4355450
C	2.0989681	-3.7896042	2.1653958
O	-1.8571229	0.2319936	1.2674707
C	-2.7314632	-0.3581985	2.1965785
C	-3.3817990	-1.5873320	1.5943197
O	-2.3880581	-2.5625182	1.2665767
C	-2.9554445	-3.7585403	0.7598785
C	-2.6466988	3.0186987	1.4252897
Si	-0.6029774	1.8176414	-1.7046460
C	0.1037976	2.9870551	-3.0321649
O	-0.1700145	0.2082382	-2.2401281
C	-0.5279828	-0.3897369	-3.4610337
C	0.3176234	-1.6226479	-3.7069115
O	0.0916097	-2.5923264	-2.6802947
C	0.7943667	-3.7999057	-2.9175833
C	-2.4919892	1.9064573	-1.9519253
K	-0.0071551	-1.3617746	0.0057529
H	-3.6302195	-4.2208993	1.4913642
H	-2.1380198	-4.4494991	0.5527924
H	-3.5139219	-3.5783637	-0.1674699
H	1.8792981	-3.6367172	-2.9434637
H	0.4867030	-4.2611906	-3.8647120
H	0.5585931	-4.4843057	-2.1024200

H	3.0667801	-4.2580349	2.3846478
H	1.5119755	-4.4717670	1.5498934
H	1.5699868	-3.6183788	3.1116356
H	-0.3710640	0.2864513	-4.3135205
H	-1.5887949	-0.6785783	-3.4644210
H	0.0547535	-2.0548874	-4.6831267
H	1.3807458	-1.3483074	-3.7238662
H	2.1128793	2.9598182	2.5513636
H	2.5094120	4.0238205	1.2023940
H	3.6544744	2.7815425	1.7052171
H	3.9589461	1.5902568	-0.9024000
H	2.9929973	2.8721842	-1.6209878
H	2.6233651	1.1816454	-1.9878155
H	-3.2488805	2.9774179	0.5175982
H	-2.2864132	4.0448270	1.5384738
H	-3.3012007	2.8068238	2.2767619
H	-2.7482485	1.6419929	-2.9825837
H	-2.8631625	2.9193319	-1.7794383
H	-3.0299400	1.2294825	-1.2855473
H	-3.5407153	0.3244015	2.4937964
H	-2.1977959	-0.6501198	3.1123211
H	3.9172950	0.2869144	1.8543029
H	3.7913586	-0.6800155	0.3760453
H	-4.0917062	-2.0152882	2.3168460
H	-3.9351504	-1.3094991	0.6873662
H	1.1910370	2.9426301	-3.0986705
H	-0.1724114	4.0164003	-2.7876425
H	-0.3048438	2.7666581	-4.0236817
H	4.0165330	-2.0558748	2.4085478

H	2.5245509	-1.3438495	3.0754247
H	-1.1864224	1.6597298	3.8712637
H	-0.0686128	2.9187507	3.3650282
H	0.4145462	1.2209086	3.2593326

$[K(MeOCH_2CH_2OSiMe_2)_3CH]^+$ [K-**1**-H]$^+$

C	-2.1917959	-3.0336190	-2.0758269
Si	-0.8086450	-1.8102760	-1.6795210
C	0.3568422	-1.7885463	-3.1646464
C	0.0019766	-2.2191558	-0.0019419
Si	-1.0509207	-1.8067955	1.5345378
C	-2.9196154	-1.8281496	1.2656321
O	-1.4546861	-0.2393792	-1.4903045
C	-2.1854050	0.4137489	-2.5289163
C	-3.0790337	1.4990384	-1.9705546
O	-2.2948444	2.5354239	-1.3815418
C	-3.0753789	3.6780948	-1.0362393
O	-0.5924935	-0.2214510	1.9754183
C	-1.1598903	0.4564285	3.0952197
C	-0.2248830	1.5236133	3.6215690
O	-0.0305913	2.5409940	2.6398798
C	0.7024846	3.6542616	3.1471984
C	-0.6796230	-3.0001386	2.9506870
Si	1.8593405	-1.8086884	0.1381426
C	2.8972504	-3.0068361	-0.8887818
O	2.0164120	-0.2256130	-0.4846447
C	3.2727539	0.4503150	-0.5376500
C	3.2736535	1.5246688	-1.6026648

O	2.3276240	2.5427603	-1.2812703
C	2.4297394	3.6733983	-2.1444922
C	2.5642979	-1.8231764	1.8898538
K	-0.0077054	1.6648572	-0.0189207
H	0.1718870	4.1259602	3.9808880
H	0.8026393	4.3762732	2.3377227
H	1.7003196	3.3526245	3.4846978
H	2.2407801	3.3958272	-3.1874840
H	3.4209320	4.1335252	-2.0718747
H	1.6802713	4.3968436	-1.8262801
H	-3.5472030	4.1134574	-1.9233728
H	-2.4023247	4.4135231	-0.5974318
H	-3.8528187	3.4228999	-0.3076091
H	4.0868883	-0.2439595	-0.7744021
H	3.4944153	0.9040339	0.4352219
H	4.2815802	1.9572464	-1.6530624
H	3.0375209	1.0906743	-2.5828222
H	-2.9885965	-3.0393651	-1.3322652
H	-1.7809489	-4.0454695	-2.1350572
H	-2.6409326	-2.8196866	-3.0491428
H	-0.1948671	-1.4851870	-4.0589971
H	0.7602817	-2.7835245	-3.3657767
H	1.1912015	-1.0994105	-3.0303906
H	0.3589970	-2.9662939	3.2785988
H	-0.8999998	-4.0253729	2.6392247
H	-1.3134767	-2.7933934	3.8169238
H	3.6181153	-1.5317981	1.8630214
H	2.5254769	-2.8243066	2.3251081
H	2.0400415	-1.1374182	2.5562211

H	-1.3591475	-0.2371548	3.9199935
H	-2.1127742	0.9174784	2.8103770
H	-2.8274776	-0.2938333	-3.0658464
H	-1.4923820	0.8532856	-3.2556534
H	-0.6732565	1.9588996	4.5242058
H	0.7412136	1.0817724	3.8973970
H	2.6441633	-2.9939323	-1.9485564
H	2.7568268	-4.0277393	-0.5221759
H	3.9633127	-2.7841499	-0.7945416
H	-3.6759681	1.9097210	-2.7953659
H	-3.7677679	1.0811710	-1.2249395
H	-3.4264156	-1.5281160	2.1871961
H	-3.2730328	-2.8333302	1.0248567
H	-3.2337071	-1.1528074	0.4695668
H	0.0025411	-3.3211980	-0.0009462

[Li($MeOCH_2CH_2OSiMe_2)_3Si$] (Li-**2**)

C	-0.2337487	1.6121749	3.5250142
H	-1.0814468	0.9260450	3.4701790
H	-0.6092729	2.5792193	3.8690372
H	0.4579742	1.2361453	4.2855749
C	2.2188527	2.7978524	2.1831183
H	2.8130489	2.3900456	3.0065677
H	1.9336970	3.8156780	2.4624474
H	2.8592157	2.8721653	1.3017168
C	2.3972647	2.4031385	-1.7765197
H	2.9543745	1.9796647	-0.9383976
H	2.5134943	3.4893564	-1.7400319

H	2.8606680	2.0518510	-2.7038921
C	-0.1870108	2.4956455	-3.3687062
H	0.3793113	2.1429000	-4.2360476
H	-0.1753023	3.5885488	-3.3956588
H	-1.2256091	2.1798430	-3.4858861
C	-3.4225067	1.1073520	-1.5060022
H	-2.8142431	0.7664559	-2.3462704
H	-3.8208531	2.0927223	-1.7605734
H	-4.2716232	0.4240554	-1.4029386
C	-3.6695836	1.5131969	1.4952772
H	-4.5100372	0.8125047	1.4809734
H	-4.0825271	2.5173551	1.3669324
H	-3.2084129	1.4768875	2.4843864
C	2.1077286	-0.5445331	2.1861190
H	1.5875491	-1.2683178	2.8249028
H	2.6936643	0.1113590	2.8386883
C	3.0664245	-1.2724058	1.2679129
H	3.6470384	-0.5502355	0.6786084
H	3.7665953	-1.8760659	1.8639767
C	1.0492763	-0.5738960	-2.8399122
H	2.0653454	-0.9180865	-2.6137796
H	1.0985636	0.0261499	-3.7551276
C	0.1567695	-1.7660047	-3.1068710
H	-0.8453073	-1.4286553	-3.4033959
H	0.5752800	-2.3696986	-3.9259928
C	3.1166826	-2.8580754	-0.4915521
H	3.7588897	-2.2049341	-1.0958180
H	2.4430158	-3.4033272	-1.1486910
H	3.7549493	-3.5645037	0.0548540

C	-0.7281978	-3.6978497	-2.0610496
H	-1.7443676	-3.4420825	-2.3870004
H	-0.7771296	-4.1760039	-1.0854231
H	-0.2921503	-4.3903520	-2.7927325
C	-0.0940308	-3.7148054	2.0622980
H	-0.0331013	-3.3011184	3.0767144
H	0.9118480	-3.8670183	1.6781342
H	-0.6247165	-4.6744281	2.1087829
C	-2.6607197	-1.5460654	0.5658763
H	-2.7934137	-2.0945457	-0.3747427
H	-3.6536754	-1.2426419	0.9150566
C	-2.0525889	-2.4609444	1.6074776
H	-1.9995776	-1.9490551	2.5774646
H	-2.6747848	-3.3604644	1.7230207
Li	0.1664040	-0.9179840	-0.0246362
O	1.1649460	0.1817011	1.4153429
O	0.5381216	0.1718682	-1.7474567
O	-1.8093602	-0.4324333	0.3587261
O	2.3091308	-2.1055892	0.3984498
O	0.0728497	-2.5377434	-1.9152074
O	-0.7431528	-2.8157528	1.1786638
Si	-0.6593039	2.7807586	0.1258670
Si	0.6275572	1.8000254	1.8310631
Si	0.5499371	1.9268426	-1.6999100
Si	-2.3988538	1.1999280	0.1033130

$[Li(MeOCH_2CH_2OSiMe_2)_3SiH]^+$ [Li-**2**-H]$^+$

C	1.8213095	2.4731613	3.0571727

Si	1.7487364	1.6826084	1.3408020
C	3.4702028	1.7325650	0.5638485
Si	0.0015453	2.5513344	0.0056475
Si	-2.0309752	1.6879576	0.8488169
C	-2.2147624	1.7208916	2.7295775
O	1.2353077	0.0469475	1.4693261
C	1.8149973	-0.8774771	2.3905796
C	0.7385631	-1.7970488	2.9193043
O	0.0825991	-2.4101744	1.8113415
C	-0.8232989	-3.4381293	2.2103582
O	-1.8932254	0.0573915	0.3221686
C	-2.9807891	-0.8665778	0.3726522
C	-2.9080528	-1.7879016	-0.8227796
O	-1.6235114	-2.4077546	-0.8398996
C	-1.5191702	-3.4252578	-1.8345883
C	-3.5521129	2.4937313	0.0666012
Si	0.2831967	1.6833421	-2.1756100
C	1.7202070	2.4903296	-3.1025959
O	0.6711719	0.0510786	-1.7995818
C	1.1871950	-0.8626591	-2.7680223
C	2.1755148	-1.7915794	-2.1018237
O	1.5303592	-2.4232158	-0.9976871
C	2.3258133	-3.4534200	-0.4127716
C	-1.2564331	1.7192444	-3.2704233
Li	0.0016053	-1.1667624	-0.0071144
H	-2.2318960	-4.2331445	-1.6385347
H	-0.5044344	-3.8110436	-1.7918577
H	-1.7132607	-3.0193177	-2.8335127
H	3.2851969	-3.0574623	-0.0620610

H	2.5181432	-4.2522735	-1.1364168
H	1.7674087	-3.8479755	0.4315988
H	-0.2911595	-4.2447008	2.7253224
H	-1.2887102	-3.8220570	1.3068082
H	-1.5958983	-3.0433620	2.8795960
H	1.6993275	-0.3425779	-3.5840865
H	0.3630770	-1.4378260	-3.2009679
H	2.5150605	-2.5430588	-2.8258554
H	3.0508800	-1.2328088	-1.7480716
H	0.8798834	2.3741345	3.6003855
H	2.0298296	3.5412334	2.9502422
H	2.6210213	2.0522110	3.6722314
H	4.2065632	1.2604922	1.2205543
H	3.7926769	2.7643220	0.4026433
H	3.4913200	1.2165252	-0.3970942
H	-3.5488811	2.4166421	-1.0220876
H	-3.5660424	3.5567809	0.3220498
H	-4.4851550	2.0638248	0.4403520
H	-1.0573656	1.2506320	-4.2386140
H	-1.5665349	2.7489390	-3.4661636
H	-2.0932191	1.1959420	-2.8053456
H	-3.9491650	-0.3557506	0.3560168
H	-2.9223835	-1.4462780	1.2989519
H	2.2808319	-0.3669626	3.2396908
H	2.5896917	-1.4592539	1.8818113
H	-3.6970911	-2.5472182	-0.7475623
H	-3.0589506	-1.2247186	-1.7521140
H	2.6656712	2.3982291	-2.5647894
H	1.5134566	3.5570371	-3.2248446

H	1.8513789	2.0739848	-4.1049351
H	1.1945279	-2.5606111	3.5624741
H	0.0094977	-1.2347420	3.5158122
H	-3.1556420	1.2555982	3.0376043
H	-2.2257626	2.7497834	3.0980552
H	-1.3973373	1.1935804	3.2240985
H	0.0045238	4.0455693	0.0031252

$[Na(MeOCH_2CH_2OSiMe_2)_3Si]$ (Na-**2**)

Na	-0.0011619	-1.1119807	-0.0061828
Si	-0.0041744	2.8252784	0.0027447
Si	-2.0004568	1.7587392	0.6427387
Si	1.5570954	1.7683392	1.4088576
Si	0.4399141	1.7665872	-2.0504366
O	-2.0230739	0.0456935	0.2888345
O	1.2858695	0.0494410	1.5856037
O	0.7604353	0.0534666	-1.9010336
O	-1.9681139	-2.6064516	-0.5810191
O	0.4812874	-2.5876915	1.9857317
O	1.4695603	-2.5979781	-1.4162315
C	-2.4113142	1.8858114	2.5037844
C	-3.5145754	2.4809119	-0.2704669
C	1.5094895	2.4697143	3.1853146
C	3.3734869	1.9300484	0.8408647
C	-0.9706915	1.8984004	-3.3317188
C	1.9856259	2.4943336	-2.9049775
C	-3.1216161	-0.8130827	0.5224891
C	-3.1895355	-1.8683683	-0.5624546

C	2.0395276	-0.8122384	2.4149201
C	1.1319101	-1.8600432	3.0267036
C	1.1260130	-0.7990487	-2.9679249
C	2.0866787	-1.8602384	-2.4706983
C	-1.9372020	-3.5888127	-1.6055749
C	-0.4193257	-3.5670020	2.4809114
C	2.3259332	-3.5856109	-0.8621493
H	-3.3998061	1.4715944	2.7277778
H	-2.4131620	2.9319417	2.8197924
H	-1.6757878	1.3547569	3.1117159
H	-3.4069743	2.4044003	-1.3543845
H	-3.6055980	3.5422856	-0.0255184
H	-4.4537008	1.9970478	0.0160353
H	0.5204038	2.3659211	3.6365845
H	1.7428230	3.5371367	3.1529130
H	2.2396091	1.9950105	3.8486011
H	4.0645320	1.5112772	1.5794293
H	3.6356164	2.9820184	0.7027060
H	3.5418439	1.4176340	-0.1089425
H	-0.6704670	1.4924901	-4.3029265
H	-1.2502779	2.9440636	-3.4826693
H	-1.8611700	1.3597496	-3.0000195
H	2.8750400	2.4029343	-2.2779038
H	1.8230089	3.5598220	-3.0870098
H	2.1985756	2.0260146	-3.8712853
H	-4.0761045	-0.2726132	0.5207451
H	-3.0217666	-1.3001922	1.5017502
H	-4.0341043	-2.5454220	-0.3707048
H	-3.3425267	-1.3889256	-1.5380424

H	2.5276330	-0.2744525	3.2368000
H	2.8288687	-1.3052006	1.8315438
H	1.7226741	-2.5455337	3.6511973
H	0.3777385	-1.3748525	3.6598687
H	1.6230232	-0.2549639	-3.7804361
H	0.2347641	-1.2806179	-3.3916094
H	2.3543687	-2.5362671	-3.2952708
H	3.0036139	-1.3860099	-2.0974700
H	-2.6995721	-4.3596233	-1.4370901
H	-0.9477989	-4.0418010	-1.5905727
H	-2.1048932	-3.1380703	-2.5913255
H	0.1122090	-4.3478905	3.0390489
H	-0.9223635	-4.0077154	1.6221918
H	-1.1717627	-3.1157912	3.1394876
H	2.5730660	-4.3551412	-1.6040242
H	1.7988730	-4.0389580	-0.0249231
H	3.2579665	-3.1401671	-0.4936074

$[Na(MeOCH_2CH_2OSiMe_2)_3SiH]^+$ [Na-**2**-H]$^+$

C	1.5825339	2.5898558	3.1941192
Si	1.6827020	1.7769396	1.4903151
C	3.4429100	1.9445176	0.8223576
Si	0.0003978	2.5473703	0.0042112
Si	-2.1301011	1.7770578	0.7113896
C	-2.4294388	1.9138714	2.5726613
O	1.2851218	0.1125714	1.6198529
C	2.0029448	-0.8070826	2.4452792
C	1.0523465	-1.8185223	3.0468724

O	0.3737220	-2.5079908	1.9994142
C	-0.4734966	-3.5490136	2.4829471
O	-2.0556658	0.1185887	0.2767680
C	-3.1312775	-0.7982462	0.4906377
C	-3.1756846	-1.8179623	-0.6257919
O	-1.9318815	-2.5132219	-0.6780875
C	-1.9189678	-3.5457097	-1.6628664
C	-3.5502620	2.6151381	-0.2120370
Si	0.4509909	1.7775940	-2.1954118
C	1.9552292	2.6222084	-2.9682313
O	0.7993629	0.1206176	-1.9140020
C	1.1752288	-0.7877958	-2.9518071
C	2.1442742	-1.8186982	-2.4164553
O	1.5346456	-2.5231124	-1.3373472
C	2.3505278	-3.5817581	-0.8386304
C	-1.0178699	1.9085923	-3.3779961
Na	-0.0031938	-1.1891709	-0.0067992
H	-2.6500504	-4.3243273	-1.4216374
H	-0.9181730	-3.9721040	-1.6688897
H	-2.1442718	-3.1434969	-2.6569563
H	3.3265431	-3.2058383	-0.5120449
H	2.5047677	-4.3491978	-1.6043823
H	1.8316477	-4.0143883	0.0139408
H	0.1117163	-4.3310861	2.9779424
H	-0.9905456	-3.9684614	1.6225125
H	-1.2124900	-3.1579767	3.1914773
H	1.6616247	-0.2691717	-3.7849933
H	0.2807796	-1.2848135	-3.3422009
H	2.4067504	-2.5148455	-3.2235133

H	3.0639211	-1.3313763	-2.0683576
H	0.6029251	2.4598005	3.6574825
H	1.7665013	3.6634414	3.1005499
H	2.3386609	2.1923532	3.8767160
H	4.1727651	1.5550842	1.5378263
H	3.6902540	2.9946650	0.6470273
H	3.5691251	1.4058347	-0.1183856
H	-3.4587896	2.5062567	-1.2939399
H	-3.5581668	3.6843051	0.0157280
H	-4.5217471	2.2151143	0.0911728
H	-0.7567503	1.5287905	-4.3697900
H	-1.3224881	2.9511838	-3.4998470
H	-1.8789429	1.3449246	-3.0143915
H	-4.0989870	-0.2857708	0.5181907
H	-2.9922256	-1.3041932	1.4521419
H	2.5173852	-0.2969935	3.2666885
H	2.7605526	-1.3199344	1.8429390
H	-3.9961117	-2.5220764	-0.4357609
H	-3.3630907	-1.3212778	-1.5863865
H	2.8508387	2.5074401	-2.3554326
H	1.7614694	3.6926642	-3.0766015
H	2.1717919	2.2324273	-3.9666550
H	1.6248193	-2.5276061	3.6590196
H	0.3216852	-1.3161185	3.6935872
H	-3.4155982	1.5239788	2.8408450
H	-2.3969150	2.9588846	2.8908791
H	-1.6798892	1.3629407	3.1434839
H	0.0001146	4.0440244	0.0032249

[K($MeOCH_2CH_2OSiMe_2$)$_3$Si] (K-**2**)

C	1.7457347	2.7964673	3.1125845
Si	1.7026038	1.9806623	1.3868349
C	3.4546096	2.1913244	0.6541531
Si	0.0289839	2.9265519	0.0356035
Si	-2.0024788	2.0216524	0.7935656
C	-2.2481659	2.2102600	2.6790895
O	1.5435046	0.2701307	1.7069144
C	2.4898740	-0.4951517	2.4242160
C	1.8147466	-1.6386865	3.1534473
O	1.2048809	-2.5301515	2.2182152
C	0.5821076	-3.6357551	2.8518078
O	-2.2433674	0.3215799	0.4680311
C	-3.3699371	-0.4143352	0.8986195
C	-3.6644370	-1.5522504	-0.0563807
O	-2.5716112	-2.4720730	-0.0821763
C	-2.8211849	-3.5852200	-0.9241224
C	-3.4925428	2.8899076	-0.0263433
Si	0.3640345	2.0287401	-2.1096455
C	1.8311528	2.8760684	-2.9904342
O	0.7340118	0.3215780	-2.1681349
C	0.8957252	-0.4091620	-3.3663273
C	1.8300171	-1.5832512	-3.1584715
O	1.2767792	-2.4935665	-2.2055277
C	2.0888587	-3.6407919	-2.0174937
C	-1.1451092	2.2512265	-3.2601794
K	-0.0106977	-1.1444369	-0.0062832
H	-3.6885712	-4.1603050	-0.5761034

H	-1.9391120	-4.2244477	-0.8952874
H	-2.9997272	-3.2729923	-1.9606552
H	3.0890198	-3.3698966	-1.6570568
H	2.1933106	-4.2110824	-2.9489869
H	1.6033291	-4.2698203	-1.2714529
H	1.3112980	-4.2371456	3.4091987
H	0.1368567	-4.2541412	2.0724968
H	-0.2063799	-3.3109802	3.5418023
H	1.3261334	0.2065195	-4.1675762
H	-0.0736824	-0.7802403	-3.7253318
H	1.9778699	-2.1000754	-4.1178397
H	2.8049918	-1.2257610	-2.8026164
H	0.7889288	2.6911981	3.6284325
H	1.9459161	3.8654520	3.0051223
H	2.5269416	2.3764183	3.7541523
H	4.2298709	1.8642686	1.3542638
H	3.6424746	3.2424548	0.4218701
H	3.5698043	1.6207209	-0.2703312
H	-3.4576669	2.8007461	-1.1142898
H	-3.4718511	3.9551108	0.2165874
H	-4.4523899	2.4915915	0.3178715
H	-0.9179764	1.9487617	-4.2872822
H	-1.4495962	3.3002488	-3.2844651
H	-1.9999146	1.6646989	-2.9149874
H	-4.2733368	0.2080064	0.9469863
H	-3.2015425	-0.8231486	1.9043859
H	3.0143591	0.1038983	3.1800508
H	3.2516457	-0.9009145	1.7443226
H	-4.5755472	-2.0734181	0.2718639

H	-3.8361280	-1.1560290	-1.0657670
H	2.7575647	2.7634715	-2.4230068
H	1.6302461	3.9463568	-3.0815144
H	1.9992983	2.4844408	-3.9989749
H	2.5644424	-2.1828838	3.7460409
H	1.0513871	-1.2445517	3.8372019
H	-3.2511892	1.9022120	2.9903168
H	-2.1172295	3.2549039	2.9714406
H	-1.5219408	1.6150362	3.2375560

$[K(MeOCH_2CH_2OSiMe_2)_3SiH]^+$ [K-**2**-H]$^+$

C	3.0493185	2.7460328	1.7913396
Si	2.2699892	1.7809809	0.3645641
C	3.3441419	1.9644440	-1.1812061
Si	0.0099417	2.4264661	-0.0008078
Si	-1.4376496	1.7872518	1.7755081
C	-0.6250616	1.9406566	3.4764030
O	2.1680874	0.1228746	0.7824006
C	3.3238415	-0.6844002	1.0176147
C	3.0450685	-1.7504567	2.0540446
O	2.0367993	-2.6429528	1.5830995
C	1.8567196	-3.7648809	2.4441126
O	-1.7769169	0.1372127	1.4639251
C	-2.5899718	-0.6567808	2.3315176
C	-3.3150003	-1.7386089	1.5621981
O	-2.3774080	-2.6419364	0.9805175
C	-3.0080685	-3.7656714	0.3709418
C	-3.0467543	2.7792044	1.7547422

Si	-0.8104229	1.7740497	-2.1354540
C	0.0373039	2.7252805	-3.5321577
O	-0.4084906	0.1126815	-2.2483643
C	-0.7914005	-0.6949536	-3.3639339
C	0.2350398	-1.7717163	-3.6369860
O	0.3194975	-2.6653241	-2.5288221
C	1.1427557	-3.7958634	-2.8039863
C	-2.6857207	1.9679974	-2.2881484
K	0.0026504	-1.5148434	0.0160228
H	-3.5694628	-4.3484495	1.1090328
H	-2.2235359	-4.3918509	-0.0521939
H	-3.6889693	-3.4542172	-0.4291143
H	2.1665950	-3.4916072	-3.0492226
H	0.7380676	-4.3832002	-3.6352457
H	1.1599183	-4.4143018	-1.9077467
H	2.7737289	-4.3599183	2.5104059
H	1.0644318	-4.3799727	2.0192271
H	1.5633934	-3.4490476	3.4514576
H	-0.8883195	-0.0954568	-4.2763886
H	-1.7642236	-1.1585509	-3.1655678
H	-0.0710611	-2.3216129	-4.5371937
H	1.2170987	-1.3203837	-3.8283829
H	2.4962460	2.6154049	2.7230648
H	3.0622046	3.8135315	1.5567120
H	4.0850713	2.4408488	1.9644149
H	4.3784549	1.6729984	-0.9788250
H	3.3597223	3.0047918	-1.5157902
H	2.9730773	1.3509499	-2.0045520
H	-3.5841450	2.6667789	0.8116312

H	-2.8303268	3.8417086	1.8923143
H	-3.7148078	2.4777128	2.5660682
H	-3.0341468	1.6673263	-3.2800994
H	-2.9751976	3.0121674	-2.1451879
H	-3.2144643	1.3669390	-1.5456563
H	-3.3495217	-0.0471343	2.8341036
H	-1.9642972	-1.1150796	3.1053659
H	4.1635520	-0.0826109	1.3844248
H	3.6378458	-1.1577132	0.0808514
H	-3.9707733	-2.2787034	2.2582910
H	-3.9406433	-1.2929321	0.7780771
H	1.1205492	2.5929759	-3.5171668
H	-0.1708976	3.7940299	-3.4358138
H	-0.3302951	2.4119802	-4.5132580
H	3.9756083	-2.3034620	2.2405103
H	2.7247311	-1.2890324	2.9970017
H	-1.3160412	1.6458552	4.2712293
H	-0.3317073	2.9755698	3.6703236
H	0.2674199	1.3166607	3.5529166
H	0.0116765	3.9261285	-0.0043645

[Li($MeOCH_2CH_2OSiMe_2$)$_3$Ge] (Li-**3**)

C	3.2586258	2.1932548	1.3962541
Si	2.0638961	1.5253312	0.0619158
C	3.0847571	1.5039083	-1.5523606
Ge	0.0067531	2.7991324	-0.0022651
Si	-1.0871197	1.5426905	1.7534421
C	-0.2022147	1.5248268	3.4462457

O	1.7977817	-0.1627963	0.4614097
C	2.8424580	-1.0525628	0.8190755
C	2.3598605	-1.9966095	1.8991818
O	1.1996719	-2.6686369	1.4227988
C	0.6852439	-3.6108145	2.3487288
O	-1.3089406	-0.1459837	1.3293365
C	-2.1396906	-1.0325907	2.0600014
C	-2.8234887	-1.9886105	1.1070396
O	-1.8240252	-2.6563204	0.3452571
C	-2.3600002	-3.6133900	-0.5526826
C	-2.8367187	2.2226236	2.1155484
Si	-0.9757876	1.5402965	-1.8211167
C	-0.4069957	2.2070491	-3.5198917
O	-0.5098713	-0.1513146	-1.7931436
C	-0.7182906	-1.0380947	-2.8797417
C	0.4577643	-1.9836508	-2.9985066
O	0.6170888	-2.6602247	-1.7571085
C	1.6664968	-3.6131586	-1.7743754
C	-2.8842032	1.5368928	-1.9044842
Li	-0.0099401	-1.0796409	-0.0003387
H	-2.8654342	-4.4228474	-0.0104928
H	-1.5264679	-4.0162890	-1.1233576
H	-3.0800702	-3.1540990	-1.2418625
H	2.6232585	-3.1485174	-2.0438151
H	1.4565587	-4.4177378	-2.4908246
H	1.7392993	-4.0242631	-0.7701031
H	1.4055884	-4.4187620	2.5304427
H	-0.2244912	-4.0192024	1.9148040
H	0.4449062	-3.1366587	3.3087094

H	-0.8200564	-0.5019041	-3.8293320
H	-1.6385822	-1.6130991	-2.7225245
H	0.2734480	-2.7062048	-3.8067418
H	1.3720533	-1.4239709	-3.2366208
H	2.8362964	2.1198265	2.4008187
H	3.4328694	3.2539426	1.1957999
H	4.2348008	1.6986298	1.3939501
H	4.0359538	0.9781988	-1.4209968
H	3.3124482	2.5255300	-1.8680013
H	2.5376161	1.0188767	-2.3631063
H	-3.4903221	2.1589861	1.2429513
H	-2.7431522	3.2809233	2.3737192
H	-3.3320826	1.7265666	2.9559492
H	-3.2462373	1.0159195	-2.7968473
H	-3.2612071	2.5623089	-1.9453214
H	-3.3226624	1.0560736	-1.0279731
H	-2.9149240	-0.4972134	2.6186355
H	-1.5402192	-1.5982114	2.7831473
H	3.7191534	-0.5186962	1.2015025
H	3.1611328	-1.6283244	-0.0579795
H	-3.4208226	-2.7166925	1.6751997
H	-3.4958147	-1.4383248	0.4357348
H	0.6744292	2.1285693	-3.6511980
H	-0.6632321	3.2687225	-3.5711815
H	-0.8950083	1.7154850	-4.3673192
H	3.1504370	-2.7222640	2.1392589
H	2.1158846	-1.4361382	2.8114636
H	-0.7983400	1.0087006	4.2056134
H	-0.0362083	2.5474774	3.7950198

H 0.7699105 1.0320321 3.3838352

$[Li(MeOCH_2CH_2OSiMe_2)_3GeH]^+$ [Li-**3**-H]$^+$

C -2.6678060 -2.1926094 -2.4001666

Si -0.9897077 -1.4205118 -1.9985104

C 0.1052478 -1.4727870 -3.5371181

Ge 0.0012097 -2.3203618 -0.0006957

Si -1.2344398 -1.4318925 1.8612221

C -3.1139127 -1.5064566 1.6859282

O -1.2032303 0.2143339 -1.5088783

C -1.9703379 1.1470209 -2.2719754

C -2.7124874 2.0736740 -1.3366338

O -1.7684567 2.6863785 -0.4606346

C -2.3495492 3.7136935 0.3400290

O -0.7196638 0.2066968 1.7950583

C -0.9900154 1.1403581 2.8413572

C 0.1930380 2.0664804 3.0071315

O 0.4757312 2.6725019 1.7470803

C 1.4510874 3.7093733 1.8434209

C -0.7298315 -2.1948424 3.5153449

Si 2.2329467 -1.4384008 0.1435542

C 3.4168968 -2.2010153 -1.1174861

O 1.9152609 0.1989181 -0.2714812

C 2.9545398 1.1355215 -0.5589718

C 2.5095524 2.0556597 -1.6728918

O 1.2737653 2.6609135 -1.2971313

C 0.8727396 3.6923965 -2.1975563

C 3.0205741 -1.5093516 1.8591828

Li	-0.0056245	1.4157165	0.0016479
H	1.0893228	4.5232163	2.4804886
H	1.6239476	4.0792412	0.8365689
H	2.3905809	3.3281463	2.2584077
H	0.7679656	3.3056030	-3.2173269
H	1.6048017	4.5069513	-2.2043247
H	-0.0874395	4.0635277	-1.8504515
H	-2.7280713	4.5271165	-0.2879514
H	-1.5678273	4.0885353	0.9952753
H	-3.1752952	3.3222612	0.9448142
H	3.8761889	0.6356743	-0.8747545
H	3.1781447	1.7161677	0.3411835
H	3.2756422	2.8240960	-1.8364534
H	2.3780459	1.4959108	-2.6074392
H	-3.3733926	-2.0984419	-1.5729335
H	-2.5352165	-3.2589975	-2.6014698
H	-3.1220112	-1.7530237	-3.2921012
H	-0.3924581	-0.9980091	-4.3875204
H	0.3193579	-2.5065285	-3.8203434
H	1.0564108	-0.9637825	-3.3729476
H	0.3370567	-2.0801575	3.7148153
H	-0.9491869	-3.2658157	3.5003977
H	-1.2854597	-1.7669804	4.3540156
H	4.0080771	-1.0391183	1.8549253
H	3.1544895	-2.5463527	2.1776963
H	2.4068376	-1.0030699	2.6059540
H	-1.1697310	0.6382608	3.7975987
H	-1.8854664	1.7165855	2.5899336
H	-2.7044830	0.6428862	-2.9089779

H	-1.3020104	1.7226527	-2.9190565
H	-0.0482600	2.8340854	3.7531269
H	1.0727952	1.5111618	3.3557104
H	3.0607966	-2.0846252	-2.1425417
H	3.5114338	-3.2723747	-0.9210334
H	4.4211342	-1.7741771	-1.0509865
H	-3.2393159	2.8368331	-1.9237464
H	-3.4543152	1.5169987	-0.7505254
H	-3.6061424	-1.0370967	2.5426444
H	-3.4534383	-2.5445835	1.6438487
H	-3.4546614	-1.0027841	0.7800667
H	-0.0073474	-3.8641339	-0.0063682

$[Na(MeOCH_2CH_2OSiMe_2)_3Ge]$ (Na-**3**)

C	3.1961143	2.3302007	1.5778812
Si	2.1187202	1.5939872	0.1816708
C	3.2024716	1.6593554	-1.3904110
Ge	0.0040869	2.7583262	-0.0038525
Si	-1.2187419	1.6079409	1.7397049
C	-0.3978993	1.6865183	3.4630912
O	1.9652753	-0.1064152	0.5621003
C	3.0439748	-0.9742229	0.8511672
C	2.6126306	-2.0080156	1.8713522
O	1.4998912	-2.7416798	1.3611447
C	1.0198420	-3.7153838	2.2759418
O	-1.4725091	-0.0947778	1.4302674
C	-2.2532603	-0.9594390	2.2316434
C	-2.9312827	-1.9968350	1.3599171

O	-1.9417988	-2.7288038	0.6373132
C	-2.5034041	-3.7133883	-0.2175015
C	-2.9673495	2.3447720	1.9665618
Si	-0.8976516	1.6072677	-1.9329008
C	-0.2208350	2.3459035	-3.5601714
O	-0.4959092	-0.0935787	-1.9937916
C	-0.7780839	-0.9552709	-3.0789162
C	0.3206100	-1.9900410	-3.2122235
O	0.4215191	-2.7330454	-1.9974665
C	1.4479901	-3.7132452	-2.0394025
C	-2.8008114	1.6802064	-2.0891181
Na	-0.0088847	-1.2514085	-0.0040577
H	-3.0183000	-4.4908692	0.3604986
H	-1.6851609	-4.1568194	-0.7820567
H	-3.2179422	-3.2671389	-0.9203547
H	2.4198708	-3.2594078	-2.2688926
H	1.2310720	-4.4793846	-2.7940174
H	1.4987584	-4.1722880	-1.0539923
H	1.7780173	-4.4850214	2.4668406
H	0.1377034	-4.1714198	1.8311182
H	0.7370274	-3.2562571	3.2311659
H	-0.8408866	-0.4129809	-4.0302173
H	-1.7404714	-1.4608442	-2.9235855
H	0.0943200	-2.6674913	-4.0478048
H	1.2774048	-1.4921675	-3.4165221
H	2.7077386	2.2466910	2.5514651
H	3.3511910	3.3944030	1.3810822
H	4.1837991	1.8631580	1.6470206
H	4.1894572	1.2170672	-1.2207867

H	3.3537115	2.6964252	-1.7012238
H	2.7300102	1.1291539	-2.2201691
H	-3.5621117	2.2563195	1.0547768
H	-2.8770292	3.4096067	2.1965911
H	-3.5238532	1.8792122	2.7863328
H	-3.1469069	1.2408674	-3.0304526
H	-3.1412202	2.7186798	-2.0646616
H	-3.2880553	1.1511846	-1.2671711
H	-3.0371829	-0.4198440	2.7766701
H	-1.6215973	-1.4621232	2.9760052
H	3.9056103	-0.4369688	1.2662362
H	3.3811656	-1.4801153	-0.0630632
H	-3.5205857	-2.6811091	1.9870940
H	-3.6091248	-1.5025675	0.6520332
H	0.8668347	2.2625434	-3.6179300
H	-0.4688500	3.4100301	-3.5954555
H	-0.6497785	1.8811085	-4.4536814
H	3.4469468	-2.6917052	2.0842014
H	2.3238577	-1.5095627	2.8056313
H	-1.0372062	1.2501248	4.2374728
H	-0.2036607	2.7253921	3.7419106
H	0.5563874	1.1554693	3.4703583

$[Na(MeOCH_2CH_2OSiMe_2)_3GeH]^+$ [Na-**3**-H]$^+$

C	3.1618686	-2.3508250	1.7072016
Si	1.4609774	-1.5289633	1.7463720
C	0.7299321	-1.6676220	3.4830815
Ge	-0.0003658	-2.3194310	-0.0033348

Si	0.7777916	-1.5267895	-2.1455840
C	2.6497290	-1.6377032	-2.3798839
O	1.6076262	0.1289759	1.3281894
C	2.4142071	1.0555929	2.0589259
C	3.0274562	2.0681405	1.1171381
O	1.9896173	2.7496815	0.4162807
C	2.4843177	3.7941818	-0.4200780
O	0.3195468	0.1254542	-2.0705775
C	0.5436503	1.0500247	-3.1377249
C	-0.5692727	2.0734952	-3.1826111
O	-0.6284468	2.7618392	-1.9353175
C	-1.5996176	3.8073001	-1.9289489
C	-0.0951729	-2.3683799	-3.5944614
Si	-2.2430544	-1.5208818	0.3925829
C	-3.0701131	-2.3690173	1.8643927
O	-1.9460086	0.1283797	0.7633590
C	-2.9810847	1.0465685	1.1239200
C	-2.4498798	2.0736747	2.0991918
O	-1.3559847	2.7670214	1.5035608
C	-0.8628538	3.8268647	2.3210816
C	-3.3764721	-1.6216040	-1.1158603
Na	-0.0026302	1.4188363	-0.0045066
H	-1.3381785	4.5869583	-2.6519779
H	-1.6133408	4.2273519	-0.9255995
H	-2.5958669	3.4198223	-2.1699012
H	-0.5548985	3.4548497	3.3046046
H	-1.6253010	4.6008899	2.4580461
H	0.0014026	4.2494770	1.8134813
H	2.9618157	4.5788210	0.1762416

H	1.6324183	4.2081778	-0.9553142
H	3.2108958	3.4078163	-1.1437704
H	-3.8249579	0.5351693	1.5993940
H	-3.3548683	1.5463306	0.2239388
H	-3.2541078	2.7776654	2.3492036
H	-2.1194321	1.5845898	3.0244803
H	3.6549596	-2.2297729	0.7411322
H	3.0554462	-3.4225634	1.8947426
H	3.8230593	-1.9524792	2.4817032
H	1.4162082	-1.2612853	4.2316399
H	0.5490895	-2.7137543	3.7426907
H	-0.2167376	-1.1305949	3.5631479
H	-1.1801564	-2.2674813	-3.5337557
H	0.1402342	-3.4359297	-3.5951636
H	0.2333233	-1.9658526	-4.5566768
H	-4.3678797	-1.2186321	-0.8898582
H	-3.5112160	-2.6611065	-1.4253585
H	-2.9687939	-1.0665044	-1.9625796
H	0.5755030	0.5443895	-4.1089674
H	1.5056754	1.5520619	-2.9887302
H	3.2293783	0.5513754	2.5889523
H	1.7955737	1.5666487	2.8044712
H	-0.3708481	2.7817618	-3.9973814
H	-1.5302399	1.5814876	-3.3800995
H	-2.4871084	-2.2622037	2.7808308
H	-3.1744862	-3.4377580	1.6596341
H	-4.0742088	-1.9772915	2.0491427
H	3.6241016	2.7823874	1.6995566
H	3.6913912	1.5680111	0.4005547

H	2.9496540	-1.2312371	-3.3501935
H	2.9795317	-2.6790977	-2.3480070
H	3.1849406	-1.0897048	-1.6022563
H	-0.0051048	-3.8662526	-0.0026728

[K($MeOCH_2CH_2OSiMe_2$)$_3$Ge] (K-**3**)

C	3.0230111	2.5127848	1.8790692
Si	2.1598780	1.6564346	0.4056268
C	3.3526401	1.8281223	-1.0781993
Ge	0.0007773	2.6643154	-0.0020034
Si	-1.4309029	1.6606811	1.6673514
C	-0.7488452	1.8721259	3.4400824
O	2.1721257	-0.0443224	0.8059491
C	3.3531736	-0.7923831	1.0149346
C	3.0948510	-1.9559852	1.9493830
O	2.1476353	-2.8554136	1.3699610
C	1.9211673	-3.9979297	2.1788838
O	-1.7536711	-0.0476668	1.4995286
C	-2.5084640	-0.8062560	2.4228158
C	-3.2124474	-1.9532743	1.7269833
O	-2.2553751	-2.8506926	1.1617486
C	-2.8645104	-3.9592660	0.5201249
C	-3.1519678	2.4892760	1.6585651
Si	-0.7304937	1.6569506	-2.0729826
C	0.1555328	2.4625789	-3.5612636
O	-0.4418135	-0.0557984	-2.2567469
C	-0.8731361	-0.8161214	-3.3671267
C	0.0808608	-1.9609062	-3.6375829

O	0.1123096	-2.8520946	-2.5216340
C	0.9576079	-3.9683371	-2.7449288
C	-2.6027428	1.8886634	-2.3798806
K	0.0063116	-1.4744836	0.0099064
H	-3.4504279	-4.5570182	1.2296387
H	-2.0684590	-4.5799456	0.1091466
H	-3.5227459	-3.6382961	-0.2965799
H	1.9918141	-3.6565091	-2.9371480
H	0.6075839	-4.5677139	-3.5948327
H	0.9360131	-4.5838328	-1.8459709
H	2.8428467	-4.5772207	2.3161624
H	1.1850918	-4.6205341	1.6706476
H	1.5305208	-3.7202030	3.1657832
H	-0.9229372	-0.2140130	-4.2839607
H	-1.8788246	-1.2200055	-3.1874772
H	-0.2520857	-2.5052183	-4.5334005
H	1.0893047	-1.5692057	-3.8245977
H	2.4404476	2.4075230	2.7971910
H	3.1185090	3.5816909	1.6722937
H	4.0285421	2.1217800	2.0658139
H	4.3692460	1.5131336	-0.8222436
H	3.4011793	2.8705414	-1.4024960
H	3.0162868	1.2315489	-1.9297851
H	-3.6491107	2.3655199	0.6938973
H	-3.0391317	3.5621337	1.8341520
H	-3.8132911	2.0946447	2.4365997
H	-2.8974713	1.5656049	-3.3832817
H	-2.8680789	2.9440643	-2.2804286
H	-3.1961977	1.3278804	-1.6539308

H	-3.2833324	-0.2025254	2.9130010
H	-1.8594718	-1.2073693	3.2132503
H	4.1474168	-0.1852600	1.4695307
H	3.7400719	-1.1762387	0.0614221
H	-3.8363996	-2.4905754	2.4560065
H	-3.8636464	-1.5629835	0.9338769
H	1.2383377	2.3357875	-3.4944373
H	-0.0492026	3.5360551	-3.5722759
H	-0.1773326	2.0532826	-4.5206043
H	4.0406744	-2.4864007	2.1330241
H	2.7121373	-1.5865175	2.9095497
H	-1.4685195	1.5474205	4.1984761
H	-0.5203777	2.9238312	3.6305569
H	0.1730797	1.3025567	3.5799329

$[K(MeOCH_2CH_2OSiMe_2)_3GeH]^+$ [K-**3**-H]$^+$

C	1.9790045	2.5752216	2.9624143
Si	1.8976409	1.5951007	1.3488119
C	3.5411138	1.7441591	0.4260746
Ge	0.0000934	2.2458153	-0.0029012
Si	-2.1062056	1.5760544	0.9806749
C	-2.1110670	1.7383782	2.8645987
O	1.5941859	-0.0548013	1.6931981
C	2.4993667	-0.8638974	2.4483240
C	1.7595607	-1.9387793	3.2128002
O	1.1148034	-2.8322198	2.3072602
C	0.5237544	-3.9471883	2.9705190
O	-2.2367442	-0.0781603	0.5586255

C	-3.3182331	-0.9063360	0.9916774
C	-3.6478799	-1.9578452	-0.0445044
O	-2.5393828	-2.8367597	-0.2245322
C	-2.8488451	-3.9333621	-1.0809039
C	-3.5637881	2.5350545	0.2546813
Si	0.2232574	1.5815819	-2.3188335
C	1.5897890	2.5485789	-3.1952817
O	0.6590528	-0.0724453	-2.2305263
C	0.8398549	-0.8852747	-3.3926976
C	1.8742037	-1.9625173	-3.1517110
O	1.4315563	-2.8484120	-2.1251669
C	2.2833043	-3.9816299	-1.9760983
C	-1.3951832	1.7485802	-3.2810818
K	0.0152551	-1.6796712	-0.0042820
H	-3.6561025	-4.5435360	-0.6616288
H	-1.9513866	-4.5440132	-1.1689219
H	-3.1459416	-3.5870992	-2.0771912
H	3.3027594	-3.6808079	-1.7095288
H	2.3160421	-4.5720850	-2.8978332
H	1.8726622	-4.5960550	-1.1758577
H	1.2823929	-4.5403128	3.4922160
H	0.0507045	-4.5676540	2.2103769
H	-0.2343784	-3.6242801	3.6928856
H	1.1806234	-0.2890170	-4.2471020
H	-0.1134426	-1.3491609	-3.6685367
H	2.0187473	-2.5188802	-4.0876110
H	2.8342397	-1.5118689	-2.8687156
H	1.0710856	2.4556088	3.5556654
H	2.0997781	3.6396267	2.7455306

H	2.8312504	2.2715509	3.5766169
H	4.3785264	1.4366394	1.0589927
H	3.7214220	2.7808140	0.1306107
H	3.5519268	1.1288824	-0.4757154
H	-3.6340862	2.4124686	-0.8275733
H	-3.4485875	3.6016824	0.4639415
H	-4.5133491	2.2195412	0.6961642
H	-1.2682297	1.4433996	-4.3234765
H	-1.7311536	2.7885914	-3.2855601
H	-2.1882912	1.1401589	-2.8419762
H	-4.2271262	-0.3189007	1.1651208
H	-3.0521070	-1.3941927	1.9360851
H	3.0546791	-0.2647203	3.1787754
H	3.2281264	-1.3287310	1.7748858
H	-4.5218302	-2.5254159	0.3029954
H	-3.9056683	-1.4815892	-0.9992795
H	2.5578563	2.4164876	-2.7085698
H	1.3548442	3.6159418	-3.1886633
H	1.6894294	2.2450925	-4.2411464
H	2.4841807	-2.4895985	3.8272294
H	1.0172089	-1.4847535	3.8822304
H	-3.0713292	1.4278664	3.2859098
H	-1.9478250	2.7782492	3.1588201
H	-1.3266306	1.1317103	3.3215643
H	-0.0067436	3.7970777	-0.0077105

[(MeOCH$_2$CH$_2$OSiMe$_2$)$_3$CH] (**1**-A)

C	0.2122341	3.3292480	2.3880367
Si	-0.3940611	2.1060616	1.0695136
O	0.7397753	2.0281479	-0.1792761
C	1.0141696	3.1153568	-1.0561565
C	2.3277641	2.9212911	-1.7974775
O	2.3039200	1.8990581	-2.7756805
C	2.6659733	0.6054824	-2.3024448
C	-2.0173434	2.8065544	0.3973275
C	-0.5104324	0.3601046	1.8226213
Si	-1.9559233	-0.7067701	1.1895349
C	-1.7962766	-2.5617578	1.5224355
Si	1.1488632	-0.5528601	2.0277336
C	1.1130738	-1.5860663	3.6193145
C	2.6742552	0.5591858	2.1475716
O	1.3114048	-1.5828337	0.7000407
C	2.4864529	-2.3413676	0.4368210
C	2.2231977	-3.4567144	-0.5630776
O	1.9965670	-3.0167070	-1.8886329
C	0.6360970	-2.7374276	-2.2054453
C	-3.5690635	-0.1481240	2.0184228
O	-2.0673486	-0.4451878	-0.4748156
C	-2.9564543	-1.1625607	-1.3235290
C	-3.1480766	-0.4607362	-2.6591811
O	-2.0202567	-0.5023107	-3.5127679
C	-1.1036101	0.5760828	-3.3527225
H	0.0105179	-3.6263397	-2.0481977
H	0.6056245	-2.4744843	-3.2615433

H	0.2383531	-1.9110207	-1.6144217
H	-0.6441464	0.5812533	-2.3630570
H	-1.6036852	1.5392157	-3.5221078
H	-0.3311771	0.4485453	-4.1094835
H	3.6772013	0.6178595	-1.8735096
H	2.6637825	-0.0586730	-3.1651938
H	1.9647618	0.2310305	-1.5549021
H	-3.9484487	-1.2639268	-0.8594961
H	-2.5680475	-2.1708536	-1.5087948
H	-3.9529967	-0.9810497	-3.1885960
H	-3.4700735	0.5757273	-2.4893418
H	1.1844278	3.0526217	2.7983367
H	-0.5004562	3.3844970	3.2161896
H	0.2996765	4.3369567	1.9727488
H	-1.8387987	3.7898107	-0.0474366
H	-2.7489815	2.9457538	1.1969510
H	-2.4526195	2.1582301	-0.3626388
H	0.3040876	-2.3180614	3.6246607
H	0.9883492	-0.9388003	4.4923547
H	2.0534072	-2.1278549	3.7536678
H	-2.6506954	-3.0896292	1.0896353
H	-1.7984598	-2.7719745	2.5946830
H	-0.8829564	-2.9703716	1.0910811
H	2.8693052	-2.8068304	1.3568346
H	3.2732536	-1.6904110	0.0383003
H	1.0856399	4.0617534	-0.5001042
H	0.2077794	3.2185015	-1.7912321
H	3.1205662	-4.0834248	-0.5965320
H	1.3892310	-4.0794549	-0.2114396

H	-3.7953913	0.9014428	1.8259764
H	-3.5107975	-0.2885689	3.1018212
H	-4.4152099	-0.7429566	1.6637550
H	2.5423651	3.8531299	-2.3311698
H	3.1381844	2.7511519	-1.0759008
H	3.5755661	-0.0558806	2.2213954
H	2.6380226	1.1817102	3.0451212
H	2.7698184	1.2097384	1.2787086
H	-0.8018814	0.5664879	2.8647113

[(MeOCH$_2$CH$_2$OSiMe$_2$)$_3$CH] (**1**-B)

C	-0.6160180	0.7761917	-0.8162385
Si	-2.2484204	-0.1678922	-1.0234892
Si	-0.0854548	0.9850491	1.0042499
Si	0.7836767	0.2155769	-1.9722035
C	-0.1781249	-0.6163845	1.9992094
C	-1.0865230	2.3542495	1.8380791
C	-3.2038527	0.4217624	-2.5477878
C	-2.0456113	-2.0465314	-1.0720904
C	2.0806125	1.5639792	-2.2267822
C	0.1982171	-0.2777193	-3.7109928
O	-3.1705825	0.2290338	0.3424336
C	-4.4781735	-0.2652295	0.5916087
O	1.4377106	-1.1477202	-1.2226984
C	2.5734394	-1.8585854	-1.6870674
O	1.5309909	1.4946207	0.9585047
C	2.3072190	1.7550782	2.1161513
C	-5.0011355	0.3735332	1.8672598

O	-6.3026162	-0.1327959	2.1014566
C	-6.8963890	0.3856748	3.2750388
C	2.7989862	-3.0471345	-0.7685394
O	3.9318958	-3.7543455	-1.2398524
C	4.2405388	-4.8895089	-0.4566521
C	3.6623694	2.2839533	1.6772827
O	4.4279690	2.5410596	2.8405306
C	5.7195895	3.0379666	2.5534569
H	0.2262356	-0.4709248	3.0043526
H	0.3983967	-1.4029599	1.5088789
H	-1.2116080	-0.9516055	2.1030817
H	-0.7937557	2.4812940	2.8838979
H	-0.9339045	3.3103608	1.3299170
H	-2.1493094	2.1137037	1.8060660
H	-4.1774125	-0.0716569	-2.6114222
H	-3.3796826	1.4997333	-2.5026804
H	-2.6676943	0.2057138	-3.4734182
H	-3.0205639	-2.5363338	-1.1427628
H	-1.5467555	-2.4109753	-0.1724695
H	-1.4519185	-2.3677260	-1.9294392
H	2.8930956	1.2096791	-2.8671721
H	2.4920007	1.8908644	-1.2734141
H	1.6304072	2.4291561	-2.7224124
H	1.0609263	-0.5645933	-4.3191220
H	-0.4995454	-1.1165735	-3.7150402
H	-0.2814948	0.5653344	-4.2151171
H	-4.4730696	-1.3543905	0.7142192
H	-5.1595861	-0.0229942	-0.2320056
H	2.4276809	-2.2223250	-2.7112603

H	3.4673260	-1.2245397	-1.6792701
H	2.4549053	0.8442210	2.7079140
H	1.8274644	2.5005434	2.7616643
H	-5.0207671	1.4666485	1.7535673
H	-4.3309129	0.1333584	2.7044075
H	-7.8856750	-0.0624620	3.3622170
H	-7.0025651	1.4776278	3.2248944
H	-6.3101135	0.1330907	4.1687519
H	2.9578592	-2.6934940	0.2597586
H	1.9073804	-3.6897765	-0.7707923
H	5.1189297	-5.3589630	-0.8982335
H	4.4676246	-4.6136097	0.5819705
H	3.4140147	-5.6131314	-0.4518411
H	3.5281330	3.2016594	1.0870660
H	4.1602575	1.5411188	1.0380874
H	6.2221775	3.2040761	3.5058097
H	6.3051268	2.3215217	1.9615109
H	5.6734786	3.9881052	2.0040938
H	-0.8499991	1.7988428	-1.1471244

$[(MeOCH_2CH_2OSiMe_2)_3C]^-$ (**1**$^-$-A)

C	2.4747882	2.8445139	1.3552195
Si	1.9416991	1.7493069	-0.1302123
O	3.1796276	2.0068667	-1.3116193
C	3.7130373	3.2622886	-1.6493475
C	4.6999268	3.1500010	-2.8040285
O	4.1162458	2.9356843	-4.0827077
C	3.7115452	1.5940926	-4.3326835

C	0.3990044	2.6359425	-0.8296545
C	1.7449529	-0.0153263	0.2083492
Si	0.1617225	-0.8230479	-0.1201599
C	0.1227730	-2.7115812	0.1717993
Si	3.1659651	-0.9945265	0.7466855
C	2.9780466	-1.9109571	2.4245822
C	4.8095399	-0.0348306	0.9251914
O	3.5024236	-2.2544464	-0.3909632
C	4.4083230	-3.3062986	-0.1746664
C	4.5633616	-4.1657174	-1.4224644
O	5.3274615	-3.5819931	-2.4701548
C	4.6258564	-2.6199505	-3.2500052
C	-1.3488868	-0.1670949	0.8692053
O	-0.2961623	-0.6152127	-1.7765417
C	-1.5633670	-0.9231409	-2.2998392
C	-1.5988579	-0.7245756	-3.8094243
O	-0.9272106	-1.7219246	-4.5682462
C	0.4892398	-1.5844866	-4.6057220
H	3.7278363	-3.0632867	-3.7023206
H	5.3059930	-2.3106837	-4.0456539
H	4.3210331	-1.7551851	-2.6589167
H	0.9386170	-1.6899880	-3.6173523
H	0.7764885	-0.6050801	-5.0128280
H	0.8581062	-2.3659691	-5.2723912
H	4.5610699	0.9044578	-4.2311503
H	3.3547981	1.5655467	-5.3637092
H	2.9226417	1.2694857	-3.6526759
H	-2.3421966	-0.2727189	-1.8679173
H	-1.8547884	-1.9628455	-2.0853563

H	-2.6425212	-0.7651993	-4.1384692
H	-1.1984545	0.2708130	-4.0496058
H	3.4300520	2.5225872	1.7767921
H	1.7231669	2.7635257	2.1458861
H	2.5636821	3.9034682	1.0883539
H	0.6664173	3.6317742	-1.1971908
H	-0.3690688	2.7692915	-0.0631930
H	-0.0349622	2.0688970	-1.6549470
H	2.1335449	-2.6042337	2.4191176
H	2.7929152	-1.1747628	3.2119059
H	3.8757792	-2.4753994	2.7002939
H	-0.7921408	-3.1426082	-0.2468835
H	0.1383208	-2.9517863	1.2381098
H	0.9792014	-3.1958138	-0.3006066
H	4.0632871	-3.9696297	0.6361375
H	5.4051824	-2.9375317	0.1119392
H	4.2571353	3.7066770	-0.7991124
H	2.9281852	3.9773681	-1.9393617
H	5.1005012	-5.0795121	-1.1472718
H	3.5663008	-4.4479103	-1.7899482
H	-1.5316294	0.8935977	0.6813743
H	-1.1512552	-0.2823986	1.9387471
H	-2.2706824	-0.7136570	0.6410476
H	5.2400581	4.0988947	-2.8888928
H	5.4264350	2.3562601	-2.5792524
H	5.6448160	-0.7290135	1.0614124
H	4.7928243	0.6266701	1.7955112
H	5.0046275	0.5720262	0.0395003

[(MeOCH$_2$CH$_2$OSiMe$_2$)$_3$C]$^-$ (**1**$^-$-B)

C	-0.6072186	0.3128240	-0.7557144
Si	-2.3655746	0.3142979	-1.1373876
Si	-0.1215652	0.9672475	0.8676368
Si	0.6951692	-0.3830185	-1.7904281
C	-0.1783532	-0.2939774	2.3027660
C	-1.0903867	2.4996532	1.4830153
C	-3.0810529	1.9458345	-1.8550380
C	-3.0461953	-1.0452302	-2.2998674
C	1.9934810	0.8655153	-2.4394498
C	0.1535102	-1.3296891	-3.3667056
O	-3.2394072	0.0904542	0.3413059
C	-4.6194434	0.2941986	0.4922198
O	1.6034136	-1.5494224	-0.8911533
C	2.8600142	-2.0455464	-1.2666619
O	1.5202393	1.4964817	0.7956233
C	2.2962664	1.7954020	1.9251111
C	-5.0341048	-0.2940327	1.8319456
O	-6.4326068	-0.0911172	2.0197067
C	-6.8955527	-0.6022069	3.2464193
C	3.1946762	-3.2072417	-0.3444190
O	4.4762566	-3.7298765	-0.6896165
C	4.8589226	-4.8095059	0.1269215
C	3.6116725	2.3882625	1.4447312
O	4.4222488	2.7056357	2.5738905
C	5.6700356	3.2449723	2.2108385
H	0.1265305	0.1206697	3.2701711
H	0.4632581	-1.1466874	2.0662383

H	-1.2028185	-0.6635420	2.3946531
H	-0.7177485	2.8445675	2.4533758
H	-0.9970531	3.3236911	0.7705933
H	-2.1505951	2.2629472	1.5958608
H	-4.1647568	1.9134619	-2.0151830
H	-2.8563123	2.7880729	-1.1952891
H	-2.6077074	2.1547556	-2.8195048
H	-4.1410718	-1.0185353	-2.3060660
H	-2.7330901	-2.0369222	-1.9664124
H	-2.7110111	-0.9135293	-3.3307508
H	2.8145389	0.3892631	-2.9871834
H	2.4047754	1.4375094	-1.6063909
H	1.5038902	1.5703199	-3.1187357
H	1.0391502	-1.7140926	-3.8838036
H	-0.4948511	-2.1762978	-3.1352664
H	-0.3748795	-0.6792615	-4.0693152
H	-5.2044958	-0.1936045	-0.3007432
H	-4.8794804	1.3618776	0.4765471
H	2.8773847	-2.4074163	-2.3057721
H	3.6457026	-1.2824742	-1.1756690
H	2.5083921	0.9001685	2.5253824
H	1.8052519	2.5229118	2.5883119
H	-4.4656725	0.1911788	2.6369720
H	-4.7969834	-1.3665839	1.8507375
H	-7.9666026	-0.4013037	3.3044094
H	-6.3986086	-0.1220776	4.1022629
H	-6.7345023	-1.6878557	3.3234086
H	3.1919349	-2.8587910	0.6970247
H	2.4259327	-3.9864991	-0.4420337

H	5.8452325	-5.1402171	-0.2029629
H	4.9191572	-4.5193046	1.1862978
H	4.1575163	-5.6533837	0.0443230
H	3.4127991	3.2913367	0.8512034
H	4.1250764	1.6649283	0.7970209
H	6.2192129	3.4546043	3.1302940
H	6.2564994	2.5425566	1.6005469
H	5.5610599	4.1811444	1.6430543

$[(MeOCH_2CH_2OSiMe_2)_3Si]^-$ (**2**$^-$-A)

C	2.2970855	2.4651264	2.9358272
Si	2.0084997	1.6066614	1.2405265
O	3.5956968	1.5143800	0.5446404
C	4.3259156	2.6434208	0.1315843
C	5.8199390	2.4028313	0.2316666
O	6.2567020	1.5191041	-0.7912361
C	7.6186739	1.1880834	-0.6775575
C	1.0432147	2.8803623	0.1845952
Si	0.8351317	-0.4098328	1.4780043
Si	0.2291686	-0.9544876	-0.7498827
C	0.3032838	-2.8366779	-1.1022652
Si	2.4508870	-2.0034314	2.1223084
C	1.6179610	-3.6475995	2.6224057
C	3.4276357	-1.4204671	3.6708012
O	3.6490570	-2.6224571	1.0485509
C	4.6743792	-1.8450431	0.4784391
C	5.4935908	-2.6802933	-0.4833273
O	4.7629236	-2.9544904	-1.6712250

C	5.4329289	-3.8409233	-2.5307505
C	-1.5697870	-0.4211542	-1.1036880
O	1.0034903	-0.1877831	-2.0772118
C	2.3959505	-0.2064826	-2.3135015
C	2.7142666	0.4626297	-3.6324725
O	2.5307417	1.8718728	-3.5458224
C	2.7374246	2.5203672	-4.7736252
H	6.4030086	-3.4386063	-2.8636750
H	4.7990849	-3.9922746	-3.4054503
H	5.6131605	-4.8155486	-2.0527206
H	2.0327588	2.1727107	-5.5447627
H	3.7605526	2.3682832	-5.1533036
H	2.5802145	3.5881186	-4.6130635
H	8.2641871	2.0778502	-0.7495059
H	7.8614659	0.5120012	-1.4980293
H	7.8386200	0.6824878	0.2739925
H	2.7812256	-1.2333406	-2.3671982
H	2.9408603	0.3032737	-1.5117247
H	3.7607039	0.2448059	-3.8992105
H	2.0631941	0.0521724	-4.4188798
H	2.8531082	1.8132121	3.6141387
H	1.3351148	2.6879610	3.4053879
H	2.8498621	3.4064886	2.8382867
H	1.5210150	3.8660869	0.1886510
H	0.0314573	2.9981287	0.5828024
H	0.9629298	2.5420172	-0.8508896
H	1.0763288	-4.0826079	1.7794679
H	0.9023134	-3.4810744	3.4314976
H	2.3673645	-4.3725962	2.9551420

H	-0.0801928	-3.0527247	-2.1053633
H	-0.3051926	-3.3868739	-0.3785141
H	1.3255427	-3.2155989	-1.0317138
H	5.3696769	-1.4822770	1.2530424
H	4.2849940	-0.9586015	-0.0317046
H	4.1069769	3.5237850	0.7558805
H	4.0789847	2.9071403	-0.9056068
H	6.4178289	-2.1353566	-0.7375153
H	5.7739097	-3.6245034	0.0072083
H	-1.6898280	0.6547928	-0.9545907
H	-2.2599561	-0.9306626	-0.4259069
H	-1.8475572	-0.6570831	-2.1359046
H	6.3493024	3.3661504	0.1338912
H	6.0532652	1.9826786	1.2203740
H	4.0838420	-2.2193466	4.0328374
H	2.7397923	-1.1484923	4.4758434
H	4.0440264	-0.5444779	3.4540154

$[(MeOCH_2CH_2OSiMe_2)_3Si]^-$ (**2**$^-$-B)

Si	-0.6116660	1.6187414	-1.4519933
Si	-2.4332814	0.1085805	-1.3356280
Si	0.0957634	1.5124945	0.8021488
Si	1.0109043	0.2131923	-2.4245616
C	0.1689383	-0.1438259	1.7625535
C	-0.9546980	2.6771200	1.9019305
C	-3.5878474	0.3048234	-2.8565504
C	-2.1877389	-1.7873805	-1.1698619
C	2.6994950	1.1044618	-2.5699440

C	0.5609453	-0.2996118	-4.2225905
O	-3.3803305	0.5591704	0.0370318
C	-4.6791430	0.0826185	0.2935387
O	1.3400054	-1.2689445	-1.5968209
C	2.2527857	-2.2422826	-2.0418535
O	1.7083917	2.1326047	0.8376243
C	2.4127432	2.4229578	2.0177937
C	-4.9124933	0.1283646	1.7951105
O	-6.2363431	-0.3186231	2.0750792
C	-6.5187504	-0.3462557	3.4540757
C	2.2483606	-3.3834011	-1.0368795
O	3.1546087	-4.3917925	-1.4766951
C	3.2098231	-5.4918691	-0.6000085
C	3.8068374	2.8878905	1.6286084
O	4.5370875	3.2053915	2.8107720
C	5.8484984	3.6389439	2.5397028
H	0.6020683	-0.0240001	2.7622195
H	0.7563878	-0.8768209	1.2064420
H	-0.8425999	-0.5415306	1.8825999
H	-0.6575657	2.6491870	2.9558916
H	-0.8882017	3.7090009	1.5470091
H	-2.0013587	2.3693095	1.8316894
H	-4.4819579	-0.3240792	-2.7913415
H	-3.9011052	1.3464365	-2.9629837
H	-3.0518295	0.0282718	-3.7699605
H	-3.1421226	-2.3213754	-1.0915654
H	-1.5795095	-2.0251908	-0.2956700
H	-1.6573607	-2.1743478	-2.0440934
H	3.4741242	0.4644887	-3.0061814

H	3.0278010	1.4362841	-1.5828796
H	2.5988633	1.9914101	-3.2021509
H	1.3751215	-0.8422682	-4.7142718
H	-0.3305676	-0.9321048	-4.2498698
H	0.3502689	0.5947749	-4.8153888
H	-4.8257242	-0.9505091	-0.0511723
H	-5.4348572	0.7039411	-0.2064560
H	1.9792471	-2.6419265	-3.0284118
H	3.2738635	-1.8436416	-2.1215942
H	2.5027175	1.5451169	2.6735558
H	1.9252541	3.2175469	2.5997243
H	-4.7698045	1.1550889	2.1588001
H	-4.1768415	-0.5133759	2.2985316
H	-7.5454389	-0.6963497	3.5730003
H	-6.4299346	0.6511473	3.9086934
H	-5.8457619	-1.0283674	3.9941136
H	2.5454400	-3.0059289	-0.0491767
H	1.2325786	-3.7922974	-0.9507568
H	3.9158797	-6.2108622	-1.0184631
H	3.5546699	-5.1988222	0.4025627
H	2.2292179	-5.9787340	-0.4946234
H	3.7304993	3.7690562	0.9769479
H	4.3160203	2.0939619	1.0653543
H	6.3266940	3.8607713	3.4953580
H	6.4351107	2.8658465	2.0224063
H	5.8578183	4.5475234	1.9195287

[(MeOCH$_2$CH$_2$OSiMe$_2$)$_3$SiH] (**2**-H-A)

C	2.0068629	2.5408007	2.9242255
Si	2.0021877	1.7690655	1.1881720
O	3.6077939	1.5931002	0.6635035
C	4.3868554	2.6386239	0.0998598
C	5.8489690	2.2521142	0.0587888
O	6.0598847	1.2328971	-0.9034849
C	7.4137877	0.8405620	-0.9936501
C	1.0477258	2.9148796	0.0218237
Si	1.1526111	-0.4432635	1.1909110
Si	0.4383245	-1.1267286	-0.9926972
C	0.7967764	-2.9606234	-1.2945264
Si	2.5631585	-2.0116158	2.3064548
C	1.5548384	-3.4932854	2.9048162
C	3.4669573	-1.2141301	3.7727666
O	3.6891268	-2.6974835	1.2541305
C	4.7673765	-2.0343706	0.6152157
C	5.3800097	-2.9428756	-0.4280090
O	4.5013545	-3.0856759	-1.5323453
C	4.9642653	-4.0221642	-2.4839975
C	-1.4039687	-0.7697769	-1.2111257
O	1.1732152	-0.1886654	-2.1855899
C	2.5705900	-0.0970423	-2.4323839
C	2.8307040	0.8607777	-3.5720376
O	2.5742249	2.1949289	-3.1575811
C	2.6828628	3.1268986	-4.2127646
H	5.9321051	-3.7225023	-2.9102438
H	4.2257729	-4.0657935	-3.2840961

H	5.0716940	-5.0230673	-2.0453030
H	1.9648917	2.9120077	-5.0156950
H	3.6940680	3.1367671	-4.6434625
H	2.4650262	4.1123189	-3.8009759
H	8.0602084	1.6796037	-1.2872052
H	7.4772931	0.0650283	-1.7565306
H	7.7836697	0.4361032	-0.0413193
H	2.9840578	-1.0761299	-2.7001498
H	3.1047460	0.2548997	-1.5431182
H	3.8800741	0.7616000	-3.8877758
H	2.1863038	0.5994717	-4.4232562
H	2.5758259	1.9358288	3.6326154
H	0.9858926	2.6349274	3.3041053
H	2.4463862	3.5426621	2.9040517
H	1.4248100	3.9403654	0.0812411
H	-0.0089755	2.9371585	0.3022511
H	1.1242886	2.5788025	-1.0137390
H	1.0765596	-4.0049360	2.0675530
H	0.7758817	-3.1832871	3.6054779
H	2.2065504	-4.2112690	3.4099857
H	0.4687023	-3.2464214	-2.2984330
H	0.2538732	-3.5804271	-0.5753476
H	1.8609454	-3.1840144	-1.1956005
H	5.5498845	-1.7896516	1.3460032
H	4.4507357	-1.0956560	0.1484810
H	4.3084342	3.5575888	0.6976676
H	4.0421182	2.8597107	-0.9160165
H	6.3366975	-2.5112816	-0.7603285
H	5.5850894	-3.9241587	0.0228394

H	-1.6182596	0.2911369	-1.0700779
H	-2.0009119	-1.3368023	-0.4927374
H	-1.7212338	-1.0491269	-2.2193379
H	6.4399718	3.1445432	-0.2024186
H	6.1701439	1.9115371	1.0535612
H	4.0924623	-1.9526697	4.2827808
H	2.7508782	-0.8203457	4.4989480
H	4.1054575	-0.3875172	3.4532656
H	-0.0943122	-0.3894834	2.0329831

[$(MeOCH_2CH_2OSiMe_2)_3SiH$] (**2**-H-B)

Si	-0.4691057	1.3470298	-0.1088820
Si	-2.3353928	0.5601406	-1.3414318
Si	-0.0358952	-0.0566258	1.7484379
Si	1.4579823	1.6357675	-1.4535843
C	0.0212445	-1.8822809	1.2445229
C	-1.3337307	0.1818351	3.1073490
C	-3.1826491	1.9613804	-2.2992939
C	-1.8881970	-0.8317697	-2.5476018
C	2.8653002	2.4549980	-0.4883380
C	1.1199712	2.6550633	-3.0203005
O	-3.3922818	-0.0374227	-0.1578739
C	-4.6839488	-0.5648352	-0.4312624
O	1.8814911	0.0558122	-1.8905571
C	3.0261669	-0.2872342	-2.6589089
O	1.4868768	0.4263718	2.3148261
C	2.1053464	-0.1084940	3.4768584
C	-5.3573485	-0.8850206	0.8919479

O	-6.6400296	-1.4143967	0.6097698
C	-7.3730274	-1.7398313	1.7737955
C	3.0610184	-1.7984835	-2.8114173
O	4.2046978	-2.1314517	-3.5775278
C	4.3450738	-3.5222794	-3.7880851
C	3.4826830	0.5174043	3.6151241
O	4.0885018	-0.0173939	4.7783229
C	5.3856326	0.4935980	5.0115549
H	0.2894672	-2.5215341	2.0904618
H	0.7490856	-2.0452638	0.4470528
H	-0.9593253	-2.2037301	0.8845126
H	-1.1690898	-0.4992731	3.9471097
H	-1.3223782	1.2051180	3.4890290
H	-2.3280792	-0.0157309	2.6999054
H	-4.0464665	1.5981562	-2.8633669
H	-3.5222117	2.7485796	-1.6228328
H	-2.4891784	2.4114755	-3.0145709
H	-2.7711553	-1.1824280	-3.0893321
H	-1.4469177	-1.6827997	-2.0250483
H	-1.1592445	-0.4841092	-3.2835468
H	3.7758518	2.5295450	-1.0897049
H	3.0862563	1.8908827	0.4193148
H	2.5792576	3.4678893	-0.1924951
H	2.0310758	2.7857656	-3.6115560
H	0.3712127	2.1797101	-3.6579987
H	0.7540143	3.6512017	-2.7572954
H	-4.6181368	-1.4795299	-1.0317674
H	-5.3006480	0.1559387	-0.9802555
H	2.9899766	0.1726923	-3.6533521

H	3.9480670	0.0443152	-2.1675051
H	2.2137829	-1.1971967	3.4064231
H	1.5183715	0.1141729	4.3751996
H	-5.4340804	0.0291870	1.4971666
H	-4.7491349	-1.6099745	1.4510216
H	-8.3368012	-2.1318627	1.4506368
H	-7.5409768	-0.8565157	2.4047701
H	-6.8599203	-2.5039885	2.3731625
H	3.1035567	-2.2684972	-1.8190390
H	2.1426268	-2.1413879	-3.3081813
H	5.2462749	-3.6704517	-4.3821942
H	4.4497437	-4.0652666	-2.8391596
H	3.4862843	-3.9363016	-4.3334407
H	3.3859673	1.6095555	3.6896100
H	4.0830334	0.2914645	2.7225953
H	5.7610797	0.0226794	5.9194815
H	6.0661394	0.2606798	4.1812993
H	5.3706859	1.5825600	5.1548297
H	-0.8404886	2.7038217	0.4170616

$[(MeOCH_2CH_2OSiMe_2)_3Ge]^-$ (**3**$^-$-A)

C	1.4428250	1.6836405	3.8287809
Si	0.3014431	1.4786265	2.3120572
O	1.3021590	2.1577074	1.0947906
C	0.9803141	2.1330018	-0.2825560
C	1.5152615	3.3596170	-0.9874598
O	2.9359336	3.3108182	-1.0969728
C	3.4695975	4.4720797	-1.6771613

C	-1.1267826	2.7362131	2.5607670
Ge	-0.5093274	-0.8134648	2.1962064
Si	-2.3966514	-0.7224141	0.6736628
C	-2.9244772	-2.5028622	0.1748216
Si	1.2265033	-1.8510448	0.8789164
C	1.1012023	-3.7693111	0.9706977
C	2.9791557	-1.4478237	1.5465218
O	1.2769484	-1.4900256	-0.8141256
C	2.2356850	-1.9975887	-1.7091288
C	1.6528507	-2.9848551	-2.7064346
O	0.8633107	-2.3070028	-3.6744192
C	0.1960623	-3.1876819	-4.5458877
C	-3.9139349	0.0381551	1.5499967
O	-2.4328613	0.1815516	-0.7912742
C	-1.6575923	-0.1325949	-1.9263538
C	-2.0025886	0.7936555	-3.0725956
O	-1.5087650	2.1077458	-2.8391845
C	-1.9207352	3.0233683	-3.8218272
H	0.9013119	-3.8156465	-5.1127702
H	-0.3758441	-2.5815998	-5.2494362
H	-0.4955211	-3.8503890	-4.0064543
H	-3.0165424	3.1169766	-3.8590921
H	-1.5675735	2.7377025	-4.8254596
H	-1.4959518	3.9951293	-3.5656518
H	3.0887650	4.6367091	-2.6982779
H	4.5531631	4.3501249	-1.7248094
H	3.2424488	5.3702744	-1.0822154
H	-1.8654736	-1.1553325	-2.2763439
H	-0.5854339	-0.0916858	-1.7132396

H	-1.5603090	0.3956761	-3.9999380
H	-3.0956409	0.8256775	-3.1945280
H	2.3019781	1.0120051	3.7652174
H	0.9003705	1.4482775	4.7486408
H	1.8167080	2.7104585	3.8922074
H	-0.7245859	3.7256457	2.8037026
H	-1.7870624	2.4224860	3.3742910
H	-1.7354543	2.8277151	1.6577786
H	0.1449964	-4.1227164	0.5755223
H	1.1613161	-4.0880167	2.0149442
H	1.9061391	-4.2695359	0.4199177
H	-3.8446199	-2.4855270	-0.4195708
H	-3.1050534	-3.1025375	1.0710648
H	-2.1509997	-3.0101656	-0.4072412
H	3.0571106	-2.5123406	-1.1898978
H	2.6821372	-1.1660339	-2.2692637
H	-0.1023943	2.1144161	-0.4520184
H	1.3927931	1.2340184	-0.7537770
H	2.4769625	-3.5163580	-3.2130226
H	1.0445252	-3.7280285	-2.1736244
H	-3.7327757	1.0834933	1.8090347
H	-4.1338803	-0.5028486	2.4741291
H	-4.7936835	-0.0039728	0.8998688
H	1.0703820	3.4085318	-1.9931590
H	1.2176154	4.2604888	-0.4305260
H	3.7641899	-2.0282595	1.0495456
H	3.0282012	-1.6727552	2.6157220
H	3.2023375	-0.3863803	1.4163518

$[(MeOCH_2CH_2OSiMe_2)_3Ge]^-$ (**3**$^-$-B)

Ge	-0.4716294	0.2694362	-1.5708237
Si	-2.1773915	-1.0883987	-0.4918719
Si	0.1926162	1.5788787	0.3640046
Si	1.3498469	-1.3332728	-1.5282207
C	0.4879420	0.7991562	2.0904909
C	-1.0580574	2.9985490	0.6729193
C	-3.0789851	-2.1784381	-1.7914806
C	-1.8237271	-2.2863390	0.9641413
C	2.9810362	-0.5132815	-2.1118547
C	1.0706146	-2.7925522	-2.7493415
O	-3.3629631	0.0179207	0.1042759
C	-4.5981696	-0.3612076	0.6594469
O	1.7132003	-2.0765948	-0.0095088
C	2.7291484	-3.0307514	0.1867140
O	1.6861592	2.3340692	-0.0640176
C	2.3519133	3.2836527	0.7300702
C	-5.3675836	0.9075173	0.9894529
O	-6.6279930	0.5554619	1.5534090
C	-7.4054272	1.6804762	1.8870561
C	2.7585052	-3.3823465	1.6654757
O	3.7736662	-4.3571551	1.8914264
C	3.8729562	-4.7328906	3.2445993
C	3.4678456	3.8888764	-0.1072278
O	4.1782578	4.8413072	0.6800166
C	5.2325167	5.4512381	-0.0253189
H	0.8949622	1.5229580	2.8063623
H	1.1706100	-0.0493818	2.0151262

H	-0.4588965	0.4303126	2.4944594
H	-0.7944602	3.6241592	1.5325944
H	-1.1401047	3.6372410	-0.2105388
H	-2.0434698	2.5615446	0.8560074
H	-3.9132433	-2.7450380	-1.3635832
H	-3.4601803	-1.5629180	-2.6103845
H	-2.3771887	-2.8985198	-2.2230111
H	-2.7157957	-2.8504511	1.2599385
H	-1.4512586	-1.7440526	1.8358104
H	-1.0463756	-2.9968072	0.6739324
H	3.8235824	-1.2132438	-2.1312684
H	3.2331927	0.3265691	-1.4607199
H	2.8498892	-0.1176222	-3.1231214
H	1.9573086	-3.4281352	-2.8476021
H	0.2351773	-3.4239543	-2.4347971
H	0.8303120	-2.3985375	-3.7407526
H	-4.4718955	-0.9503104	1.5786835
H	-5.1946997	-0.9656688	-0.0378972
H	2.5479857	-3.9472472	-0.3922067
H	3.7163245	-2.6483162	-0.1067728
H	2.7890425	2.8292251	1.6300821
H	1.6824242	4.0894377	1.0622768
H	-5.5084738	1.4987762	0.0742806
H	-4.7876392	1.5152125	1.6970766
H	-8.3469383	1.3189163	2.3034443
H	-7.6230812	2.3014906	1.0056041
H	-6.9069487	2.3136310	2.6357206
H	2.9589415	-2.4769583	2.2542932
H	1.7781152	-3.7719509	1.9703635

H	4.6668474	-5.4774022	3.3225539
H	4.1256404	-3.8773935	3.8880392
H	2.9360580	-5.1738857	3.6152005
H	3.0391211	4.3697194	-0.9968966
H	4.1437109	3.0925689	-0.4470084
H	5.7184343	6.1554046	0.6519924
H	5.9764288	4.7153085	-0.3639625
H	4.8698710	6.0008376	-0.9065895

[$(MeOCH_2CH_2OSiMe_2)_3GeH$] (**3**-H-A)

C	2.8153223	2.8062408	1.5650198
Si	2.2691412	1.9067028	-0.0026074
O	3.6411064	1.9258659	-0.9799700
C	3.7525245	1.3407611	-2.2712782
C	5.1398262	1.5676720	-2.8255433
O	6.0854929	0.7760493	-2.1207911
C	7.4147269	1.0201060	-2.5303499
C	0.8524577	2.8342759	-0.8469467
Ge	1.6319419	-0.3875326	0.5154923
Si	-0.3982803	-1.1546738	-0.5747009
C	-0.5085593	-3.0486886	-0.5611473
Si	3.4877084	-1.9098788	0.2755129
C	3.2207722	-3.5278901	1.2323592
C	5.1311106	-1.1434761	0.8182739
O	3.5167261	-2.2558484	-1.3860500
C	4.6612165	-2.7068409	-2.0959070
C	4.2794893	-3.1589306	-3.4879773
O	3.9155700	-2.0435234	-4.2836347

C	3.5594555	-2.4079324	-5.6008909
C	-1.9184957	-0.4073842	0.2594300
O	-0.4953248	-0.5952673	-2.1629135
C	0.3560136	-0.9584567	-3.2370908
C	0.0965232	-0.0566243	-4.4236088
O	0.5973110	1.2458685	-4.1687409
C	0.2618401	2.1713157	-5.1826238
H	4.3904235	-2.9029282	-6.1226894
H	3.3052267	-1.4925298	-6.1345222
H	2.6917746	-3.0819304	-5.6153567
H	-0.8260608	2.2769594	-5.2876442
H	0.6786215	1.8756340	-6.1556965
H	0.6834193	3.1344987	-4.8964801
H	7.5610980	0.7865632	-3.5944693
H	8.0628426	0.3766552	-1.9355916
H	7.7029637	2.0664430	-2.3614582
H	0.1532620	-1.9914880	-3.5504375
H	1.4131719	-0.9037673	-2.9562126
H	0.5886178	-0.4822614	-5.3118071
H	-0.9845291	-0.0165800	-4.6168716
H	3.6534073	2.2922836	2.0390694
H	1.9983306	2.8665040	2.2880799
H	3.1350539	3.8231462	1.3214370
H	1.1650965	3.8547154	-1.0889374
H	-0.0136065	2.8970430	-0.1820978
H	0.5361379	2.3372858	-1.7664087
H	2.3032142	-4.0311454	0.9212989
H	3.1510720	-3.3315856	2.3056193
H	4.0558964	-4.2170689	1.0739733

H	-1.4274999	-3.3829114	-1.0519516
H	-0.5176196	-3.4261044	0.4648003
H	0.3406014	-3.5041316	-1.0753518
H	5.1279994	-3.5632928	-1.5893310
H	5.4029979	-1.9040455	-2.1689619
H	3.0270177	1.7830343	-2.9635393
H	3.5574482	0.2639866	-2.2323437
H	5.1421794	-3.6759155	-3.9375136
H	3.4469639	-3.8750391	-3.4290095
H	-1.8954686	0.6826591	0.2071869
H	-1.9694969	-0.6972645	1.3115605
H	-2.8297948	-0.7507871	-0.2376985
H	5.1435767	1.2949623	-3.8914595
H	5.3998272	2.6322063	-2.7391992
H	5.9498846	-1.8648587	0.7349752
H	5.0706391	-0.8344299	1.8654106
H	5.3800384	-0.2689503	0.2138935
H	1.2838393	-0.3987594	2.0355944

[$(MeOCH_2CH_2OSiMe_2)_3GeH$] (**3**-H-B)

Ge	-0.6877535	0.9937979	-0.9909959
Si	-2.7226528	-0.2934269	-1.1319452
Si	0.0251379	1.2092012	1.3015209
Si	1.1105422	0.1785517	-2.3685274
C	-0.0286681	-0.4598201	2.1950733
C	-1.0369479	2.4642197	2.2408896
C	-3.7489978	0.1721362	-2.6570460
C	-2.4039313	-2.1616977	-1.1451777

C	2.5231672	1.4368134	-2.4348461
C	0.5741606	-0.2429492	-4.1400035
O	-3.5648023	0.1284648	0.2769323
C	-4.8682181	-0.3366520	0.6037476
O	1.6070665	-1.2327131	-1.5790215
C	2.7465511	-1.9996761	-1.9414645
O	1.6256779	1.7564298	1.2108904
C	2.4493914	1.9820087	2.3465943
C	-5.2936918	0.3146037	1.9090696
O	-6.5889129	-0.1610776	2.2277756
C	-7.0997553	0.3798549	3.4299042
C	2.8964927	-3.1279694	-0.9360079
O	4.0387292	-3.8838966	-1.2962601
C	4.2848475	-4.9655469	-0.4204465
C	3.8122313	2.4462252	1.8630022
O	4.6272163	2.6609046	3.0011893
C	5.9308246	3.0979744	2.6733166
H	0.3356507	-0.3748078	3.2226443
H	0.5808609	-1.2008443	1.6737656
H	-1.0563893	-0.8288848	2.2352975
H	-0.7408866	2.5373606	3.2913527
H	-0.9634707	3.4577377	1.7935359
H	-2.0838752	2.1539385	2.2047062
H	-4.6760875	-0.4057649	-2.7094949
H	-4.0068291	1.2333213	-2.6502221
H	-3.1829503	-0.0284846	-3.5705565
H	-3.3418080	-2.7242646	-1.1654733
H	-1.8370962	-2.4730392	-0.2655199
H	-1.8266452	-2.4453475	-2.0287646

H	3.3764076	1.0610307	-3.0066451
H	2.8564969	1.6880070	-1.4264562
H	2.1814857	2.3578299	-2.9145428
H	1.4212514	-0.5953753	-4.7358217
H	-0.1927516	-1.0207331	-4.1537045
H	0.1667234	0.6410530	-4.6375653
H	-4.8812785	-1.4258904	0.7263424
H	-5.5913728	-0.0762322	-0.1775883
H	2.6365415	-2.4254211	-2.9458909
H	3.6565892	-1.3890830	-1.9313025
H	2.5735529	1.0658877	2.9356549
H	2.0216225	2.7492798	3.0026549
H	-5.2939850	1.4075042	1.7934560
H	-4.5760582	0.0589558	2.7014612
H	-8.0925050	-0.0437820	3.5788303
H	-7.1834958	1.4736084	3.3768391
H	-6.4682586	0.1198920	4.2900863
H	3.0063481	-2.7078177	0.0734693
H	1.9931858	-3.7537935	-0.9447634
H	5.1761240	-5.4780894	-0.7811653
H	4.4630512	-4.6201244	0.6068503
H	3.4455560	-5.6741043	-0.4088585
H	3.7027845	3.3705600	1.2787420
H	4.2506248	1.6814577	1.2061401
H	6.4708564	3.2372317	3.6093360
H	6.4629007	2.3569733	2.0614604
H	5.9112364	4.0509076	2.1274975
H	-1.0344112	2.4150872	-1.5159568

[(SiMe$_3$)$_3$C]$^-$ (**7**$^-$)

C	2.2562551	2.7725955	1.4373531
Si	1.9693161	1.7580082	-0.1818432
C	3.3997730	2.3264087	-1.3439781
C	0.4491840	2.6442820	-0.9662173
C	1.7914155	-0.0455709	0.0457084
Si	0.1564246	-0.8293924	-0.1744601
C	0.1215141	-2.7435154	0.0432032
Si	3.1756377	-1.0170325	0.7358430
C	2.9082873	-1.6865568	2.5287145
C	4.8499993	-0.0766078	0.8878525
C	3.6939836	-2.5779427	-0.2715998
C	-1.2122378	-0.2405174	1.0558232
C	-0.6534423	-0.6191800	-1.9121505
H	3.1404073	2.4255225	1.9791792
H	1.3989623	2.6542511	2.1071394
H	2.3855809	3.8425976	1.2345214
H	0.6237235	3.7255067	-0.9517595
H	-0.4831789	2.4535058	-0.4292600
H	0.3027759	2.3466452	-2.0075506
H	2.0063737	-2.3000089	2.6025011
H	2.7888188	-0.8501162	3.2243290
H	3.7556283	-2.2940834	2.8689047
H	-0.9178837	-3.0858849	-0.0069774
H	0.5325023	-3.0756294	0.9994359
H	0.6745183	-3.2491256	-0.7524370
H	-1.3508806	0.8433606	1.0157660
H	-0.9224005	-0.4918639	2.0808930

H	-2.1814814	-0.7109865	0.8508641
H	5.5706474	-0.7196217	1.4045751
H	4.7710276	0.8533618	1.4559509
H	5.2660231	0.1649431	-0.0934192
H	3.3771251	3.4132017	-1.4870061
H	3.2977951	1.8520773	-2.3246357
H	4.3829474	2.0626445	-0.9496956
H	2.8995127	-3.3255402	-0.3194871
H	4.5756318	-3.0546688	0.1728535
H	3.9423875	-2.2939096	-1.2986475
H	-0.8785514	0.4238966	-2.1429131
H	-1.5894716	-1.1862195	-1.9771408
H	0.0239002	-0.9904730	-2.6871372

$[(SiMe_3)_3CH]$ (**7**-H)

C	0.4229467	3.3225047	2.3772272
Si	-0.3716271	2.1274520	1.1284152
C	0.6522427	2.1092534	-0.4732961
C	-2.0444299	2.9319881	0.7008160
C	-0.5321154	0.3734616	1.8981868
Si	-1.9938237	-0.6722233	1.2179991
C	-1.9627848	-2.4921404	1.7784564
Si	1.1263236	-0.5800449	2.0817234
C	1.0256312	-1.7894026	3.5467314
C	2.6119474	0.5400824	2.4886992
C	1.5787589	-1.5354367	0.5007507
C	-3.6440666	0.0008005	1.8828052
C	-2.0316326	-0.6894522	-0.6829555

H	1.4536150	3.0677673	2.6229226
H	-0.1484238	3.3428491	3.3096104
H	0.4185406	4.3374656	1.9692048
H	-1.8534301	3.9323080	0.3005417
H	-2.6739148	3.0509988	1.5857628
H	-2.6163571	2.3843260	-0.0497138
H	0.2934545	-2.5831241	3.4002769
H	0.7660755	-1.2598175	4.4679461
H	2.0000966	-2.2605303	3.7045113
H	-2.8473944	-2.9916635	1.3721581
H	-2.0081682	-2.5839611	2.8661720
H	-1.0879793	-3.0430638	1.4296116
H	-3.8670262	1.0114419	1.5426605
H	-3.6447263	0.0072650	2.9765559
H	-4.4634012	-0.6482620	1.5601683
H	3.4962927	-0.0923801	2.6112861
H	2.4665982	1.0811140	3.4267289
H	2.8372020	1.2688803	1.7089010
H	-0.8259388	0.5768538	2.9400060
H	0.1997651	1.4652183	-1.2307103
H	1.6767448	1.7680299	-0.3142246
H	0.7035258	3.1196914	-0.8883272
H	0.8470695	-2.3058624	0.2504832
H	2.5443788	-2.0309885	0.6350884
H	1.6683976	-0.8691445	-0.3603251
H	-2.1732939	0.3045684	-1.1112979
H	-2.8591884	-1.3157540	-1.0279060
H	-1.1101625	-1.1032721	-1.0985961

$[(SiMe_3)_3Si]^-$ (**8**$^-$)

C	2.1538672	2.3852095	3.0547372
Si	1.8588808	1.5903468	1.3252717
C	3.5850108	1.6797568	0.4672308
C	0.7957999	2.8900549	0.3810017
Si	0.6977692	-0.4711762	1.5754598
Si	0.1980861	-1.0360650	-0.6805408
C	-0.2762724	-2.8955167	-0.8514670
Si	2.4674379	-1.9774828	2.0852169
C	1.7439921	-3.6926993	2.5801107
C	3.4482290	-1.4109814	3.6436593
C	3.8381763	-2.4011647	0.7949537
C	-1.3774538	-0.0915295	-1.2596774
C	1.4690231	-0.7401654	-2.1021182
H	2.7916255	1.7525630	3.6778550
H	1.2041166	2.5108249	3.5816514
H	2.6322929	3.3678136	2.9678142
H	1.2542280	3.8841279	0.4358446
H	-0.2090528	2.9542811	0.8070060
H	0.6901086	2.6284955	-0.6753464
H	1.1966417	-4.1473610	1.7499212
H	1.0427089	-3.5824541	3.4115589
H	2.5347698	-4.3877816	2.8854778
H	-0.6748475	-3.1067631	-1.8504241
H	-1.0360379	-3.1725283	-0.1157390
H	0.5886310	-3.5453930	-0.6927956
H	-1.2156995	0.9897811	-1.2589819
H	-2.2133436	-0.2967141	-0.5856082

H	-1.6705770	-0.3896487	-2.2730753
H	4.1373287	-2.1958244	3.9759948
H	2.7692929	-1.1815113	4.4693009
H	4.0374695	-0.5124646	3.4413857
H	3.5357408	1.3010773	-0.5567066
H	4.3225695	1.0766518	1.0039530
H	3.9564448	2.7113237	0.4328214
H	3.4084076	-2.8657353	-0.0965607
H	4.5741719	-3.0978649	1.2150189
H	4.3668186	-1.5008349	0.4725884
H	1.7179858	0.3207693	-2.1911326
H	1.0674328	-1.0732974	-3.0670827
H	2.4007680	-1.2802826	-1.9172399

[$(SiMe_3)_3SiH$] (**8**-H)

C	1.8794022	2.4418123	3.0364090
Si	1.8884548	1.7722272	1.2539407
C	3.6615246	1.8822229	0.5655535
C	0.7665661	2.8929170	0.1980925
Si	1.1084808	-0.4799907	1.2340187
Si	0.3678273	-1.1896790	-0.9175527
C	0.0119035	-3.0612604	-0.9350679
Si	2.6387708	-2.0049906	2.2402126
C	1.7331435	-3.6130538	2.7088266
C	3.3897038	-1.2704850	3.8297805
C	4.0601985	-2.4255044	1.0433997
C	-1.2416806	-0.2813431	-1.3759695
C	1.6824240	-0.8186645	-2.2456129

H	2.5295244	1.8585737	3.6917794
H	0.8727980	2.4203243	3.4608572
H	2.2282608	3.4788254	3.0535372
H	1.1013527	3.9324074	0.2661773
H	-0.2709203	2.8535809	0.5378054
H	0.7852967	2.6066704	-0.8558095
H	1.3038724	-4.1073470	1.8349240
H	0.9211320	-3.4153957	3.4124081
H	2.4259261	-4.3140475	3.1844828
H	-0.3858960	-3.3599740	-1.9096880
H	-0.7243833	-3.3353539	-0.1759608
H	0.9157420	-3.6468801	-0.7525940
H	-1.1000926	0.8006545	-1.4164518
H	-2.0304230	-0.4874477	-0.6487039
H	-1.5959471	-0.6103308	-2.3575843
H	4.0441714	-2.0039476	4.3105326
H	2.6140365	-0.9944232	4.5477229
H	3.9867903	-0.3797070	3.6217423
H	-0.1157357	-0.4938683	2.1098075
H	3.7189881	1.5120567	-0.4608838
H	4.3642373	1.3024645	1.1687946
H	4.0050197	2.9210250	0.5635613
H	3.6906672	-2.9024939	0.1326661
H	4.7642342	-3.1159002	1.5174117
H	4.6172097	-1.5328026	0.7496379
H	1.8853124	0.2521245	-2.3222863
H	1.3388611	-1.1624915	-3.2257773
H	2.6277268	-1.3220432	-2.0294493

[$(SiMe_3)_3Ge]^-$ (**9**$^-$)

C	1.5448675	1.7763516	3.6830504
Si	0.2171742	1.5014432	2.3141402
C	0.9977950	2.2931348	0.7377871
C	-1.2073779	2.7077222	2.7936762
Ge	-0.5325371	-0.8267124	2.3713038
Si	-2.3577915	-0.7402620	0.7442203
C	-2.8149872	-2.5033082	0.1149348
Si	1.2155647	-1.8825864	1.0236306
C	0.9795402	-3.7944685	1.0581427
C	2.9551466	-1.6087143	1.8061550
C	1.4659249	-1.4741162	-0.8450370
C	-3.9597135	-0.1213632	1.6181544
C	-2.2586263	0.3255401	-0.8603580
H	2.4381735	1.1746227	3.4946451
H	1.1530946	1.4799418	4.6599980
H	1.8502396	2.8278384	3.7398024
H	-0.8260753	3.7224920	2.9575012
H	-1.7021898	2.3780339	3.7113930
H	-1.9687329	2.7594642	2.0102303
H	0.0414268	-4.0862870	0.5779186
H	0.9438182	-4.1562945	2.0894060
H	1.7986222	-4.3090610	0.5419755
H	-3.7468374	-2.4867673	-0.4623274
H	-2.9463809	-3.1934958	0.9527920
H	-2.0304232	-2.9122849	-0.5276912
H	-3.8466512	0.9082495	1.9686764
H	-4.1814025	-0.7405810	2.4917993

H	-4.8240627	-0.1571664	0.9442914
H	3.7103508	-2.2360076	1.3181790
H	2.9449141	-1.8553782	2.8713760
H	3.2746497	-0.5670042	1.7136770
H	1.2594289	3.3443283	0.9119263
H	0.3068887	2.2501241	-0.1077705
H	1.9089379	1.7657632	0.4428439
H	1.6706442	-0.4107828	-0.9918446
H	0.5706357	-1.7175616	-1.4238697
H	2.3034463	-2.0443871	-1.2657693
H	-2.1319091	1.3845421	-0.6197045
H	-3.1722409	0.2233728	-1.4588274
H	-1.4098202	0.0278006	-1.4809352

[$(SiMe_3)_3GeH$] (**9**-H)

C	3.2869253	2.6019331	1.4584457
Si	2.1627996	1.9254114	0.0801125
C	3.0728415	2.0710510	-1.5862193
C	0.5930129	3.0007292	0.0086838
Ge	1.5952469	-0.3875100	0.5454796
Si	-0.3546848	-1.1634198	-0.6737201
C	-0.5935241	-3.0377472	-0.4371014
Si	3.4861150	-1.9004428	0.3883045
C	3.1056280	-3.5010297	1.3445872
C	5.0482349	-1.1137513	1.1407086
C	3.8459350	-2.3359314	-1.4301706
C	-1.9117903	-0.2779638	-0.0315433
C	-0.1642400	-0.8085657	-2.5349288

H	4.2214865	2.0414466	1.5277445
H	2.7925936	2.5481748	2.4312901
H	3.5374711	3.6499000	1.2665914
H	0.8602739	4.0502955	-0.1482539
H	0.0239427	2.9363592	0.9388690
H	-0.0656397	2.6999686	-0.8092114
H	2.2320473	-4.0158327	0.9389838
H	2.9095753	-3.2929089	2.3990877
H	3.9555368	-4.1883163	1.2902955
H	-1.5011938	-3.3720195	-0.9491846
H	-0.6920405	-3.2961856	0.6197230
H	0.2457247	-3.6058957	-0.8446811
H	-1.8457104	0.8035799	-0.1669752
H	-2.0636642	-0.4718493	1.0328125
H	-2.7986165	-0.6301928	-0.5673473
H	5.8793640	-1.8251581	1.1138718
H	4.8875384	-0.8268122	2.1823855
H	5.3569155	-0.2216965	0.5910911
H	1.1766242	-0.4114060	2.0479961
H	2.4652722	1.6917165	-2.4112338
H	4.0121897	1.5132681	-1.5801603
H	3.3086725	3.1178603	-1.8002744
H	4.0699035	-1.4437663	-2.0198099
H	2.9969913	-2.8398820	-1.8981121
H	4.7086125	-3.0054686	-1.4985698
H	-0.0739950	0.2621698	-2.7324381
H	-1.0374499	-1.1751663	-3.0829849
H	0.7193385	-1.2994518	-2.9494806

6. References

1) A. G. Brook, F. Abdesaken and H. Söllradl, *J. Organomet. Chem.* **1986**, *299*, 9-13.

2) U. Herzog, N. Schulze, K. Trommer and G. Roewer, *J. Organomet. Chem.* **1997**, *547*, 133.

3) H. Li, L. J. Hope-Weeks and C. Krempner, *Chem. Comm.* **2011**, *47*, 4117-4119.

4) H. Li, A. J. A. Aquino, D. B. Cordes, F. Hung-Low, W. L. Hase and C. Krempner, *J. Am. Chem. Soc.* **2013**, *135*, 16066-16069.

5) M. Schlosser and J. Hartmann, *Angew. Chem.* **1973**, *85*, 544-5.

6) G. C. Vougioukalakis, M. M. Roubelakis and M. Orfanopoulos, *J. Org. Chem.* **2010**, *75*, 4124-4130.

7) H. Nakamura, Y. Nakayama, H. Yasuda, T. Maruo, N. Kanehisa and Y. Kai, *Organometallics* **2000**, *19*, 5392–5399.

8) L. A. Carpino and A. C. Wu, PCT Int. Appl. **1991**, WO 9108190 A1 19910613.

9) G. M. Sheldrick, *SADABS* v2008/1, 2008.

10) G. M. Sheldrick, *Acta Crystallogr.*, **2008**, *A64*, 112-122.

11) A. D. Becke, *J. Chem. Phys.* **1993**, *98*, 5648.

12) A. Schäfer, H. Horne and R. Ahlrichs, *J. Chem. Phys.* **1992**, *97*, 2571-2577.

13) A. Schäfer, C. Huber and R. Ahlrichs, *J. Chem. Phys.* **1994**, *100*, 5829-5835.

14) A. E. Reed, R. B. Weinstock and F. Weinhold, *J. Chem. Phys*. **1985**, *83*, 735-46.

15) R. Ahlrichs, M. Bär, M.; Häser, H. Horn and C. Kölmel, *Chem. Phys. Lett*. **1989**, *162*, 165.

16) NBO, version 3.1 E. D. Glendening, A. E. Reed, J. E Carpenter and F. Weinhold Theoretical Chemistry Institute WISC-TCI, University of Wisconsin-Madison

17) F. Weigend and R. Ahlrichs, *Phys. Chem. Chem. Phys.*, **2005**, *7*, 3297-305.

18) F. Weigend, *Phys. Chem. Chem. Phys.* **2006**, *8*, 1057-65.

19) Gaussian 09, Revision **E.01**, M. J. Frisch, G. W. Trucks, H. B. Schlegel, G. E. Scuseria, M. A. Robb, J. R. Cheeseman, G. Scalmani, V. Barone, B. Mennucci, G. A. Petersson, H. Nakatsuji, M. Caricato, X. Li, H. P. Hratchian, A. F. Izmaylov, J. Bloino, G. Zheng, J. L. Sonnenberg, M. Hada, M. Ehara, K. Toyota, R. Fukuda, J. Hasegawa, M. Ishida, T. Nakajima, Y. Honda, O.

Kitao, H. Nakai, T. Vreven, J. A. Montgomery, Jr., J. E. Peralta, F. Ogliaro, M. Bearpark, J. J. Heyd, E. Brothers, K. N. Kudin, V. N. Staroverov, R. Kobayashi, J. Normand, K. Raghavachari, A. Rendell, J. C. Burant, S. S. Iyengar, J. Tomasi, M. Cossi, N. Rega, J. M. Millam, M. Klene, J. E. Knox, J. B. Cross, V. Bakken, C. Adamo, J. Jaramillo, R. Gomperts, R. E. Stratmann, O. Yazyev, A. J. Austin, R. Cammi, C. Pomelli, J. W. Ochterski, R. L. Martin, K. Morokuma, V. G. Zakrzewski, G. A. Voth, P. Salvador, J. J. Dannenberg, S. Dapprich, A. D. Daniels, Ö. Farkas, J. B. Foresman, J. V. Ortiz, J. Cioslowski, and D. J. Fox, Gaussian, Inc., Wallingford CT, **2009**.
